# Sustainable Germanium Recovery From Germanium Dioxide via Halogen‐Free Chemical Vapor Transport

**DOI:** 10.1002/chem.71263

**Published:** 2026-06-19

**Authors:** Julian Burkhart, Christian A. Bömer, Jana Milanovic, Victoria S. Drescher, Igor Atanasov, Moritz Maxeiner, Klaus Müller‑Buschbaum

**Affiliations:** ^1^ Institute of Inorganic and Analytical Chemistry Justus‐Liebig‐University Giessen Giessen Germany; ^2^ Center For Materials Research (LAMA) Justus‐Liebig‐University Giessen Giessen Germany

**Keywords:** chemical vapor transport, critical resources, germanium, hydrogen, recovery

## Abstract

Chemical vapor transport (CVT) and its potential for chemical recycling was studied for recovery of Ge from GeO_2_ and SiO_2_/GeO_2_ mixtures using hydrogen, a hydrogen‑argon mixture and natural gas. CVT occurred via the transport species GeO, yielding mixtures of Ge and GeO_2_. Natural gas enabled transport but led to deposition of carbon deteriorating separation. Pure hydrogen proved too strong as reducing agent, forming elemental Ge at the source, preventing CVT. Optimal performance was achieved by a hydrogen(10%)‑argon(90%) mixture in combination with water vapor, altogether moderating reduction strength, stabilizing the transport species, improving separation and resulting in a sustainable process. The hydrogen(10%)‑argon(90%)‐based CVT achieved a maximum Ge recovery of 86% within 30 min, higher flow rates resulted in 73% in only 10 min. Upscaling was done by a factor of three and maintained high Ge recovery of 83% in 30 min demonstrating scalability. To mimic Ge recovery from optical fibers and thus from SiO_2_, a SiO_2_/GeO_2_ mixture was successfully investigated, resulting in Ge recovery of 76% in 30 min with no simultaneous transport of Si. The process presents significant potential for Ge recovery from oxide feedstocks and could be adapted to secondary resources such as GeO_2_‐based optical fiber waste.

## Graphical abstract

Recovery of the critical element Ge from GeO_2_ and SiO_2_/GeO_2_ mixtures was investigated based on an endothermic chemical vapor transport via GeO, formed with natural gas or a hydrogen(10%)‑argon(90%) mixture and water vapor. (This picture was partly created with AI, engine: Adobe Firefly).

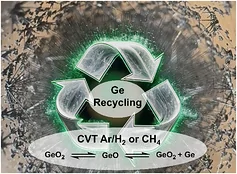

## 1 Introduction

Germanium's (Ge) electrical and optical properties make it crucial to various high‐tech applications, including optical fibers (≈ 30%–40%), infrared systems (≈ 25%), polymerization catalysts (≈ 25%), and electronic devices, such as solar cells or LEDs (≈ 10%–20%) [1, 2, 3]. The demand for these modern applications is expected to drive a significant increase in Ge consumption [1, 2, 4, 5]. In particular, the production of optical fibers to establish the fifth‐generation (5G) wireless telecommunication will further boost the demand for Ge [2, 4].

Unfortunately, Ge is regarded as one of the elements with the highest supply risk among the list of critical raw materials [6]. The primary reasons for the supply risk in Europe include its low crustal abundance (approx. 1.6 ppm) [2, 7, 8, 9], significant production losses during extraction from zinc refinery residues or coal fly ash [3, 10, 11], and the fact that production is concentrated in non‐European countries [1, 12].

Given the supply risk, it is essential to either improve Ge production from zinc refinery residues or coal fly ash, or to establish ways for an effective recycling process for secondary resources, such as used optical fibers or production residues. In both cases, a chemically sustainable approach that avoids the use of hazardous chemicals is preferable. Currently, Ge can be extracted through various methods depending on the starting material, such as residues from the zinc production, coal fly ash, or secondary resources [2, 4, 10, 12]. The most common methods are hydrometallurgical and involve leaching, solvent extraction or ion exchange techniques [2, 4, 10, 12]. However, these methods have notable disadvantages, including the use of large amounts of hazardous chemicals (e.g. sulfuric acid, hydrofluoric acid or sodium hydroxide), high costs, significant chemical consumption, and the co‐extraction of impurities [2, 4, 10, 12].

Another potential method for recovering Ge can be the use of the gas phase for separation, utilizing a so‐called chemical vapor transport (CVT) process (see Figure 1). Using CVT reactions as a potential recovery method for critical resources was recently reported for tellurides [13, 14, 15]. In general, CVT reactions are well‐known for their application in forming single crystals [16, 17] and are also used as a technical purification method, exemplified in the Mond or van‐Arkel‐de‐Boer processes [18, 19, 20, 21, 22]. CVT reactions can occur in closed or open systems. In closed systems, a chemical equilibrium is established, where the transport agent (TA) repeatedly reacts with the source material (SM) to form the transport species (TS), which moves along a temperature gradient. This results in the deposition of a purified compound (PC) at the sink [21, 22]. In an open system, equilibrium is not achieved, as the TA interacts with the SM only once before exiting as a gas or solid. Continuous TA supply in open systems allows for higher transport rates and faster recovery, which is highly favorable for industrial applications [23].

**FIGURE 1:**
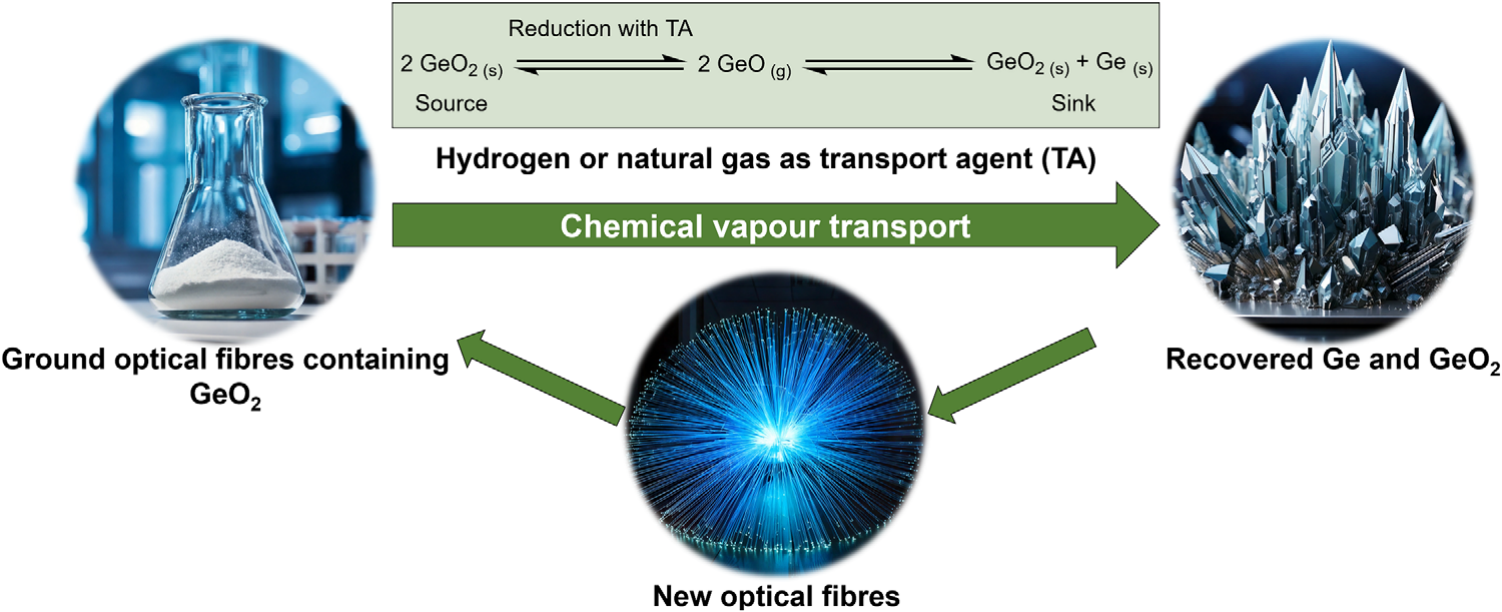
A schematic representation of a sustainable life cycle for Ge starting with GeO_2_‐containing secondary resources, such as optical fibers made from fused silica doped with Ge. The process involves a chemical vapor transport reaction using hydrogen, a hydrogen‐argon mixture or natural gas as the transport agent TA to form the transport species GeO. This ultimately results in a mixture of Ge and GeO_2_, which can be utilized to produce new materials. This picture was partly created with AI (engine: Adobe Firefly).

Specifically for the recovery of Ge, a CVT based on the volatile germanium monoxide (GeO) species is a promising recovery possibility suitable for all Ge resources based on germanium dioxide (GeO_2_). This would include coal fly ashes, optical fiber waste, as well as polymerization catalysts. Altogether, the volatile species GeO is mentioned in different ways in the literature: GeO is either described in CVT reactions performed with different transport agents [24, 25, 26, 27, 28, 29, 30, 31, 32], as an unfavorable side reaction during the reduction of GeO_2_ [33, 34, 35, 36], during chemical vapor deposition (CVD) methods to deposit Ge in different morphologies (thin films, needles, wires) [37, 38, 39, 40] or to separate Ge in processes with carbon [41, 42, 43]. Depending on the purpose of investigations, they were performed in closed and open systems, and for both cases different reduction agents were described in the literature that include hydrogen [24, 25, 26, 27, 28, 33, 34, 35, 36, 37], methane [38], alcohols [39], carbon [40, 41, 42, 43], and water [29, 30, 31, 32]. The formation of the well‐known volatile hydride species GeH_4_ or other hydrides is typically not considered in the literature due to its thermal decomposition already occurring at relatively low temperatures of approximately 400°C being outside process temperatures [44].

To date, only the reduction with carbon has been investigated with a focus on Ge recovery, whereby greenhouse gases are formed and reduced pressure is used [41, 42, 43]. The CVT reactions with natural gas or hydrogen as transport agents have not been investigated with a focus on Ge recovery, chemical recycling and sustainability concerns, what we would like to highlight with our study. Furthermore, to the best of our knowledge, no comparison of different reducing agents has been reported in the literature.

In this work, we describe CVT based on the volatile species GeO, being investigated as a new approach to recover Ge from Ge resources based on germanium dioxide (GeO_2_), like GeO_2_‐based optical fiber waste. Therefore, the CVT process was investigated with pure GeO_2_ and GeO_2_/SiO_2_ mixtures as starting material. Especially, transport has been developed for an open‐flow system allowing for a high increase of the Ge transport rate up to 35 times of the reported closed systems [24, 25, 26, 27, 28, 29, 30] and open systems [37, 38, 39], improving the feasibility of the process for chemical recycling. Furthermore, hydrogen and natural gas are compared as potential reducing agents in terms of effectiveness and sustainability. Optimal conditions for both reducing agents, including the possibility for upscaling, were determined by analyzing transport rates and recovery yields. In addition, the phase compositions and purity were identified using energy‐dispersive x‐ray spectroscopy (EDX) and powder x‐ray diffraction (PXRD).

## 2 Results and Discussion

To investigate the recovery of Ge from GeO_2_‐containing secondary resources, such as optical fibers, a model system using pure GeO_2_ as the source material was studied. Hydrogen and natural gas were selected as transport agents, and their performance was compared, because different from a halogen‐based transport, no co‐transport of silicon has to be expected [45, 46, 47]. Therefore, a halogen‐free chemical vapor transport reaction is beneficial for applications involving both Ge and Si oxide feedstock, such as optical fibers.

Chemical vapor transport (CVT) reactions can be carried out in either closed or open systems. In closed systems, a chemical equilibrium can be established, allowing the transport agent (TA) to repeatedly interact with the source material (SM) and form the transport species (TS). The transport species then moves according to the reaction type: from the higher temperature region (*T*_2_) to the lower temperature region (*T*_1_) for endothermic reactions, or in the opposite direction for exothermic reactions. The temperature difference at the sink shifts the equilibrium, driving the back‐reaction and resulting in the deposition of the transported material as a purified compound (PC) [21, 22].

In open systems [13, 14, 15], like the one used in this work (see Figure 2), the transport agent interacts with the source material only during contact before it is moved forward. Depending on the specific CVT reaction, the transport agent either exits the system as a gas or solidifies in cooler regions beyond the sink temperature (*T*_1_). Consequently, the transport agent must be continuously supplied, but can be reused via cycling. The flow rate *ν* (normal liter (NL, at 0°C and 1 bar) per hour and square centimeter), at which the transport agent is introduced can significantly affect the chemical equilibrium of the CVT reaction, enabling higher transport rates (as transported mass per hour) and, in turn, giving options for faster processes, which is preferable for recovery [23]. Due to these differences, open systems offer unique opportunities to control transport dynamics and enhance efficiency and process speed, making further investigations in this field crucial for developing new recycling strategies for critical elements like Ge.

**FIGURE 2:**
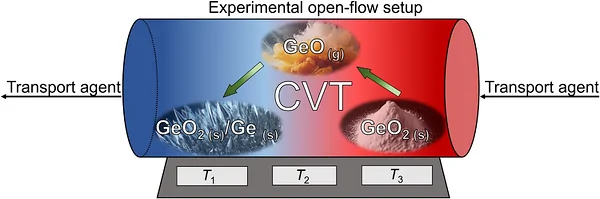
Schematic illustration of the experimental open‐flow CVT setup. At the source (located at T_3_), GeO_2_ (hexagonal) reacts with the transport agent and forms the transport species GeO, which evaporates and disproportionates at the sink (located at T_1_) into Ge and GeO_2_. This picture was partly created with AI (engine: Adobe Firefly).

### 2.1 Chemical Vapor Transport With Natural Gas

Chemical vapor transport reactions were investigated with natural gas (NG) as a reducing agent because of its abundance, and taking its influence regarding the “carbon footprint” into consideration. The natural gas used consisted primarily of methane (92%), followed by ethane (4%) and smaller amounts of nitrogen (1%), carbon dioxide (1%), and other alkanes. Since methane is the main component, it is considered the primary reducing agent. Temperature‐dependent, methane can thermally decompose into its elemental constituents (**R1**), producing hydrogen, which then acts as the reducing agent (**R2**). This complete pyrolysis requires temperatures above 1200°C for a significant yield and in only low quantities within the investigated temperature range (700‐1000°C) [48]. Alternatively, methane itself can directly function as the reducing agent (**R3**). These reaction pathways (**R2** and **R3**) were also described in previous studies [37, 38, 39]. In these studies, instead of a complete pyrolysis into the elements (**R1**), a possible decomposition of methane into different carbon hydrides and hydrogen has been proposed, based on the absence of carbon deposition [37, 38, 39]. Since the results of the present study show significant carbon deposition, which affects both the reaction outcome and the purity of the recovered Ge, R1 is expected to occur at least in small quantities.

(R1) $$C{H_{4}}_{\left(g\right)}⇌2{H_{2}}_{\left(g\right)}+C_{\left(s\right)}$$

(R2) $$\mathrm{Ge}{O_{2}}_{\left(s\right)}+{H_{2}}_{\left(g\right)}⇌{\mathrm{GeO}}_{\left(g\right)}+H_{2}O_{\left(g\right)}$$

(R3) $$\mathrm{Ge}{O_{2}}_{\left(s\right)}+0.25{{\mathrm{CH}}_{4}}_{\left(g\right)}⇌{\mathrm{GeO}}_{\left(g\right)}+0.5H_{2}O_{\left(g\right)}+0.25{{\mathrm{CO}}_{2}}_{\left(g\right)}$$

In closed systems, a common approach to estimate the temperature dependence of CVT reactions is by calculating the chemical equilibrium constant *K*. For proper CVT, a temperature where *K* = 1 should be selected. However, since this study utilizes open systems, chemical equilibrium is not achieved due to the continuous gas supply. As a result, the calculation of *K* can only provide a rough estimation of the operating temperatures and is primarily useful for comparing CVT reactions that may occur simultaneously.

Figure 3 illustrates the temperature dependence of the logarithmic chemical equilibrium constant *K* for two CVT reactions discussed above (**a**: **R2**, **b**: **R3**). The graphs in Figure 3 were calculated using the van't‐Hoff equation [49] based on the enthalpies and entropies given in the literature [50, 51, 52]. For both CVT reactions (**a**: **R2**, **b**: **R3**), a value of *K* = 1 is reached at approximately 900°C (**a** = 920°C, **b** = 880°C), indicating that both CVT reactions will occur simultaneously, and consequently, this temperature range was investigated for a proper CVT. However, due to the expected low amounts of hydrogen, reaction **R3** is, in the case of NG investigations, likely the predominant pathway for forming the gaseous transport species GeO.

**FIGURE 3:**
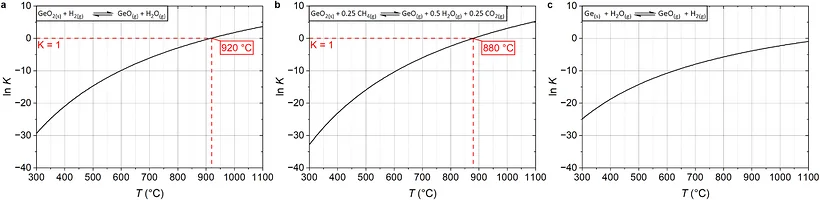
Logarithmic equilibrium constant of (a) reduction of GeO_2_ with hydrogen to GeO (**R2**), (b) reduction of GeO_2_ with methane to GeO (**R3**) and (c) oxidation of Ge with water to GeO (**R4**) in a temperature range of 300°C to 1100°C is shown. The logarithmic equilibrium was calculated using the van't‐Hoff equation [49] based on the enthalpies and entropies given in the literature [50, 51, 52].

According to these calculations, the CVT reactions were investigated within a source temperature range of 680°C–960°C (see Table 1) and in accordance with temperatures reported in the literature for systems using pure methane and ethanol‐argon mixtures as reducing agents [38, 39]. In all investigations, the transported material was firmly attached to the quartz tube after the CVT, either as a coating or as crystals (see Figures S3–S6). As a result, complete mechanical removal was complicated and may lead to an underestimation of yield and transport rate. However, the deposited material was mechanically removed as thoroughly as possible and analyzed using powder x‐ray diffraction (PXRD) and energy‐dispersive x‐ray spectroscopy (EDX).

**TABLE 1:** Temperature investigations of the CVT performed with NG (blue), H_2_ (orange) or Ar/H_2_ (green) as a reducing agents. In some cases, the reducing agent was passed through water. For each experiment, an identification (ID) abbreviation, the temperature gradient, the reaction time and the transport agent flow rate are listed. Additionally, the calculated recovery yield and transport rate are given.

ID	*T*_1_ (°C)	*T*_2_ (°C)	*T*_3_ (°C)	*t* (h)	*ν* (TA) (NL · h^−1^ · cm^−2^)	Water	*Recovery yield* (Ge) (%)	*Transport rate* (Ge) (mg · h^−1^)
NG‐I	760	930	960	0.5	3.1	No	76	321
NG‐II	760	930	960	0.5	3.1	Yes	75	320
NG‐III	660	830	860	0.5	3.1	Yes	63	269
NG‐IV	570	740	770	0.5	3.1	Yes	6	24
NG‐V	470	640	680	0.5	3.1	Yes	0	0
H_2_‐I	460	590	630	0.5	8.9	No	2	8
H_2_‐II	460	590	630	0.5	8.9	Yes	1	6
H_2_‐III	560	690	720	0.5	8.9	Yes	1	4
H_2_‐IV	650	790	820	0.5	8.9	Yes	2	7
ArH_2_‐I	760	930	960	0.5	2.2	No	88	377
ArH_2_‐II	860	1050	1060	0.5	2.2	Yes	36	152
ArH_2_‐III	760	930	960	0.5	2.2	Yes	86	366
ArH_2_‐IV	660	830	860	0.5	2.2	Yes	74	317
ArH_2_‐V	570	740	770	0.5	2.2	Yes	15	67
ArH_2_‐VI	470	640	680	0.5	2.2	Yes	2	8

In addition to the deposition of the transported material as coating or as crystals (see Figure 4), elemental carbon deposition was observed at source temperatures above 800°C. This carbon deposition was visible to the naked eye (see Figure s 4 & S3), and the elemental mapping revealed carbon deposition on the transported material (see Figures S19,S26). Consequently, contrary to previous reports for methane [38] and ethanol‐argon mixtures [39], pyrolysis must occur, at least to a certain extent. In general, this carbon deposition obstructs the optical assessment and thereby the separation between the source and sink.

**FIGURE 4:**
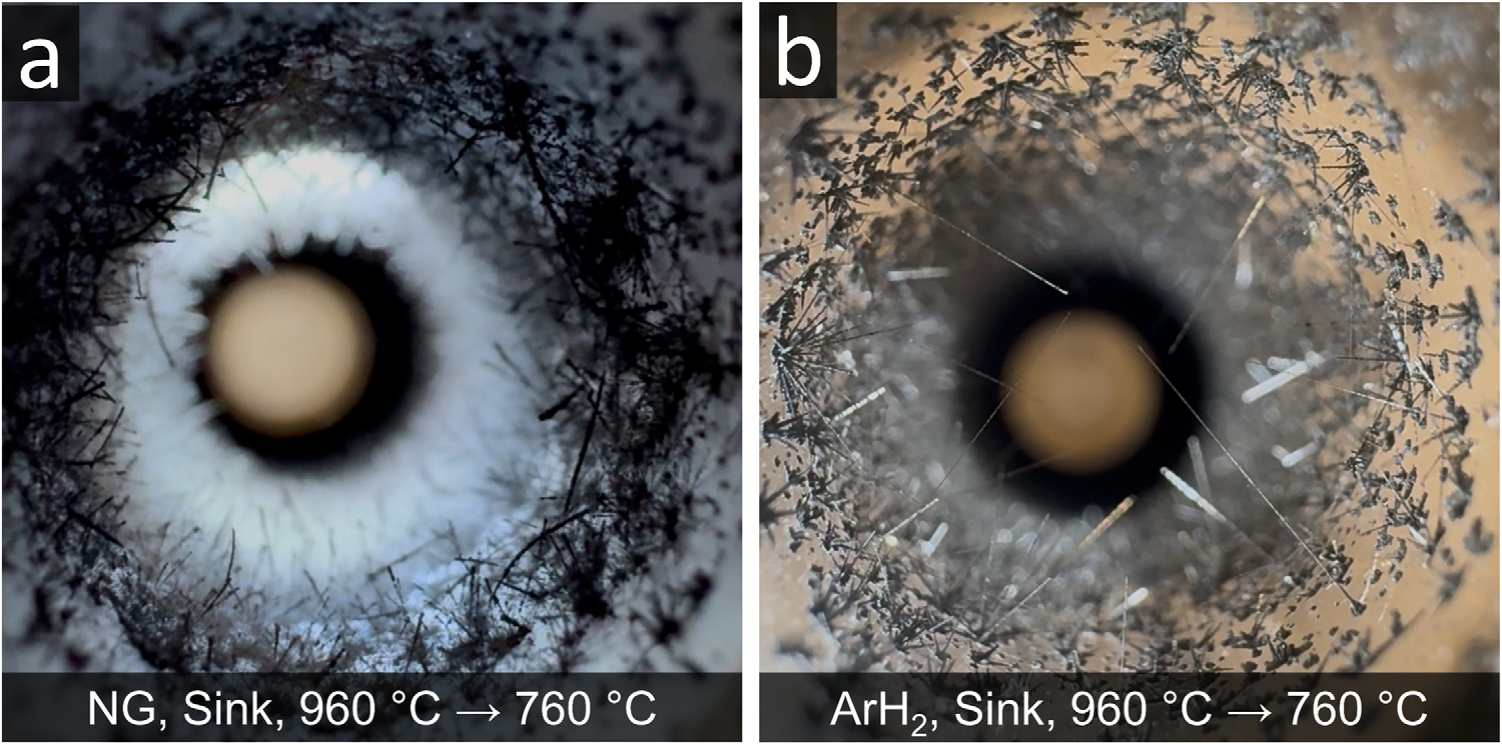
Images of the deposition at the sink. The CVT process was performed at a temperature gradient of 960°C → 760°C with natural gas (a) or a hydrogen(10%)‑argon(90%) mixture (b) as transport agent. In image a, the carbon deposition is visible to the naked eye.

The PXRD data of both the source and sink materials after the CVT reaction are shown in Figure 5. At the source, with increasing temperature, the hexagonal modification of GeO_2_ transforms to the thermodynamically more stable tetragonal modification. Additionally, at source temperatures of 860°C or higher, reflections corresponding to elemental Ge appear. This reduction to elemental Ge likely occurs either through reaction with methane or with carbon formed by the pyrolysis of NG. However, the presence of elemental Ge hinders the transportation of Ge, as its re‐oxidation to GeO (**R4**), as well as the complete re‐oxidation to GeO_2_ in the presence of water vapor, is less likely to occur (see Figures 3c and S1).

(R4) $${\mathrm{Ge}}_{\left(s\right)}+H_{2}O_{\left(g\right)}⇌{\mathrm{GeO}}_{\left(g\right)}+{H_{2}}_{\left(g\right)}$$

**FIGURE 5:**
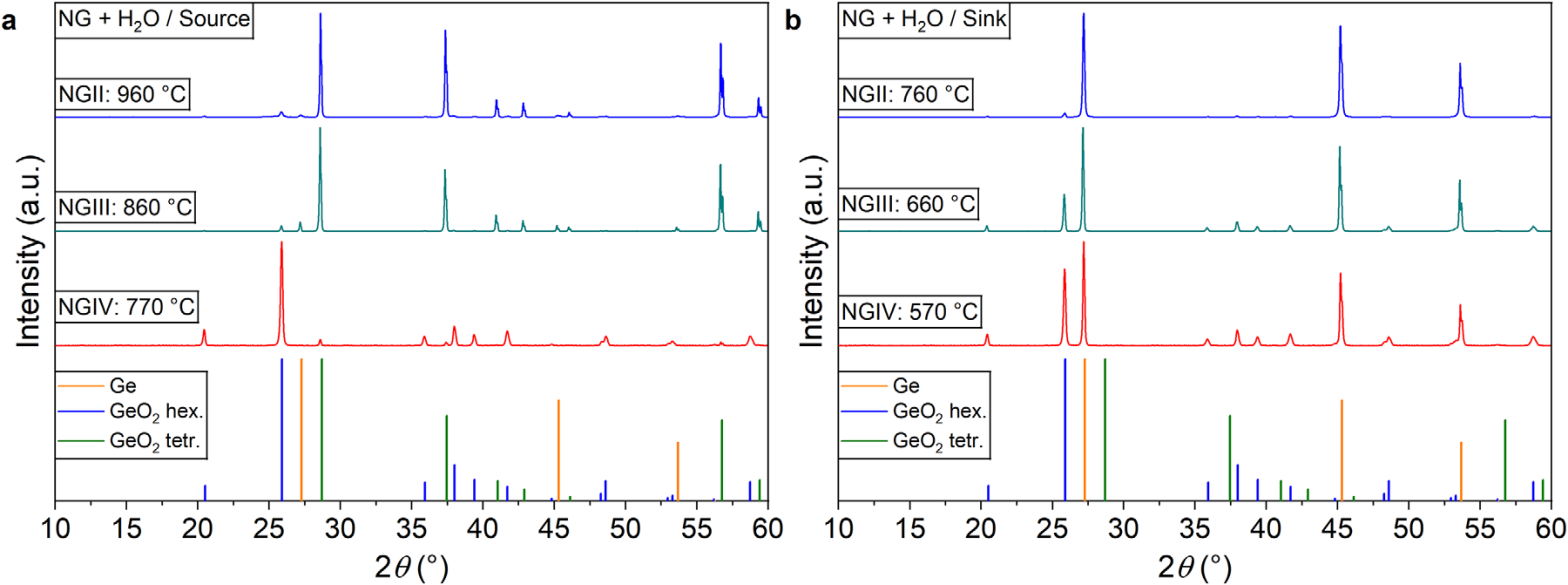
PXRD data of source (a) and sink (b) of the CVT experiments with NG and water vapor as transport agents are shown. Additionally, references for Ge [55], hexagonal GeO_2_ [56] and tetragonal GeO_2_ [57] are shown.

At the sink, a mixture of elemental Ge and hexagonal GeO_2_ is present at all temperatures, indicating that the transport species GeO disproportionates into Ge and GeO_2_ (**R5**), as described in the literature [40, 41, 53]. In addition, the temperature dependence of the logarithmic chemical equilibrium constant *K* of reaction **R5** indicates that disproportionation predominantly occurs below 600°C (see Figure S2), which corresponds well to the observed main deposition region (see Figures S5,6).

(R5) $$2{\mathrm{GeO}}_{\left(g\right)}⇌{\mathrm{Ge}}_{\left(s\right)}+\mathrm{Ge}{O_{2}}_{\left(s\right)}$$

This finding contrasts with reported CVD methods for Ge, in which pure elemental Ge is reported at the sink, which was linked to a second reduction of the transport species GeO by the transport agent [38, 39]. In the present work, the proportion of elemental Ge at the sink increases with temperature (see Figure 5b). Since this behavior is not observed in the hydrogen‐based investigations (see Figure 7b), we propose that the proportion of elemental Ge increases due to a second reduction of GeO or GeO_2_ with the deposited carbon rather than the transport agent. In general, a reduction of GeO_2_ by carbon has also been reported in the literature [40, 41, 42, 43]. In addition, we propose that a second reduction of GeO with the transport agent would also be expected to occur at higher temperatures closer to the source. This reduction to Ge would, in turn, prevent successful transport to the sink and therefore cannot explain the increasing amount of Ge observed at the sink.

Removal of the whole transported material was achieved via dissolving and quantified by microwave plasma atomic emission spectroscopy (MP‐AES). Since hexagonal GeO_2_ is soluble in water (4.05 g · L^−1^, 387 mmol · L^−1^) [54], and any elemental Ge present needs to be oxidized, the transported material at the sink was dissolved in a water:hydrogen peroxide mixture (80:1) for removal. The carbon deposition hinders the dissolution of the transported material in the water:hydrogen peroxide mixture and needs to be removed. For removal, the carbon was oxidized to CO_2_. The oxidation is occurring in a temperature range of 850–900°C, which was previously determined in a synthetic air atmosphere using simultaneous thermal analysis (differential thermal analysis + thermogravimetry) coupled to a mass spectrometer (DTA/TG‐MS) (see Figure S9). In the end, at a temperature of 925°C, carbon could be completely removed. The complete removal is evidenced by the elemental mapping of carbon, which, after its oxidation, shows carbon only in the carbon pad area, not deriving from the sample (compare Figure 6c,d), and is further supported by optical assessment of the reaction tubes (compare Figures S3, S4).

**FIGURE 6:**
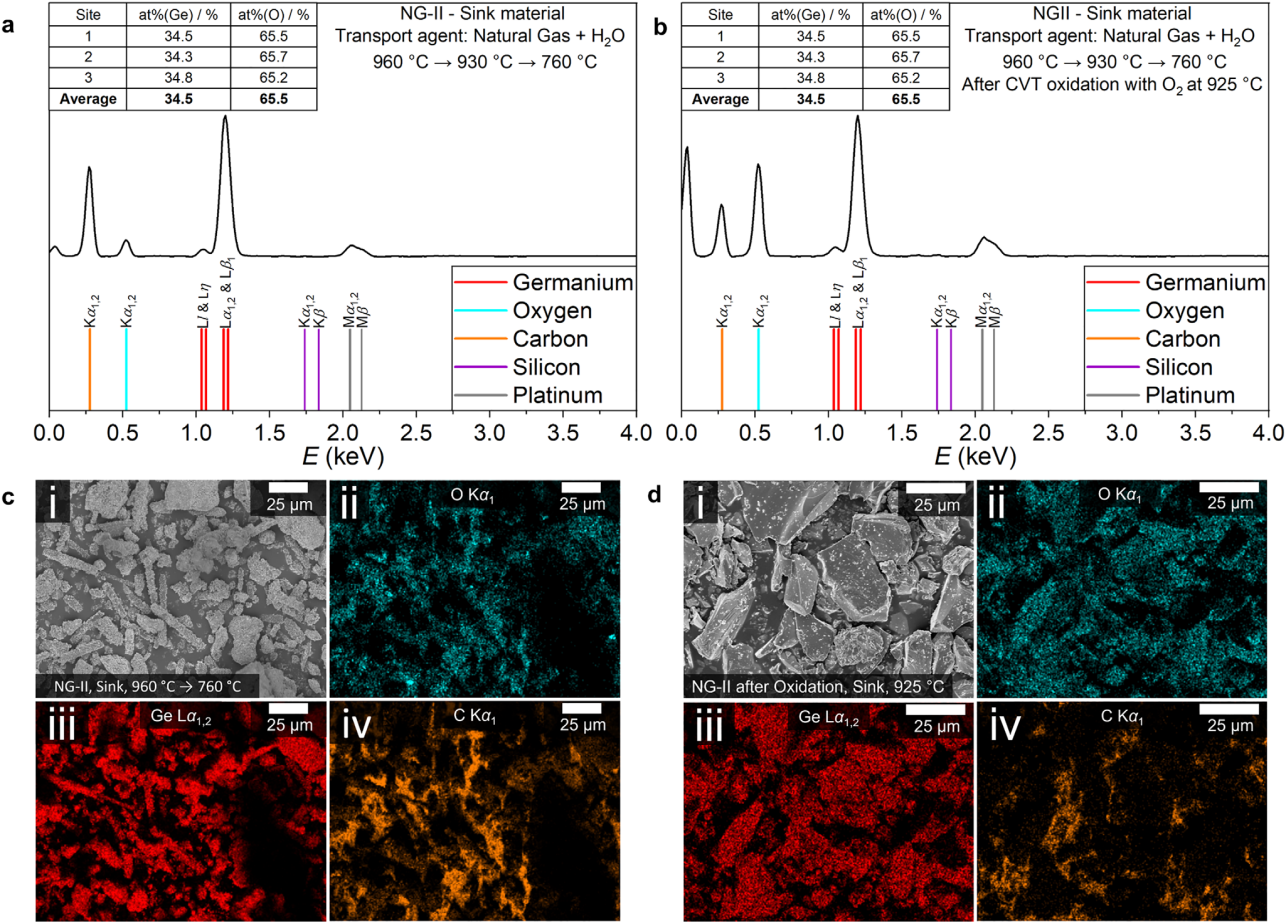
EDX data of the sink of a process with natural gas, NGII‐sink (960°C → 760°C) before (a) and after oxidation at 925°C (b) are shown. For each sample, an average at% value of germanium and oxygen is given. It is calculated from three different measuring spots. Characteristics x‐ray emission lines are shown for germanium, oxygen, carbon, silicon and platinum as references [58]. Additionally, a scanning electron microscopy (SEM) image, and the elemental mapping for germanium (red dots), oxygen (blue dots) and carbon (orange dots) are shown before (c) and after the oxidation (d) of the sink material. Experiment designation according to Table 1.

During the carbon removal process, most of the elemental Ge also oxidized (see Figures 6a,b, and S8) and could therefore be transported again (**R6**), preventing a final assessment of the separation between the source and sink after CVT.

(R6) $$2{\mathrm{Ge}}_{\left(s\right)}+{O_{2}}_{\left(g\right)}\to 2{\mathrm{GeO}}_{\left(g\right)}+{O_{2}}_{\left(g\right)}\to 2\mathrm{Ge}{O_{2}}_{\left(s\right)}$$

After carbon removal, the sink material was dissolved in a water:hydrogen peroxide mixture, as described above, and the Ge concentration $c_{\mathrm{MP}-\mathrm{AES}}(Ge_{\mathrm{Sink}})$ in the solution volume $V_{\mathrm{MP}-\mathrm{AES}}$ was determined by MP‐AES. The final recovery yields and transport rates were calculated using equations **E1** and **E2** in combination with the theoretically maximum recoverable amount of Ge $m\;(Ge_{\mathrm{Theoretical}})$. The transport rate was calculated by normalizing the experimentally transported Ge mass to the corresponding reaction time and is therefore reported in units of mg h^−^^1^. Since the transport reactions proved to be fast in the open flow systems with reaction times typically shorter than 1 h (mostly 30 min), the reported values represent extrapolated hourly rates and may numerically exceed the total Ge mass initially present in a single experiment.

(E1) $$\mathrm{Recovery}\;\mathrm{yield}=\frac{c_{\mathrm{MP}-\mathrm{AES}}(Ge_{\mathrm{Sink}})\;\cdot \;V_{\mathrm{MP}-\mathrm{AES}}}{m\;(Ge_{\mathrm{Theoretical}})}\;\cdot \;100\%$$

(E2) $$\mathrm{Transport}\;\mathrm{rate}=\frac{c_{\mathrm{MP}-\mathrm{AES}}(Ge_{\mathrm{Sink}})\;\cdot \;V_{\mathrm{MP}-\mathrm{AES}}\;}{Reaction\;time}$$

The calculated results are presented in Table 1. It is important to note that the hexagonal GeO_2_ used contained approximately 2.5 wt% moisture, as determined by DTA/TG‐MS (see Figure S8). Similar values of moisture were also reported in the literature [36, 38, 39]. Therefore, for calculating recovery yields and transport rates, the initial starting mass was corrected by a value of 2.5%.

During the investigations with hydrogen, the introduction of additional water vapor (≈ 5 vol%) resulted in a clear separation of the source and the sink, which was not observed without water vapor (see Figure S6). As noted above, the separation between the sink and the source was not observable during investigations with natural gas due to carbon deposition.

Nevertheless, to allow a better comparison between the reducing agents and to assume improved separation in the case of natural gas investigations, additional water vapor was introduced. Moreover, in the presence of natural gas, water vapor may promote a steam reforming process, potentially increasing hydrogen production and reducing carbon deposition [59, 60].

In general, the chemical equilibrium of **R2** and **R3** is not sufficiently influenced by temperature control, resulting in no separation of source and sink. Adding water vapor to the system shifts the chemical equilibrium of **R2** and **R3** toward the reactants, which in turn decreases the reducing power of the reducing agents, hinders a complete reduction to elemental Ge (**R7**) and therefore ultimately stabilizes the transport species GeO. This successfully shifts the deposition of the sink material and separates the transported material from the material at the source. As a further consequence of the stabilized transport species, the area of the deposited material (sink) is now adjustable with the applied temperature gradient (see Figure S6), which is advantageous for industrial applications.

The highest recovery yield of 76% in only 30 min process time (321 mg · h^−1^) was obtained at a temperature gradient of 960°C → 930°C → 760°C without the addition of water vapor. Introducing water vapor reduced the recovery yield slightly by 1%, which can be attributed to a slower formation of GeO (reactions **R2** and **R3**). Since the yield decline is minimal while the separation improves significantly (see Figure S6), the use of water vapor is considered essential. The influence of the water vapor was also investigated by PXRD and EDX (see Figures S11, S16). Lower temperatures at both source and sink (860°C → 830°C → 660°C) led to slower transport reactions and, consequently, lower recovery yields (63% instead of 75%). Nevertheless, such lower temperatures (860°C → 830°C → 660°C) may be considered beneficial for energy‐saving reasons and for reducing the amount of carbon impurities. Higher temperatures than 960°C are not desirable, as they increase the amount of carbon and promote more frequent reduction with carbon.

### 2.2 Chemical Vapor Transport With Hydrogen

Chemical vapor transport reactions were carried out with pure hydrogen (H_2_) and a hydrogen(10%)‑argon(90%) mixture (Noxal, Ar/H_2_) as reducing agents. In both cases, the CVT process relies on the formation of the volatile species GeO, as described by reaction **R2**.

Pure hydrogen was employed as a reducing agent at three temperature gradients. Since hydrogen and an Ar/H_2_ mixture do not cause carbon deposition, the oxidation step of carbon at 925°C can be omitted, resulting in a recovery process with shorter reaction time, less energy requirement and no CO_2_ formation. This beneficial side effect enhances sustainability while reducing costs for energy in comparison to the NG recovery process. As described for the NG‐investigations, the transported material was dissolved in a water:hydrogen peroxide mixture (80:1) and the concentration to calculate recovery yields and transport rates was determined by MP‐AES (see Table 1) using equations **E1** and **E2**.

Furthermore, also for pure hydrogen as a reducing agent, water vapor was introduced for a better comparison with the other investigations (NG and Ar/H_2_).

None of the investigated temperature gradients using pure hydrogen as the reducing agent resulted in a significant transport of Ge (see Table 1). Instead, after reduction of GeO_2_ to GeO (**R2**) at the source, the transport species GeO was directly reduced to elemental Ge according to reaction **R7**. The reduction was confirmed by PXRD and EDX analyses of the source material (see Figures S12, S29, and S30).

(R7) $${\mathrm{GeO}}_{\left(g\right)}+{H_{2}}_{\left(g\right)}⇌{\mathrm{Ge}}_{\left(s\right)}+H_{2}O_{\left(g\right)}$$

The reduction capability of pure hydrogen is too high to achieve separation of Ge from the source. Even the addition of water vapor to the system does not sufficiently shift the reaction equilibrium of reaction **R7**. Under reducing conditions with hydrogen, volatile Ge–hydride species can also be considered to improve the transport situation, as in particular, GeH_4_ is known to be a volatile germanium hydride. However, under the experimental conditions employed in this work, GeH_4_ would thermally decompose (expected for temperatures below approximately 400°C [44]), and since no significant transport of Ge was observed, we assume that the volatile hydride species GeH_4_ does not positively influence the CVT process. Consequently, a recycling process could not be carried out using pure hydrogen.

As an alternative, an Ar/H_2_ mixture was investigated as a potential reducing agent with a lower reduction capability, preventing the complete reduction to Ge at the source (**R7**). From a safety perspective, the investigated Ar/H_2_ mixture, technically known as Noxal, only contains 10% hydrogen, which lowers the explosion risk considerably. Even in the event of a leak, the resulting gas‐air composition will typically remain below the lower explosion limit of hydrogen in air (≈ 4%). In addition, the higher density of the Ar/H_2_ mixture compared to air prevents oxygen diffusion into the tube, which is different from using hydrogen or NG. Overall, the use of the Ar/H_2_ mixture enhances the safety of the process compared to pure hydrogen, which is advantageous for the implementation of such a process in industrial applications. Although a CVT occurred, no distinct separation between the source and the sink was observed. Instead, deposition occurred along the whole reaction tube (see Figure S6). To address this, water vapor (≈ 5 vol%) was introduced to shift the reaction equilibrium **R7** toward the transport species GeO. In addition, the reaction equilibrium of **R2** and **R3** is shifted toward the reactants, which is resulting in a slower formation of the transport species GeO. This slower formation explains the slightly reduced recovery yields by 1–2% while using water vapor, observed for NG and Ar/H_2_ investigations. Most importantly, the introduction of water vapor markedly improved the separation between source and sink and is beneficial for an effective recovery process (see Figure S6). The effect of the water vapor was also investigated by PXRD and EDX (see Figures 7c,d, S11, and S16). The results of the investigations with Ar/H_2_ as a reducing agent are shown in Table 1.

**FIGURE 7:**
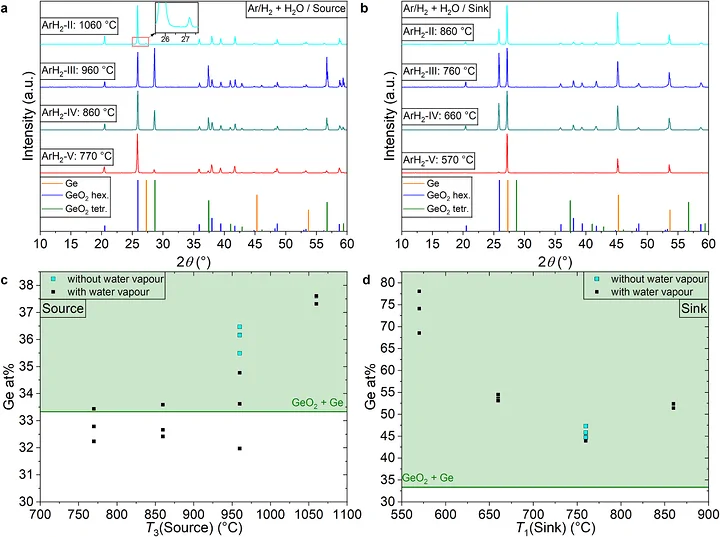
PXRD and EDX data of source (a & c) and sink (b & d) of the CVT experiments with Ar/H_2_ without (c & d, turquoise boxes) and with (c & d, black boxes) water vapor as transport agents are shown. Additionally, PXRD references for Ge [55], hexagonal GeO_2_ [56] and tetragonal GeO_2_ [57] are shown.

The highest recovery yield of 88% in only 30 min process time (377 mg · h^−1^) was achieved at a temperature gradient of 960°C → 930°C → 760°C without the addition of water vapor. Similarly to NG investigations, also for the Ar/H_2_ investigations, water vapor slightly reduced the recovery yield by 2%, which can again be attributed to a slower formation of GeO (**R2**). As previously mentioned, the decrease is minimal, while the improvement in separation is significant (see Figure S6), confirming that the use of water vapor is essential and beneficial.

The optimal temperature gradient (960°C → 930°C → 760°C) for the Ar/H_2_ investigations corresponds to the optimal conditions observed in the NG investigations (NG‐I), with a 12% higher recovery yield. In general, the recovery yields at each temperature gradient were consistently the highest when using Ar/H_2_ as the reducing agent. This improvement in recovery yields in all Ar/H_2_ investigations compared to the NG investigations may be due to the possible reduction of GeO_2_ or GeO to elemental Ge by carbon, which impedes efficient CVT and can only occur when using NG as the reducing agent.

Furthermore, as observed for the NG investigations (see Table 1), lower temperatures at both the source and the sink (860°C → 830°C → 660°C) led to slower transport reactions and, consequently, lower recovery yields (74% instead of 86%). Nevertheless, such reduced temperatures may still be advantageous for energy‐saving reasons.

Additionally, higher temperatures (1060°C → 1050°C → 860°C) were investigated for Ar/H_2_, which led to a significant decrease in the recovery yield to only 36%. The source material appeared slightly greyish and was no longer powdery, instead appeared as a hard chunk. This suggests that complete reduction to elemental Ge occurs more frequently at elevated temperatures, which was confirmed by EDX and PXRD. EDX revealed a higher Ge content of approx. 37.5 at% compared to approx. 33.3 at% at the other temperature gradients (see Figure 7c). In addition, PXRD showed a small reflection at 27.2 °, which was not present at the other temperature gradients and corresponds to the strongest reflection of Ge (see Figure 7a). Therefore, the source materials for process temperatures 960°C (Ar/H_2_‐III) and for 1060°C (Ar/H_2_‐II) were also investigated with SEM and EDX in order to check for potential morphological and element distribution differences (see Figure S46). The data strongly suggest that, for 1060°C, the formed elemental Ge quantitatively melts and encapsulates GeO_2_ within the molten Ge matrix (mp: 938°C [61]). This becomes evident when comparing both the surface and the fracture surface of the chunk‐like source material obtained at 1060°C with the powdery source material at 960°C (see Figure S46). This encapsulation impedes the contact between GeO_2_ and hydrogen, significantly slowing down the CVT process or possibly stopping it entirely. However, since both temperatures of 1060°C and 960°C exceed the melting point of Ge, also for the lower process temperature melting can be expected. Accordingly, we expect parameters such as the ratio difference of Ge/GeO_2_ formed and the fluidity of a Ge melt to be of further influence.

Furthermore, for Ar/H_2_, the reaction time and the flow rate were investigated with respect to recovery yield and transport rate at the optimal temperature gradient of 960°C → 930°C → 760°C (see Tables S1 and S2). A reaction time of only 30 min was required to completely transport the source material. To investigate the flow rate, a reaction time of 10 min was used. A flow rate lower than 2.2 NL · h^−1^ · cm^−2^ (recovery yield: 48%) resulted in a reduced recovery yield (30%), while higher flow rates resulted in faster recovery, achieving a plateau (72%) at a flow rate of 4.4 NL · h^−1^ · cm^−2^. Therefore, a flow rate range of 2.2‐4.4 NL · h^−1^ · cm^−2^ is proposed as the optimum, balancing recovery yield and chemical consumption.

In general, the composition of the source and sink materials differs between the NG and Ar/H_2_ investigations. The varying composition can be attributed to the presence of carbon, which is only formed when NG is used and resulted in a second reduction of GeO and GeO_2_ to elemental Ge. This difference is evident when directly comparing PXRD and EDX data for both reducing agents (see Figure S52).

At the source, for both NG and Ar/H_2_, increasing temperature causes a transformation of the hexagonal modification of GeO_2_ to the thermodynamically more stable tetragonal modification, except in the case of 1060°C (only investigated for Ar/H_2_), where the hexagonal modification of GeO_2_ remains. This may again be linked to the formation of a passivation layer of elemental Ge, which was described above. Additionally, the formation of elemental Ge at source temperatures of 860°C or higher in the NG‐based investigations is absent when using Ar/H_2_, which can be attributed to the absence of carbon. The absence of elemental Ge in the case of Ar/H_2_ is highly beneficial and likely contributes significantly to the higher recovery yields observed.

At the sink, for both reducing agents, a mixture of elemental Ge and hexagonal GeO_2_ is consistently present across all temperatures, indicating that the transport species GeO disproportionates into Ge and GeO_2_ (**R5**), as corroborated by the literature [40, 41, 53]. However, the sink composition still differs notably between NG and Ar/H_2_. For NG, the amount of elemental Ge increases with temperature due to a second reduction by carbon at the sink. In contrast, for Ar/H_2_, the amount of elemental Ge initially decreases until 760°C and afterwards increases again at higher temperatures (860°C), due to a second reduction with hydrogen. This overall observation confirms that in the case of NG, the second reduction can only be attributed to carbon and not to the original reducing agent itself. This contrasts with previous reports, where no carbon deposition was reported, and the second reduction was attributed to the original reducing agents only [38, 39].

In addition, the general feasibility of upscaling the process was demonstrated by tripling the amount of source material and increasing the overall reaction setup by enlarging the tube diameter to accommodate a larger amount of starting material while keeping other parameters constant (960°C → 930°C → 760°C, 2.2 NL · h^−1^ · cm^−2^). This upscaling resulted in a recovery yield of 83% in only 30 min of process time and a transport rate of 1055 mg · h^−^^1^. It should be noted that these experiments were intended as a proof‐of‐concept and feasibility demonstration rather than a systematic parametric study. Consequently, the effects of increased reactor dimensions, altered diffusion conditions, and increased source material loading cannot be considered independently. Nevertheless, the observed increase in transport rate with larger source material quantities indicates that higher amounts of Ge can generally be transported within comparable process times, highlighting the potential of the process for future scale‐up applications.

PXRD and EDX analyses of the sink material showed results identical to those of the standard (ArH2‐III), as presented in the supporting information (see Figures S14, S42).

Finally, the Ge recovery from a SiO_2_/GeO_2_ (15 wt% Ge) mixture was investigated to simulate a potential Ge recovery from GeO_2_‐based optical fiber waste involving larger amounts of fused silica (see Figure 8). EDX determined a Ge content of 16.6 wt% in the SiO_2_/GeO_2_ starting mixture, which is close to the experimentally used value of 15 wt% (see Figure 8b). The slightly higher value of Ge arises because larger silica particles were avoided during EDX measurements to prevent focusing issues.

**FIGURE 8:**
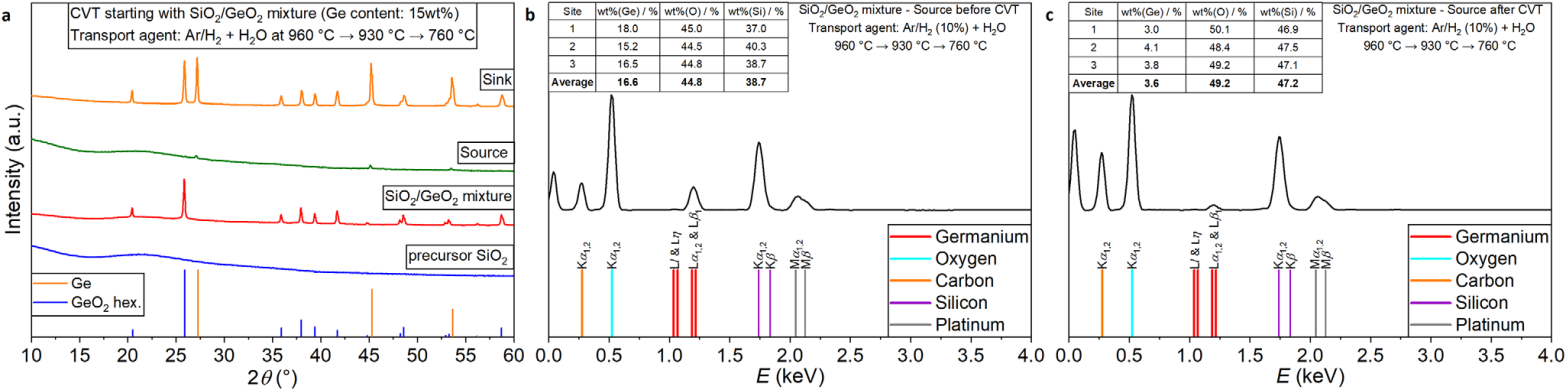
PXRD data (a) of the used SiO_2_ precursor (blue), the SiO_2_/GeO_2_ starting mixture (red), the source (green) and the sink (orange) after the recovery process. PXRD references for Ge [55] and hexagonal GeO_2_ [56] are shown. Additionally, EDX data of the source before (b) and after (c) the CVT process are shown. For each sample, an average at% value of germanium, oxygen and silicon is given. It is calculated from three different measuring spots. Characteristic x‐ray emission lines are shown for germanium, oxygen, carbon, silicon, and platinum as references [58].

Since the silica is amorphous, the XRD pattern shows only GeO_2_ reflections (Figure 8a). For improved visibility of the amorphous fraction, the data in Figure 8a was not background corrected. The best performing Ar/H_2_ process setup (960°C → 930°C → 760°C, 2.2 NL · h^−1^ · cm^−2^) was used for this recovery investigation. Within only 30 min process time, a recovery yield of 76% (149 mg · h^−1^) was achieved, being comparable with pure GeO_2_ (86%), demonstrating the promising process applicability to GeO_2_‐based optical fiber waste. XRD and EDX confirmed that the phase composition of the sink consists of Ge and GeO_2_ (Figures 8a and S43), similar to the investigation using pure GeO_2_ (see Figure 7b) as starting material. The source composition differs in comparison to the investigation using pure GeO_2_ as starting material. At the source of the SiO_2_/GeO_2_ investigation, small reflections of elemental Ge are visible (see Figure 8a). EDX determined a remaining Ge content of 3.6 wt% (see Figure 8c). The reflections of elemental Ge were absent using pure GeO_2_ (see Figure 7a), likely due to the high crystallinity of remaining GeO_2_ in comparison to the remaining amorphous silica at the source. Therefore, we recommend decreasing the hydrogen content or increasing the water vapor concentration to suppress elemental Ge formation at the source for materials and mixtures of SiO_2_/GeO_2_. Furthermore, EDX revealed a silicon content (1.1 wt%) in the recovered sink material similar to all other samples, attributed to small silica particles from the dismantling of the reaction tube, as described in the supporting information (see Figures S47–S51). Consequently, no simultaneous transport of Si occurred, which in turn shows the strong potential of this process as a technically relevant Ge recovery method.

In comparison to hydrometallurgical recycling routes [2, 12], which already achieve recovery rates close to 100% but are often associated with limited sustainability, the investigated CVT recycling strategy could offer a selective and sustainable approach for Ge recovery while achieving comparable recovery rates. In particular, the CVT‐based recycling method avoids the use of hazardous chemicals such as sulfuric acid, hydrofluoric acid, and sodium hydroxide. Moreover, hydrometallurgical recycling routes typically require large amounts of such chemical reagents, whereas the proposed process could operate with minimal reagent consumption, if the unreacted reducing gas is recovered and recirculated within the system. In addition, when operated using green energy and possibly green hydrogen for the Ar/H_2_ flow, the process has the potential to be fully sustainable without direct greenhouse gas emissions. Also, as different from halogen containing transport processes, no transport of Si‐containing species is involved, selectivity for systems with mixed silicon oxide—germanium oxide constitutions is high, with the achievable purity being not yet fully explored.

Overall, these findings highlight the significant potential of CVT processes as a recovery method in open setups, and in particular, introduce a novel approach for Ge recovery from oxide species, for example, in a recycling treatment of GeO_2_‐based optical fiber waste.

## 3 Conclusion

In this work, a comparative study of a halogen‐free CVT‐based recovery process for Ge from GeO_2_ using different reducing agents (natural gas, H_2_ and Ar/H_2_) in a continuous flow process is presented. All CVT processes occurred via the transport species GeO, yielding a mixture of Ge and GeO_2_ at the sink, with their relative amounts depending on the temperature and the reducing agent, confirmed by PXRD and EDX analyses. Furthermore, the presence of water vapor (≈ 5 vol%) proved essential for shifting the reaction equilibrium of the CVT reaction. The water vapor hinders the formation of elemental Ge at the source, in turn, stabilizes the transport species GeO, and especially promotes effective separation of the sink and the source.

In terms of both separation and recovery yield, the most effective natural gas‐based CVT recovery process (960°C → 930°C → 760°C, 3.1 NL · h^−1^ · cm^−2^, 30 min, ≈ 5 vol% H_2_O) achieved a recovery yield of 75% in only 30 min process time (320 mg · h^−1^). However, due to carbon formation and deposition throughout the entire tube, an oxidation step at 925°C was required to remove the carbon as CO_2_. Moreover, the formed carbon promoted the reduction of GeO_2_ to Ge at both sink and source. In particular, reduction of GeO_2_ to Ge at the source proved detrimental, as it hindered the formation of a stable transport species GeO and therefore reduced the recovery process efficiency.

When pure hydrogen was used as a reducing agent, no significant CVT recovery was observed (recovery yield 2%); instead, the source material GeO_2_ was completely reduced to elemental Ge.

To control and reduce the reduction capability of pure hydrogen, a hydrogen(10%)‑argon(90%) (Noxal, Ar/H_2_) mixture was employed instead. The most effective Ar/H_2_‐based CVT recovery process (960°C → 930°C → 760°C, 2.2 NL · h^−1^ · cm^−2^, 30 min, ≈ 5 vol% H_2_O achieved a recovery yield of 86% in only 30 min process time (366 mg · h^−1^).

Furthermore, scaling up the process by tripling the source material and increasing the tube diameter yielded a recovery yield of 83% in only 30 min process time and a proportional increase in the transport rate to 1055 mg · h^−1^, showing that the process is scalable. This finding underlines the substantial potential of the presented CVT recovery method for Ge, as well as the broader applicability of CVT processes as future recovery technologies.

Ultimately, recovery from a SiO_2_/GeO_2_ (15 wt% Ge) mixture was investigated, resulting in a high Ge recovery of 76% (149 mg · h^−1^) also in only 30 min process time. Importantly, no simultaneous transport of Si occurred, which enables the use of the presented process for the industrial recycling of GeO_2_‐based optical fiber waste, preferable to a halogen‐based CVT.

In conclusion, the Ar/H_2_ mixture not only delivered the highest overall recovery yield but also avoided the use of halogens as well as use and production and release of greenhouse gases and required less energy than the natural gas‐based CVT illustrating the advantages of an Ar/H_2_ mixture over natural gas or pure hydrogen.

Altogether, these findings highlight the significant potential of CVT processes as a recovery method in open flow setups avoiding the high consumption of hazardous chemicals of hydrometallurgical processes. In particular, this work introduces a novel and sustainable approach for germanium recovery from oxide species being free of halogens, which should be considered for future recovery processes, for example, in the treatment of GeO_2_‐based optical fiber waste. Operated with green energy and green hydrogen applied in a cycled flow‐process, this approach can constitute a fully sustainable process for the chemical recycling of Ge.

## Conflicts of Interest

The authors declare no conflicts of interest.

## Supporting information



The supporting information contains the experimental section and images of the reaction tubes. Additionally, further tables with transport rates and recovery yields are listed. Furthermore, thermal analysis measurements, PXRD diffractograms, EDX spectra and EDX elemental mappings are shown for several samples.

## Data Availability

The data that supports the findings of this study are available in the supplementary material of this article
