# Supplementary material for: Sustainable Germanium Recovery From Germanium Dioxide via Halogen‐Free Chemical Vapor Transport
Source: Chemistry. 2026 Jun 19;32(37):e71263. doi: 10.1002/chem.71263 (PMC13628529; doi:10.1002/chem.71263)
Supplement: Supplementary file 1 [file CHEM-32-e71263-s001.pdf]

Sustainable Germanium Recovery from Germanium Dioxide via Halogen-free Chemical Vapour Transport

Julian Burkhart[a], Christian A. Bömer[a], Jana Milanovic[a], Victoria S. Drescher[a], Igor Atanasov[a], Moritz Maxeiner[a], Klaus Müller-Buschbaum*[a, b]

[a] J. Burkhart, C. A. Bömer, J. Milanovic, V. S. Drescher, I. Atanasov, Dr. M. Maxeiner, Prof. Dr. K. Müller-Buschbaum
Institute of Inorganic and Analytical Chemistry
Justus-Liebig-University Giessen
Heinrich-Buff-Ring 17, 35392 Giessen, Germany
E-mail: klaus.mueller-buschbaum@anorg.chemie.uni-giessen.de
Homepage: https://www.uni-giessen.de/de/fbz/fb08/Inst/iaac/mueller-buschbaum

[b] Prof. Dr. K. Müller-Buschbaum
Center for Materials Research (LAMA)
Justus-Liebig-University Giessen
Heinrich-Buff-Ring 16, 35392 Giessen, Germany.

Contents

1. Experimental section 2
2. Chemical vapour transport (CVT) 4
3. Microwave plasma atomic emission spectroscopy (MP-AES) 8
4. Simultaneous thermal analysis (STA) 9
5. Powder X-ray diffractometry (PXRD) 10
6. Energy dispersive X-ray analysis (EDX) 13
7. Comparison natural gas and hydrogen(10%)-argon(90%) mixture 27
8. References 28

1. Experimental section

For all experiments and measurements, the following chemicals were used: hexagonal GeO_2 (*Thermo Scientific*, 99.999% trace metal basis), fused SiO_2 (*Sigma-Aldrich*, 99.9% trace metal basis), hydrogen (*Nippon Gases*, 99.99%), hydrogen(10%)-argon(90%) mixture (*Nippon Gases*), natural gas (Giessen city supply), oxygen (*Nippon Gases*, 99.5%) and hydrogen peroxide (*VWR*, 33 wt%).

1.1. Chemical vapour transport

All recovery processes were performed inside a quartz tube (inner/outer diameter: 14.5 mm/17 mm, cross-sectional area: 1.65 cm^2). The experiments were carried out in a three-zone furnace (*Carbolite* TZF 12/65/550). An amount of 314 mg (3.00 mmol) of GeO_2 was placed in the quartz tube at the centre of the third heating zone (T_3 in **Figure 2**). For the upscaling investigation, 942 mg (9.00 mmol) of GeO_2 and for the SiO_2/GeO_2 (15 wt% Ge) investigation, a well-grounded mixture of 523 mg (8.70 mmol) and 144 mg (1.38 mmol) was used and also placed in the middle of the third heating zone (T_3 in **Figure 2**).
For all three transport agents used (hydrogen, argon-hydrogen mixture or natural gas), the recovery process followed the same procedure. First, the quartz tube was evacuated for 15 min. It was then purged with argon for 15 min to ensure an oxygen-free atmosphere during the reaction. Next, the three furnace zones were heated to establish the desired temperature gradients. Once the target temperatures were reached, the argon flow was stopped, and the reducing agent was introduced at the specified flow rate. After the designated reaction time, the furnace was switched off and allowed to cool to room temperature under an argon atmosphere.

1.2. Oxidation of source material after NG-based CVT

The oxidation process to remove the carbon deposition was carried out immediately after the natural gas-based CVT. To completely remove residual reducing agent, the tube was first purged with argon. Subsequently, all heating zones were heated to 925 °C and an oxygen flow of 0.8 $NL \cdot h^{-1} \cdot cm^{-2}$ was introduced. After a reaction time of 2 h, the atmosphere was switched to argon, and the furnace was cooled to room temperature.

1.3. Determination of CVT parameters

To determine the Ge recovery yield and transport rate of the CVT recovery method, MP-AES was employed. The sink material was detached from the glass wall by dissolving it in a water:hydrogen peroxide (80:1) mixture, first heated to 45 °C for 1 h, then to 100 °C under reflux for 20 h. The resulting solution was filtered through a syringe filter (LLG-syringe filters, SPHEROS, PTFE, 0.22 µm, Ø 25 mm) to remove potential glass particles. The Ge concentration was measured using a 4210 MP-AES with MP Expert Software (ver. 1.6.0.9255) from Agilent Technologies and the external calibration method. A commercial standard solution for Ge (Merck, Certipur®, (NH4)2GeF6 in H2O, 1000 $mg \cdot L^{-1}$), was diluted to prepare calibration standards of 1 $mg \cdot L^{-1}$, 5 $mg \cdot L^{-1}$, 10 $mg \cdot L^{-1}$, 25 $mg \cdot L^{-1}$, 50 $mg \cdot L^{-1}$, 75 $mg \cdot L^{-1}$, 100 $mg \cdot L^{-1}$ and 125 $mg \cdot L^{-1}$ for external calibration (see Fig. S5). The emission intensity was recorded at the characteristic Ge atomic emission wavelength of 275.459 nm. For each sample, the emission intensity was measured three times, and the average intensity was used to calculate the final Ge concentration. The calculated error range for the recovery yields and transport rates is in all cases <1% and <2 $mg \cdot h^{-1}$, respectively.

1.4. Powder X-Ray diffraction

The samples were analysed using an Empyrean with Data Collector (ver. 7.2a) software from Malvern PANalytical. An Empyrean Cu LFF X-ray tube (λ(Cu-Kα) = 154.06 pm) operating at 40 kV and 40 mA was used as an X-ray source. The X-rays were focused using Soller slits (0.04 rad), a beam mask (10 mm), a fixed divergence slit (0.5°) and a fixed anti-scatter slit (1°). After focusing, the X-rays were diffracted by the sample, which was positioned flat on top of a silicon wafer. Subsequently, the X-rays underwent a fixed anti-scatter slit (0.5°), Soller slits (0.04 rad) and a Nickel Beta-filter towards a PIXcel3D detector. A Bragg-Brentano geometry was carried out between 10° and 60° in 2θ at room temperature and ambient atmosphere. Ultimately, the diffractograms were background-corrected using HighScore Plus (ver. 3.0e (3.0.5), Malvern PANalytical) and plotted using OriginPro 2024b (ver. 10.1.5.132, OriginLab).

1.5. Energy dispersive X-ray analysis

To prevent sample charging, all specimens were sputter-coated in advance with 5 nm platinum. Elemental compositions were determined using a GeminiSEM 560 from Carl Zeiss equipped with an Oxford Instruments Ultim®Max detector and operated with the software AztecLive (ver. 6.1) from Oxford Instruments. Focusing was carried out with the software SMART SEM (ver. 6.08) from Carl Zeiss. Samples were mounted on an aluminium holder using sticky carbon pads. Measurements were performed with a 60 µm aperture and 6 kV acceleration voltage at a working distance of 8,5 mm, employing a Schottky field emitter as the electron source. The acquired spectra were background-corrected and plotted using OriginPro 2024b (ver. 10.1.5.132, OriginLab).

1.6. Simultaneous thermal analysis

The oxidation temperature of Ge and the water content of the used GeO_2 (hex.) were determined via simultaneous thermal analysis (differential thermal analysis + thermogravimetry) using an STA 409 PC Luxx® coupled to a QMS 403 Aëolos Quadro mass spectrometer from *NETZSCH*. The quasi-multiple ion detection method was used to record mass spectroscopic signals. The measurements were carried out in Al_2O_3-crucibles over a temperature range of 20 °C to 1000 °C with a heating rate of 5 K · min^{-1}, under a constant synthetic air flow of 30 mL · min^{-1} and a protective argon flow of 20 mL · min^{-1}. For each run, 15-20 mg of the respective sample was used. Data was processed with Proteus Thermal Analysis software (ver. 6.1.0, *NETZSCH)* and visualised using OriginPro 2024b (ver. 10.1.5.132, *OriginLab*).

2. Chemical vapour transport (CVT)

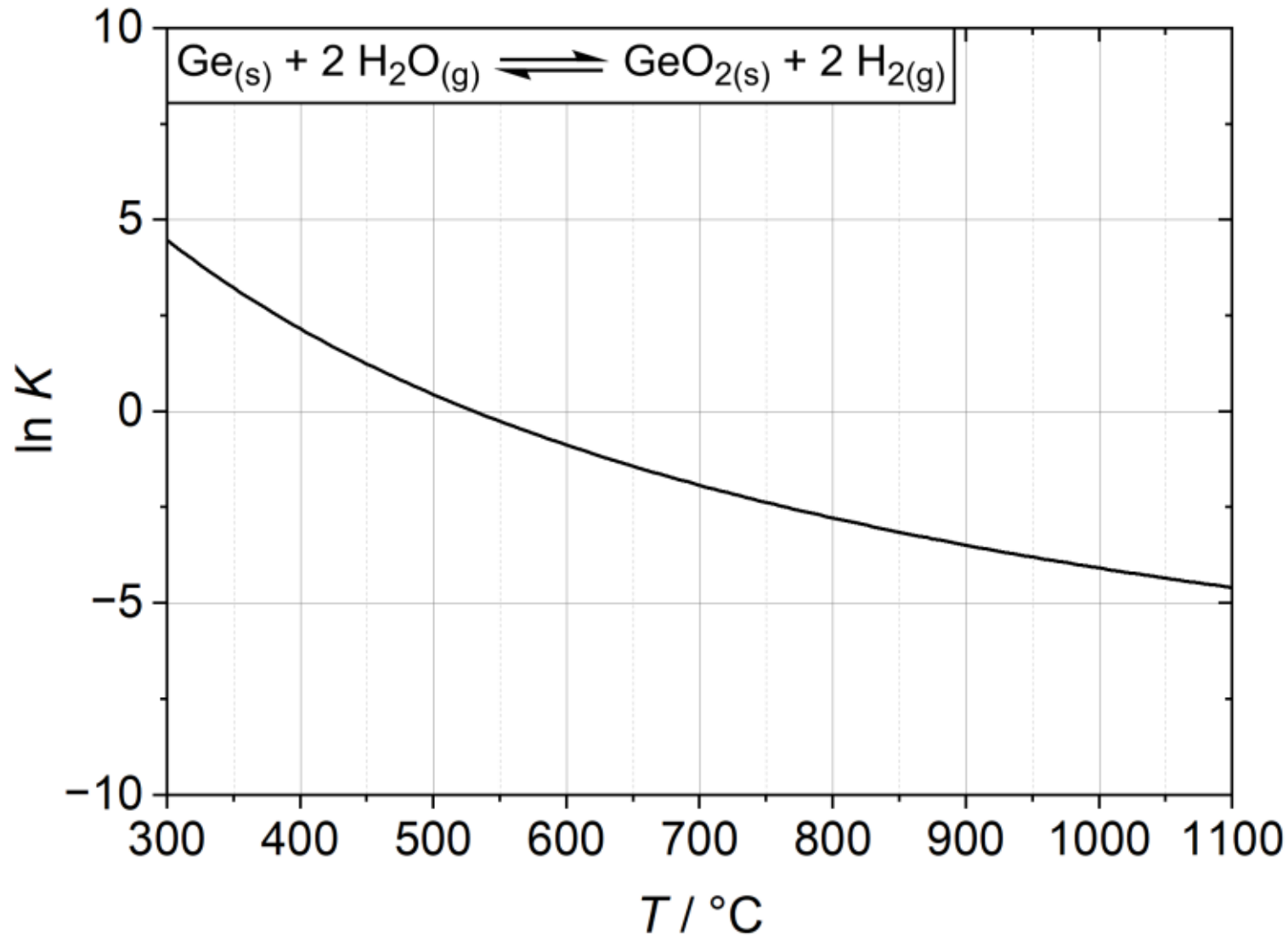

Figure S1 Logarithmic equilibrium constant of the oxidation of Ge to GeO_2 with water in a temperature range of 300 °C to 1100 °C is shown. The logarithmic equilibrium was calculated using the van't-Hoff equation[49] based on the enthalpies and entropies given in the literature[50-52].

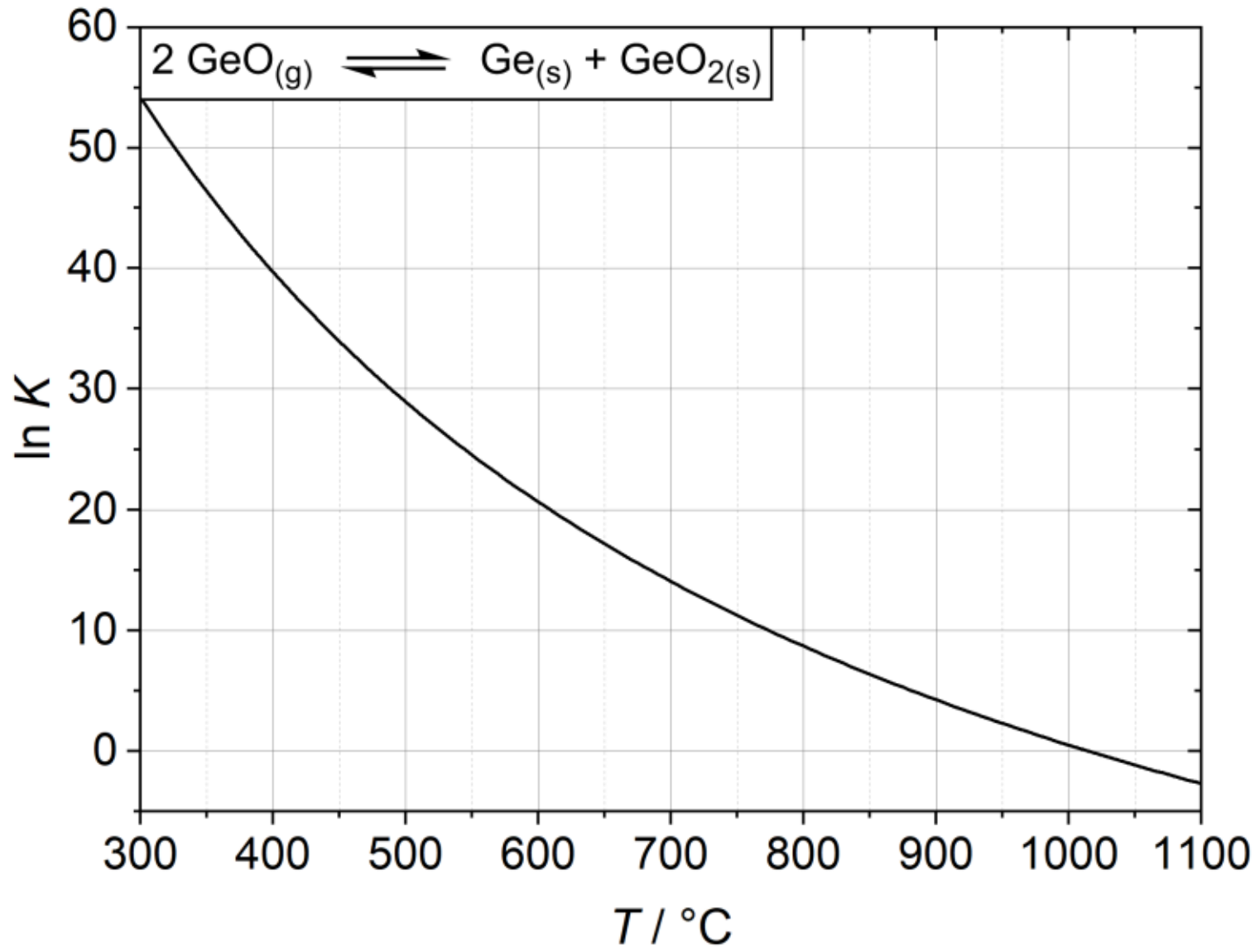

Figure S2 Logarithmic equilibrium constant of the disproportionation of GeO to GeO_2 and Ge in a temperature range of 300 °C to 1100 °C is shown. The logarithmic equilibrium was calculated using the van't-Hoff equation[49] based on the enthalpies and entropies given in the literature[50-52].

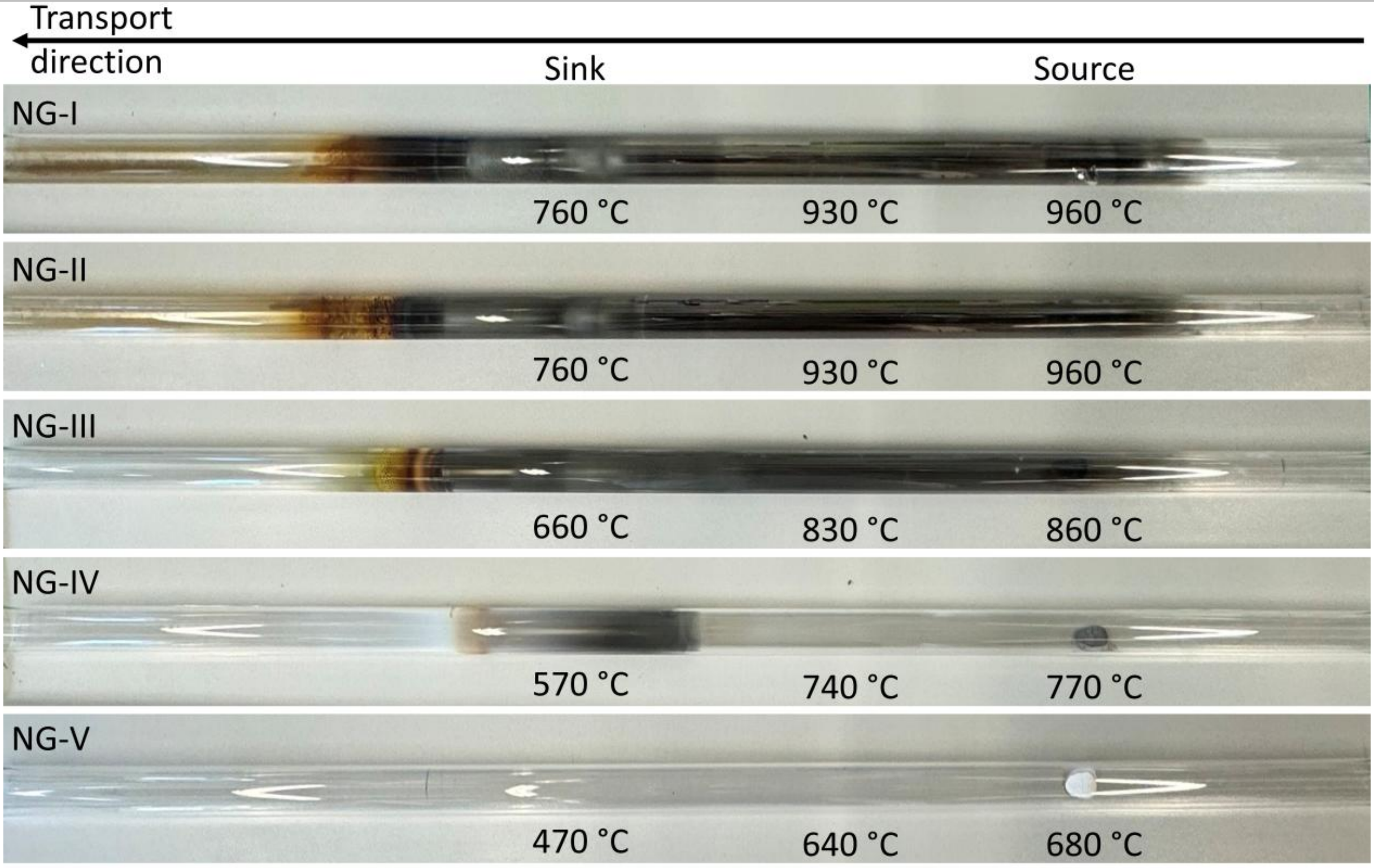

Figure S3 Overview of all NG reaction tubes. The sink and source are marked at the top, as well as the transport direction. In addition, the specific temperatures of the three heating zones are given for each reaction tube.

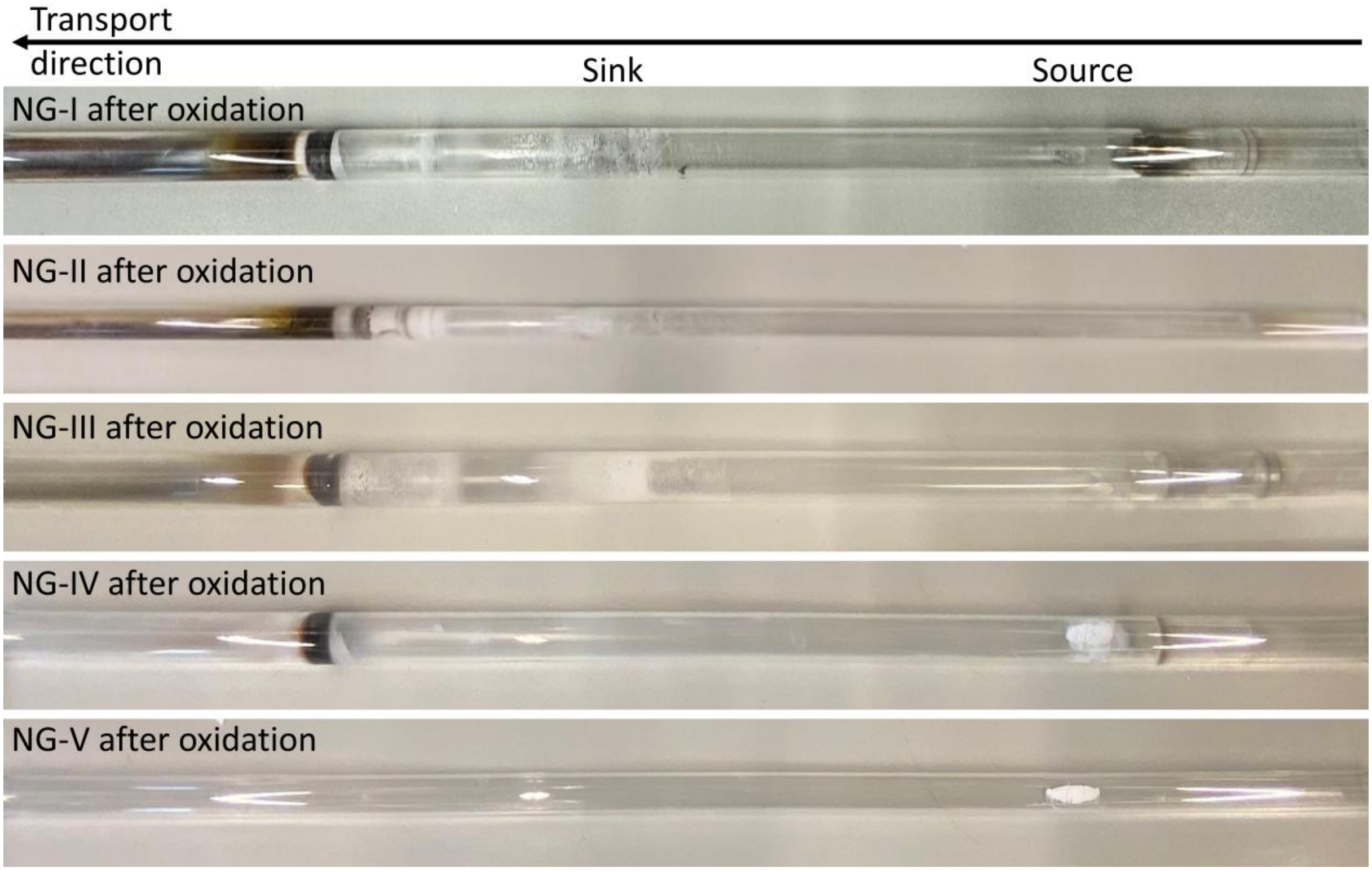

Figure S4 Overview of all NG reaction tubes after oxidation with oxygen at 925 °C. The sink and source are marked at the top, as well as the transport direction.

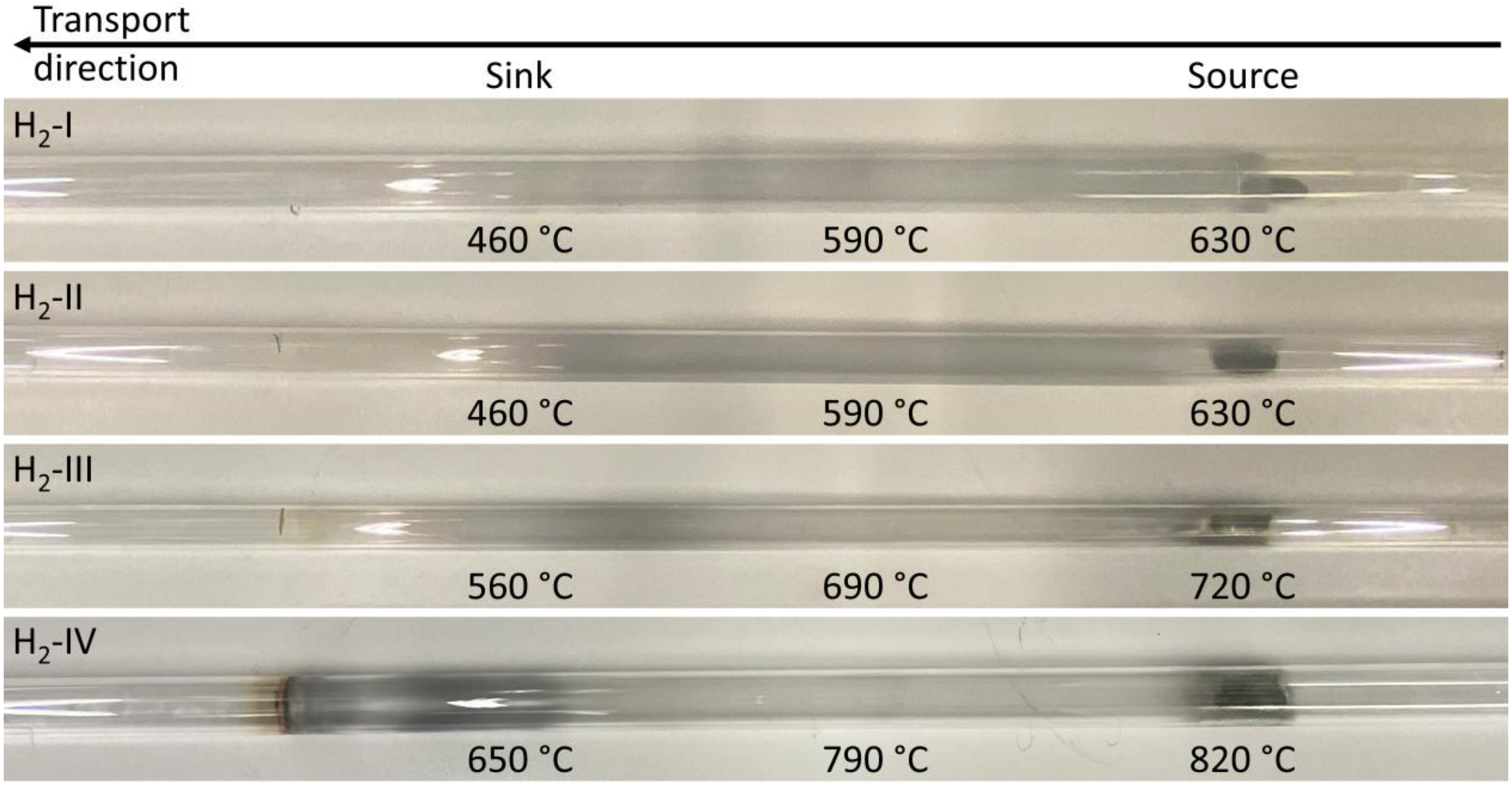

Figure S5 Overview of all H_2 reaction tubes. The sink and source are marked at the top, as well as the transport direction. In addition, the specific temperatures of the three heating zones are given for each reaction tube.

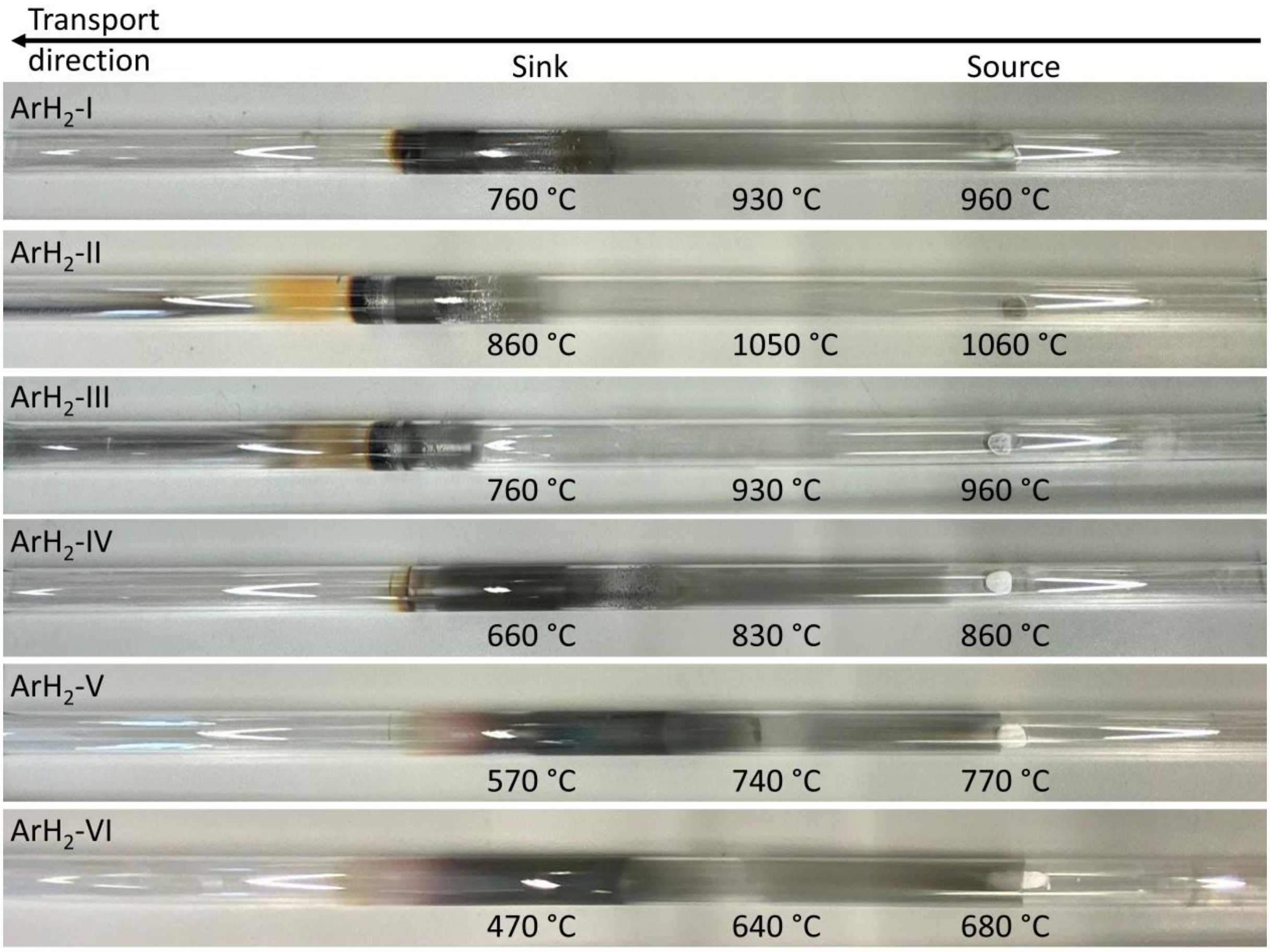

Figure S6 Overview of all ArH_2 reaction tubes. The sink and source are marked at the top, as well as the transport direction. In addition, the specific temperatures of the three heating zones are given for each reaction tube.

Table S1. Reaction time investigations of the CVT performed with ArH_2 as a reducing agent. In all cases, ArH_2 was passed through water. For each experiment, temperature gradient, reaction time and ArH_2 flow rate are listed. Additionally, the recovery yield are given.

T_1 [°C]	T_2 [°C]	T_3 [°C]	t [min]	$Q(ArH_2)$ [NL · h^{-1} · cm^{-2}]	Water	Recovery yield (Ge) [%]
760	930	960	30	2.2	Yes	86
760	930	960	20	2.2	Yes	79
760	930	960	10	2.2	Yes	48

Table S2. Flow rate investigations of the CVT performed with ArH_2 as a reducing agent. In all cases, ArH_2 was passed through water. For each experiment, temperature gradient, reaction time and ArH_2 flow rate are listed. Additionally, the recovery yield and the transport rate are given.

T_1 [°C]	T_2 [°C]	T_3 [°C]	t [min]	$Q(ArH_2)$ [NL · h^{-1} · cm^{-2}]	Water	Recovery yield (Ge) [%]	Transport rate (Ge) [mg · h^{-1}]
760	930	960	10	0.8	Yes	30	384
760	930	960	10	2.2	Yes	48	617
760	930	960	10	4.4	Yes	72	922
760	930	960	10	7.6	Yes	73	924

3. Microwave plasma atomic emission spectroscopy (MP-AES)

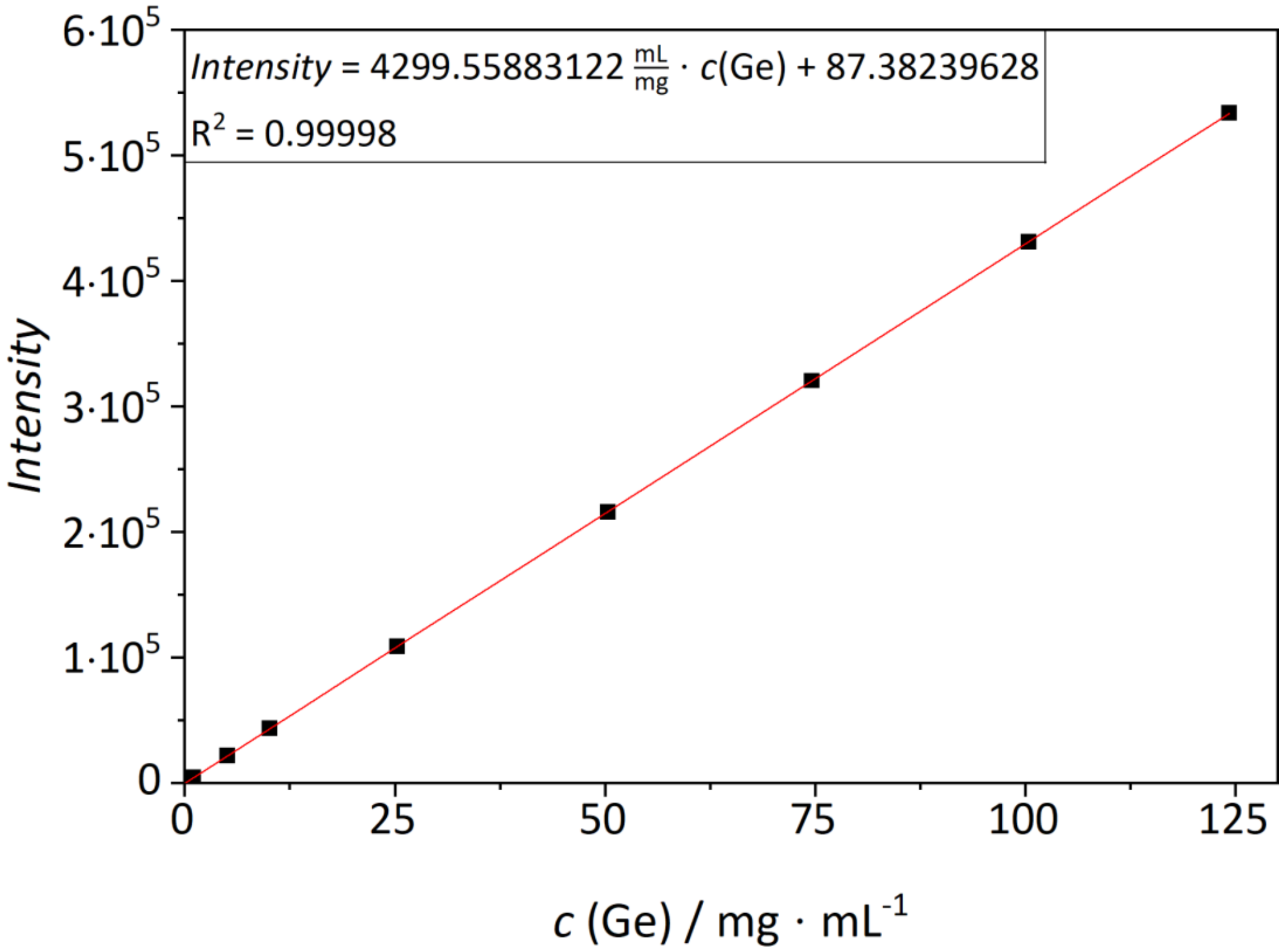

Figure S7 Exemplary external calibration of the MP-AES measurements performed with Ge-standard solution for the Ge emission line at 275.459 nm within a c(Ge) range of 0 to 125 mg · L^{-1}. The fitting parameters of the red linear fit are listed at the top left corner, as well as the correlation factor R^2.

4. Simultaneous thermal analysis (STA)

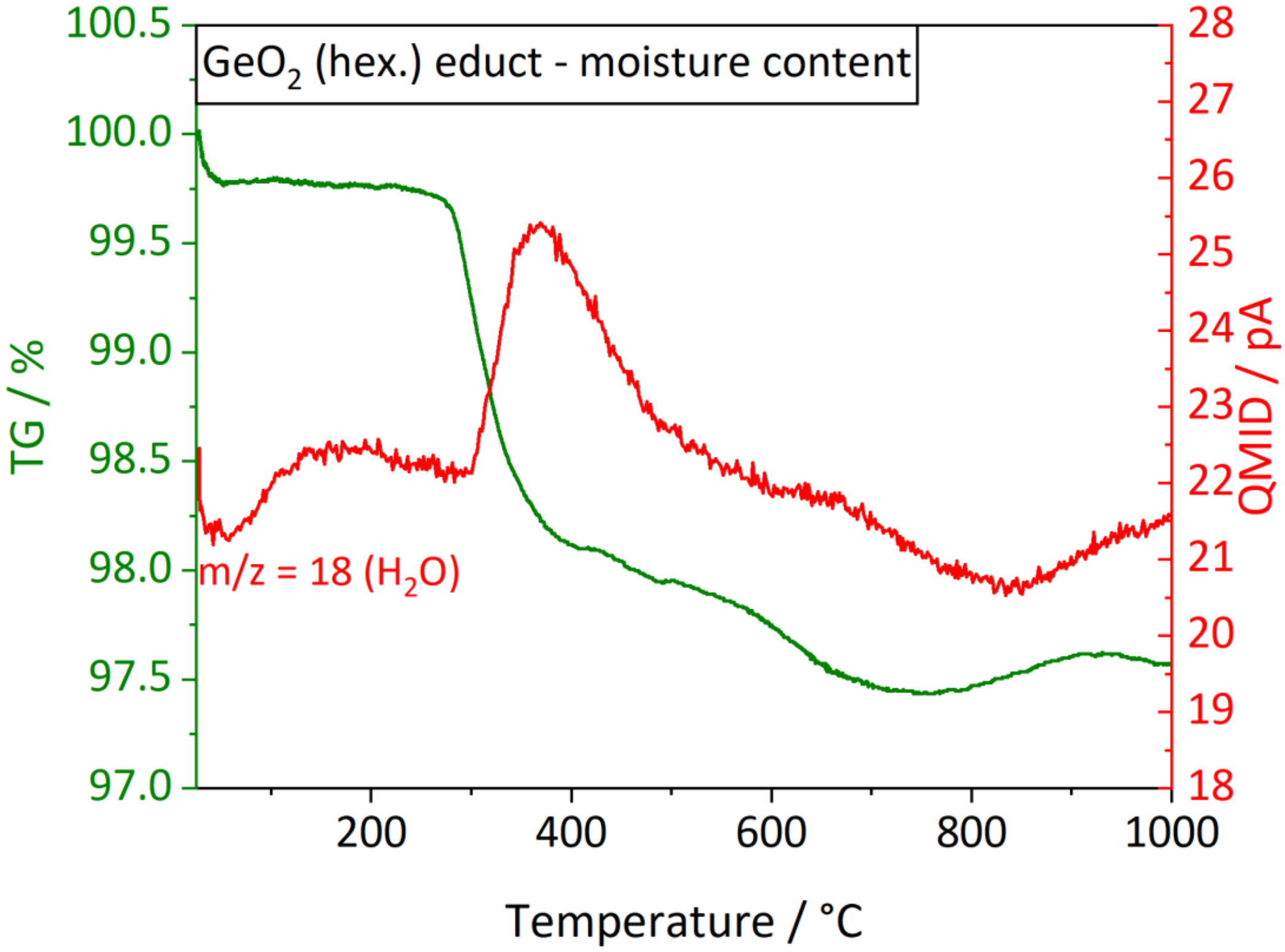

Figure S8 Determination of moisture content of the used GeO_2 (hex.) educt via STA combining thermogravimetry (TG, green curve) and mass spectrometry (QMID, red curve, m/z = 18 (H_2O)).

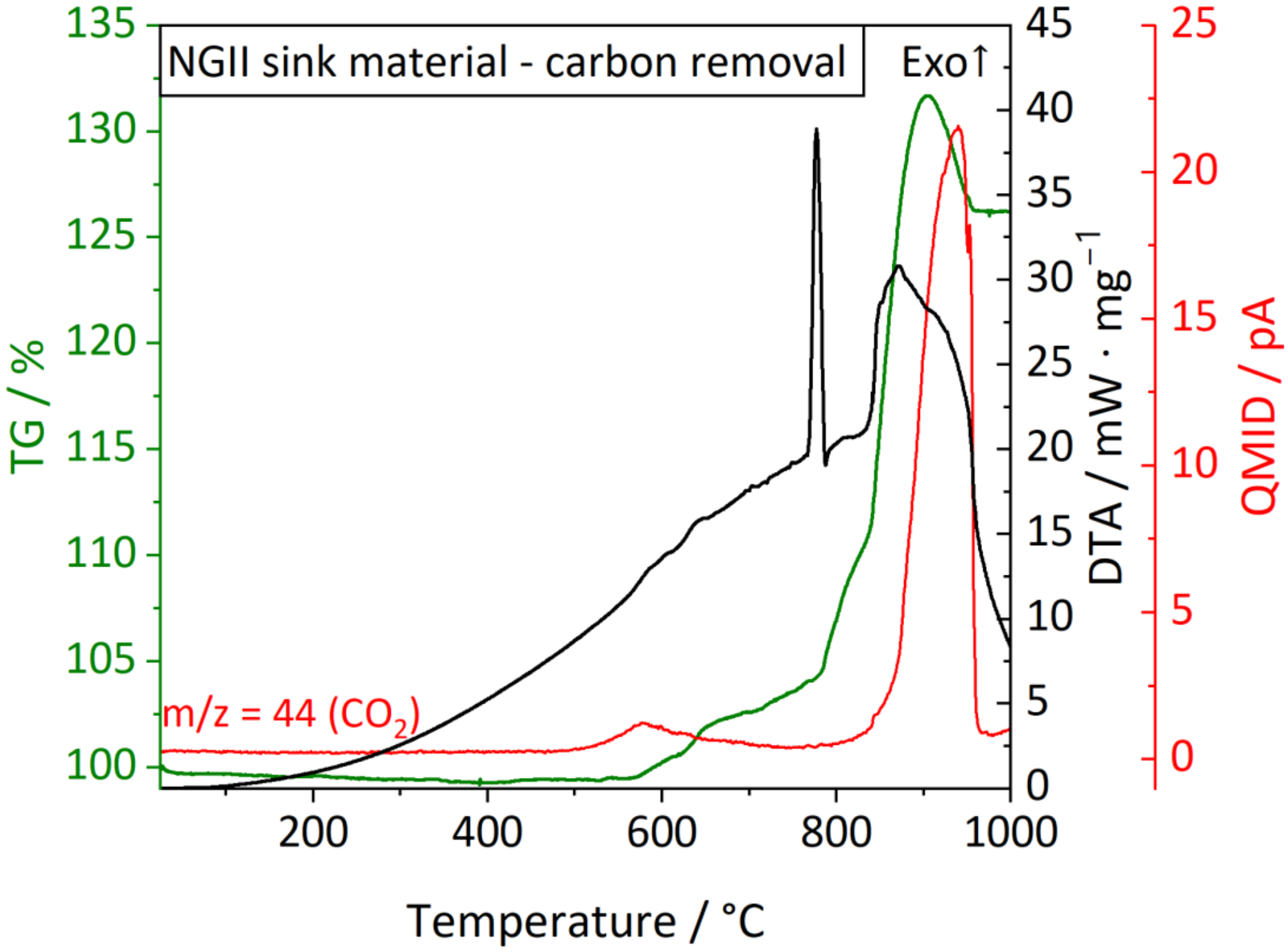

Figure S9 Investigation of the carbon removal of NG II sink material via STA in synthetic air atmosphere combining differential thermal analysis (DTA, black curve), thermogravimetry (TG, green curve) and mass spectrometry (QMID, red curve, m/z = 44 (CO_2)).

5. Powder X-ray diffractometry (PXRD)

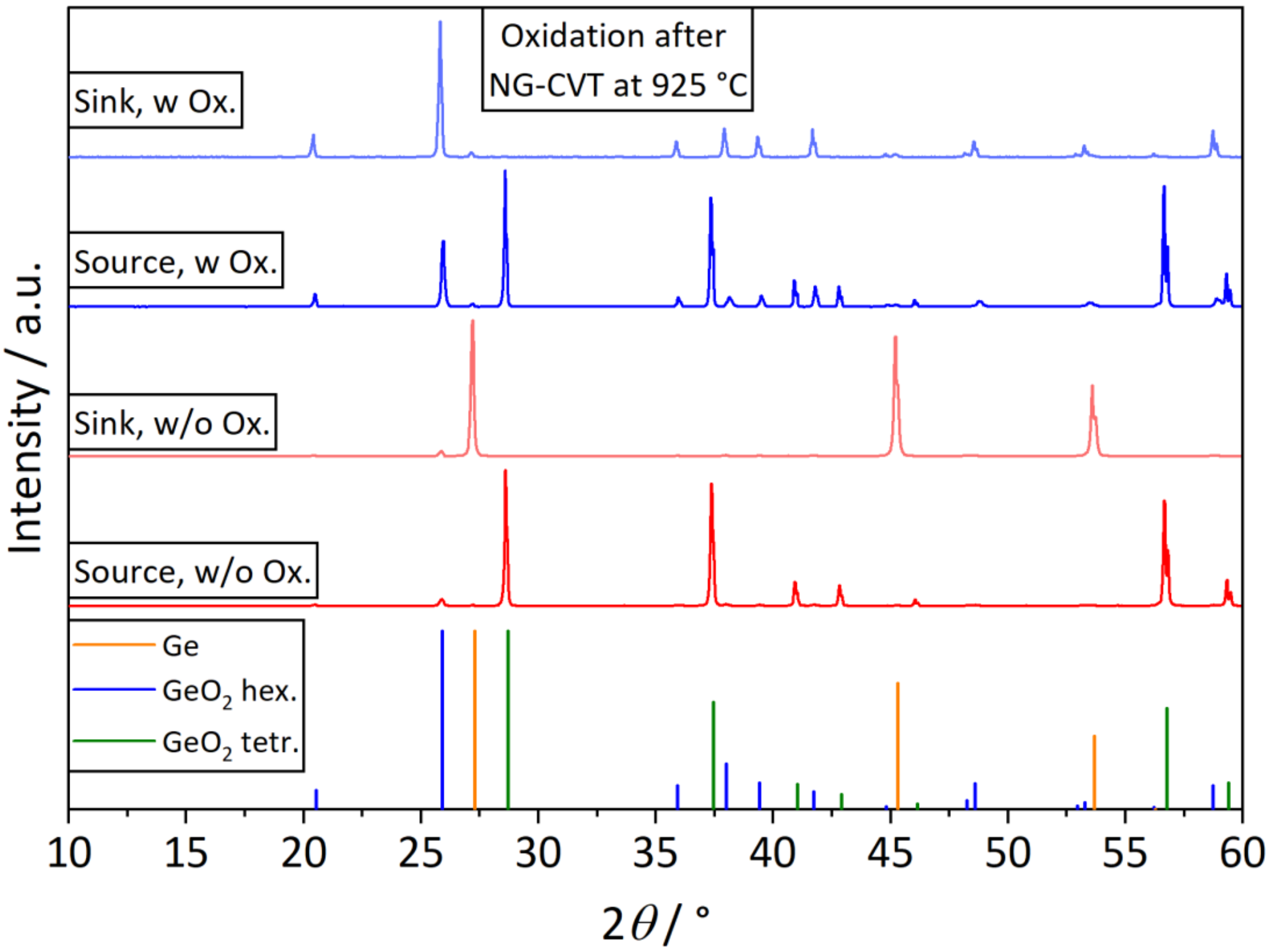

Figure S10 Comparison of PXRD data of source and sink with (w, blue curves) and without (w/o, red curves) O_2-oxidation at 925 °C. Additionally, references for germanium[55], hexagonal germanium dioxide[56] and tetragonal germanium dioxide[57] are shown.

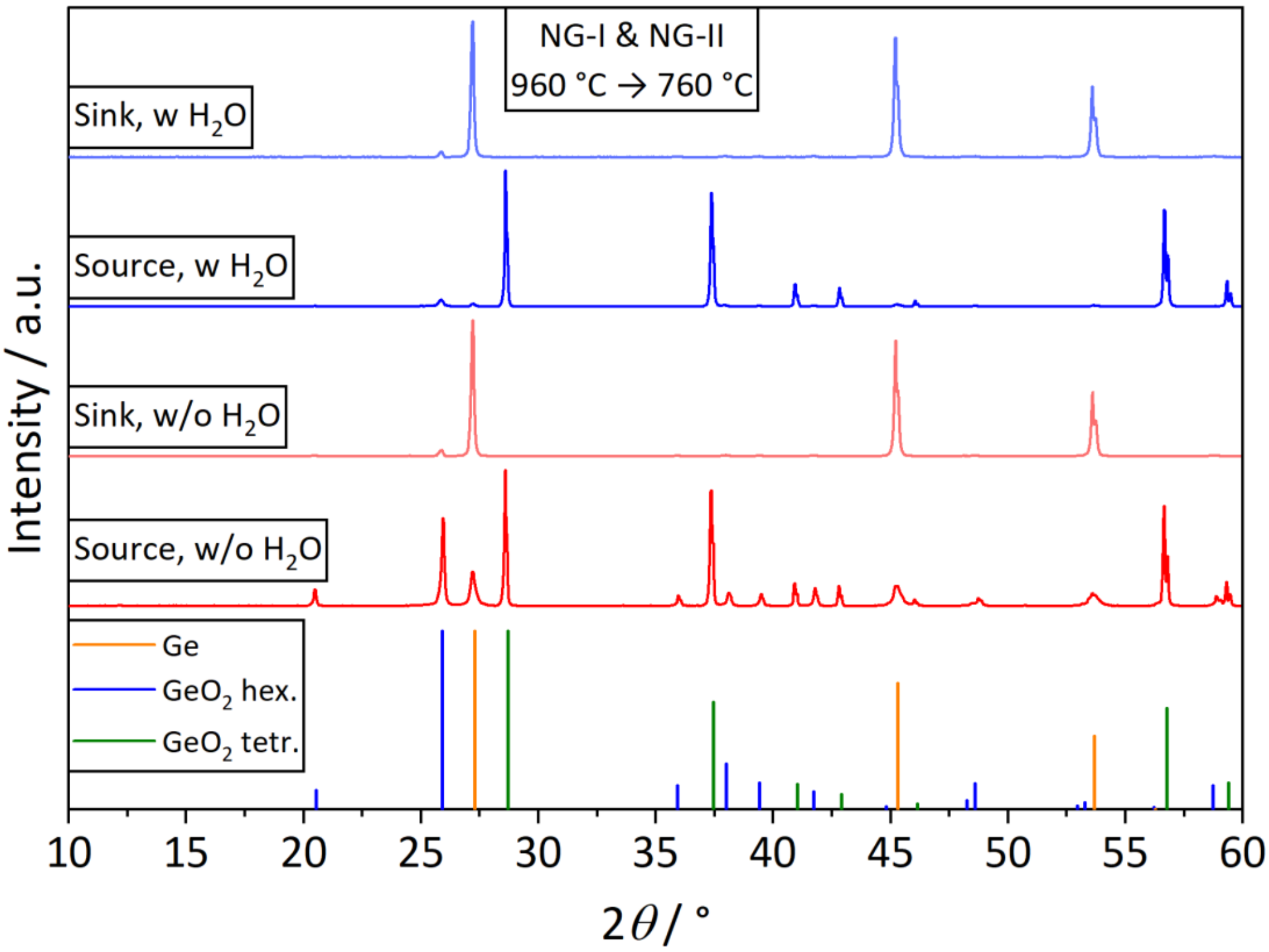

Figure S11 Comparison of PXRD data of source and sink of the NG-CVT investigations (NG-I & NG-II) with (w, blue curves) and without (w/o, red curves) water vapour. Additionally, references for germanium[55], hexagonal germanium dioxide[56] and tetragonal germanium dioxide[57] are shown.

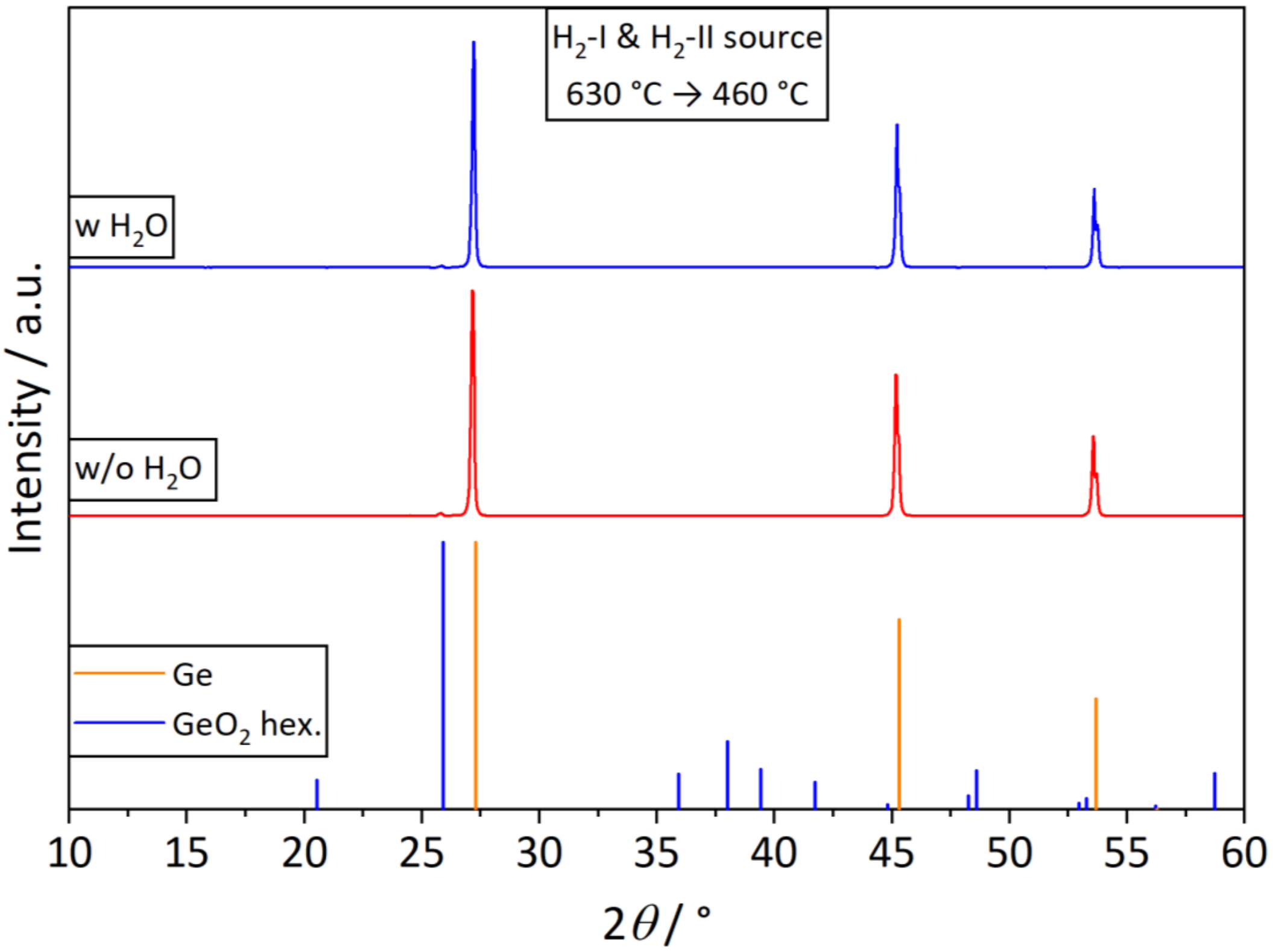

Figure S12 Comparison of PXRD data of the source of the H_2-CVT investigations (H_2-I & H_2-II) with (w, blue curve) and without (w/o, red curve) water vapour. Additionally, references for germanium[55] and hexagonal germanium dioxide[56] are shown.

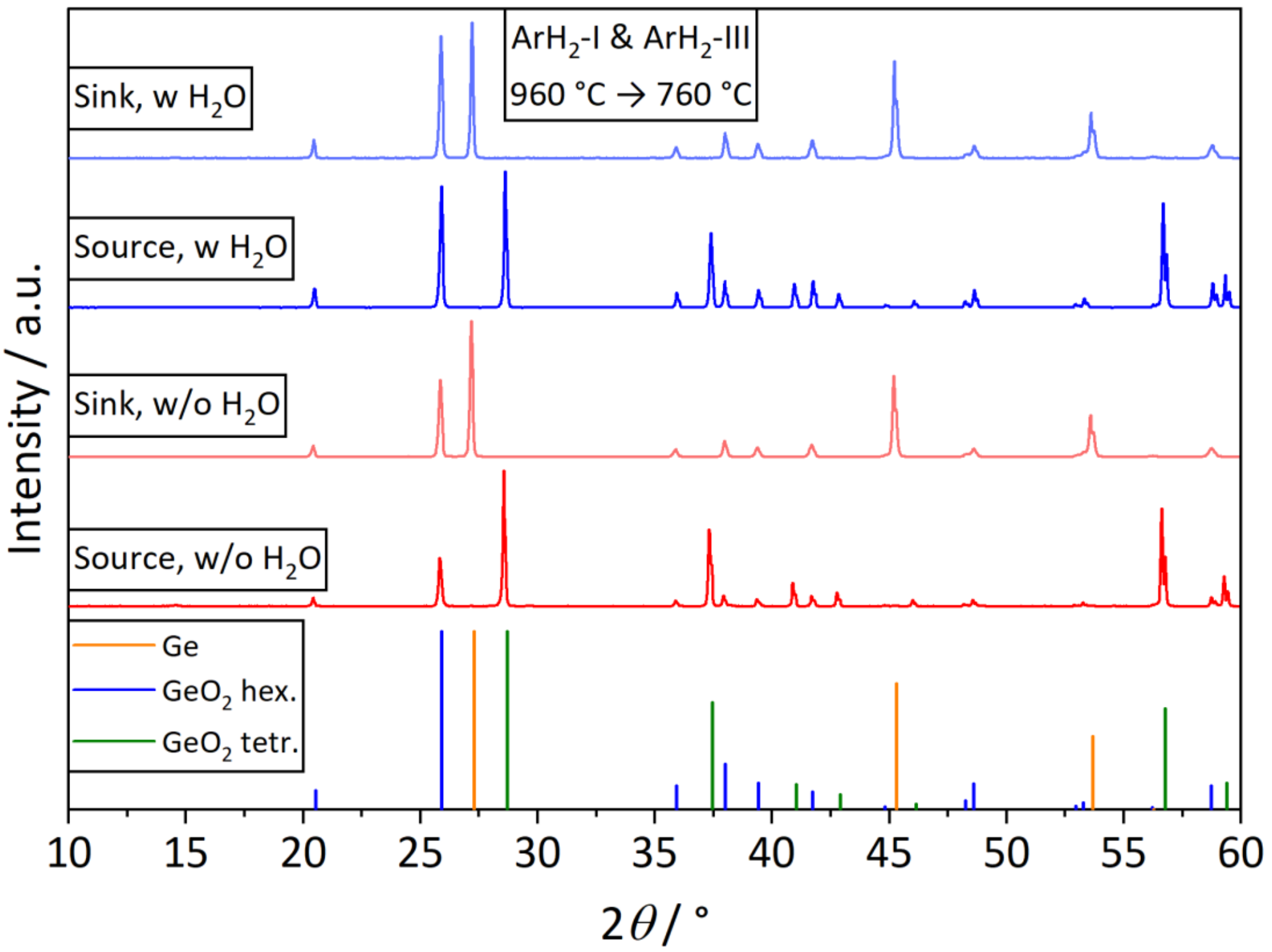

Figure S13 Comparison of PXRD data of source and sink of the ArH_2-CVT investigations (ArH_2-I & ArH_2-II) with (w, blue curves) and without (w/o, red curves) water vapour. Additionally, references for germanium[55], hexagonal germanium dioxide[56] and tetragonal germanium dioxide[57] are shown.

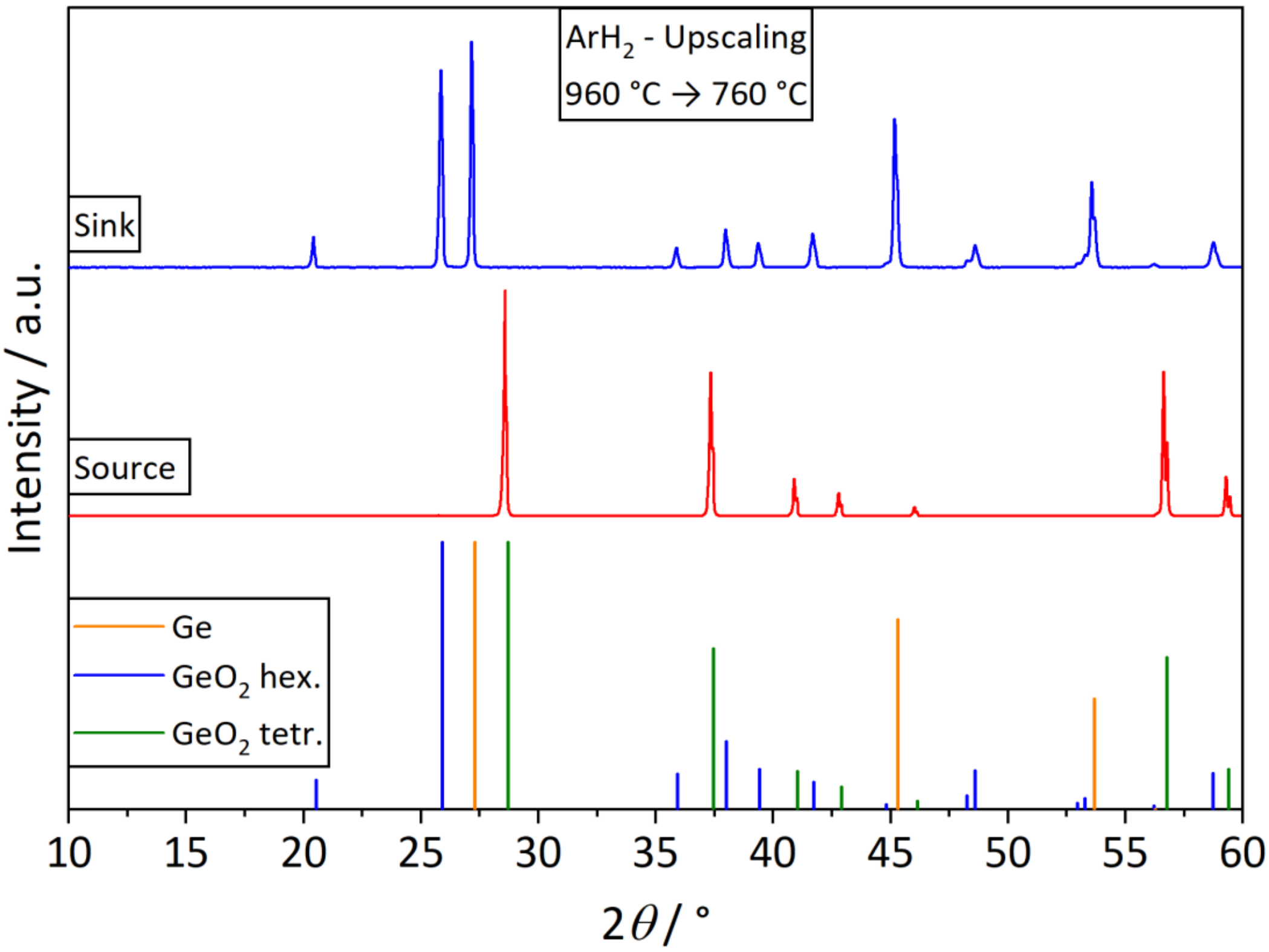

Figure S14 PXRD data of the source and sink of the ArH_2-upscaling investigation is shown. Additionally, references for germanium[55], hexagonal germanium dioxide[56] and tetragonal germanium dioxide[57] are shown.

6. Energy dispersive X-ray analysis (EDX)

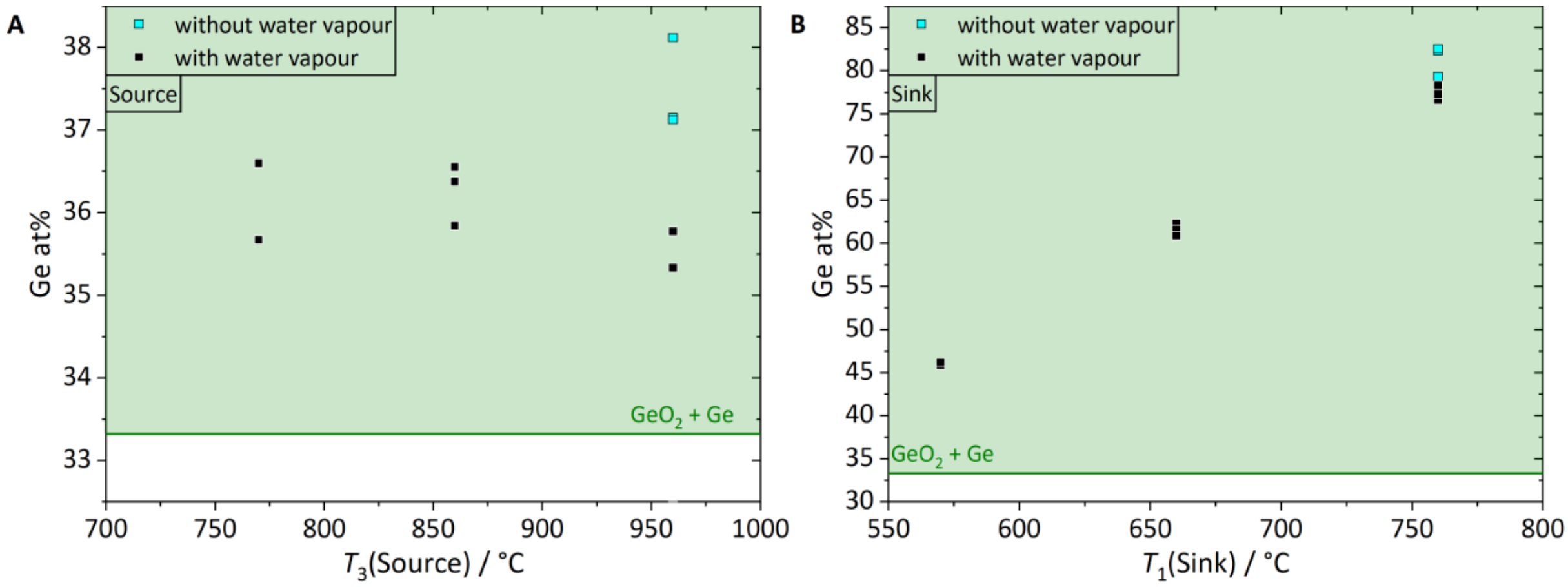

Figure S15 EDX data of source (A) and sink (B) of the CVT experiments with NG without (turquoise boxes) and with (black boxes) water vapour as transport agents are shown.

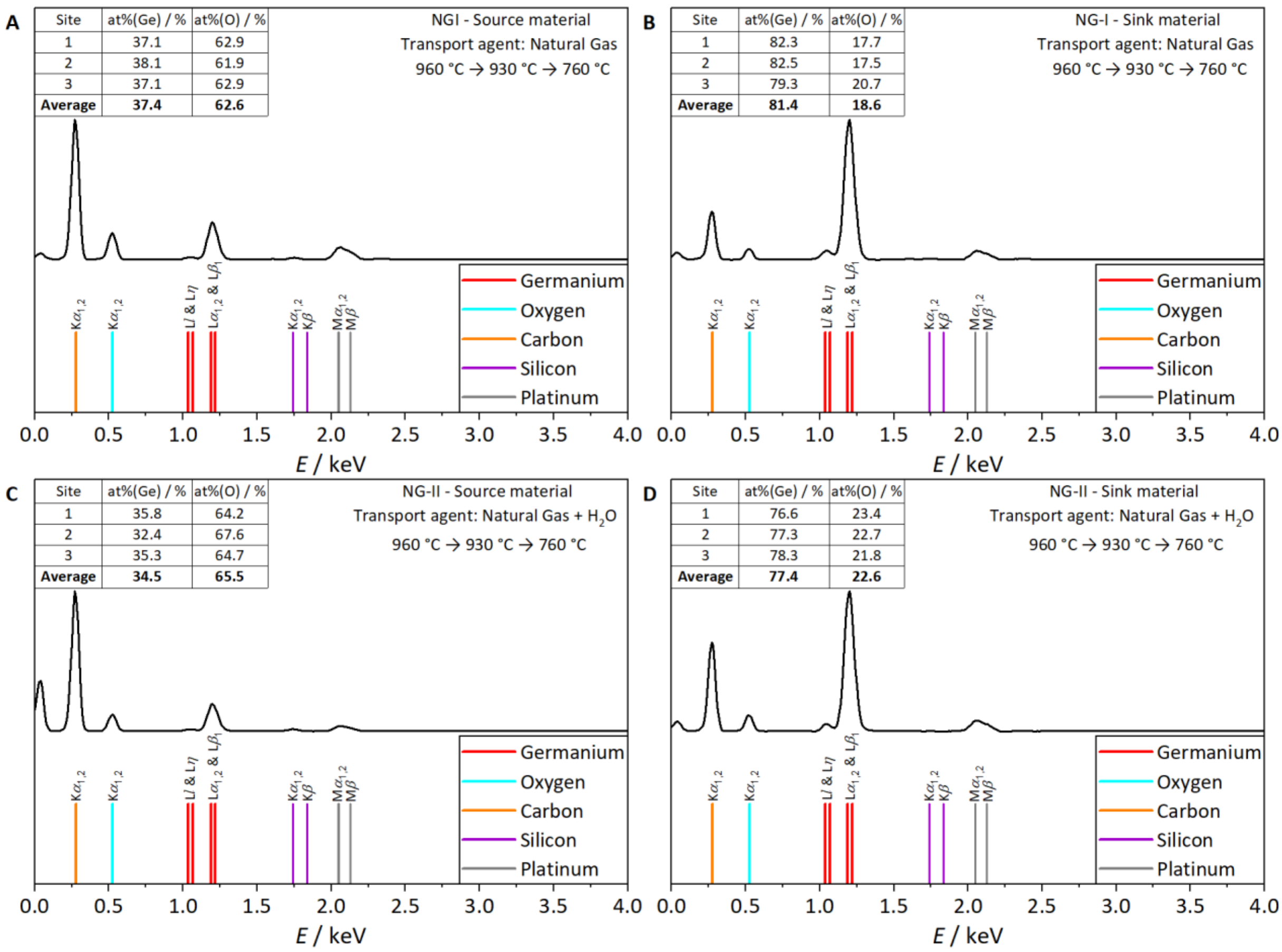

Figure S16 EDX spectra of sink and source materials without (A&B: NG-I) and with (C&D: NG-II) water vapour are shown. An average at% value of germanium and oxygen is given. It is calculated from three different measuring spots. X-ray emission lines are shown for germanium, oxygen, carbon, silicon and platinum as references.[58]

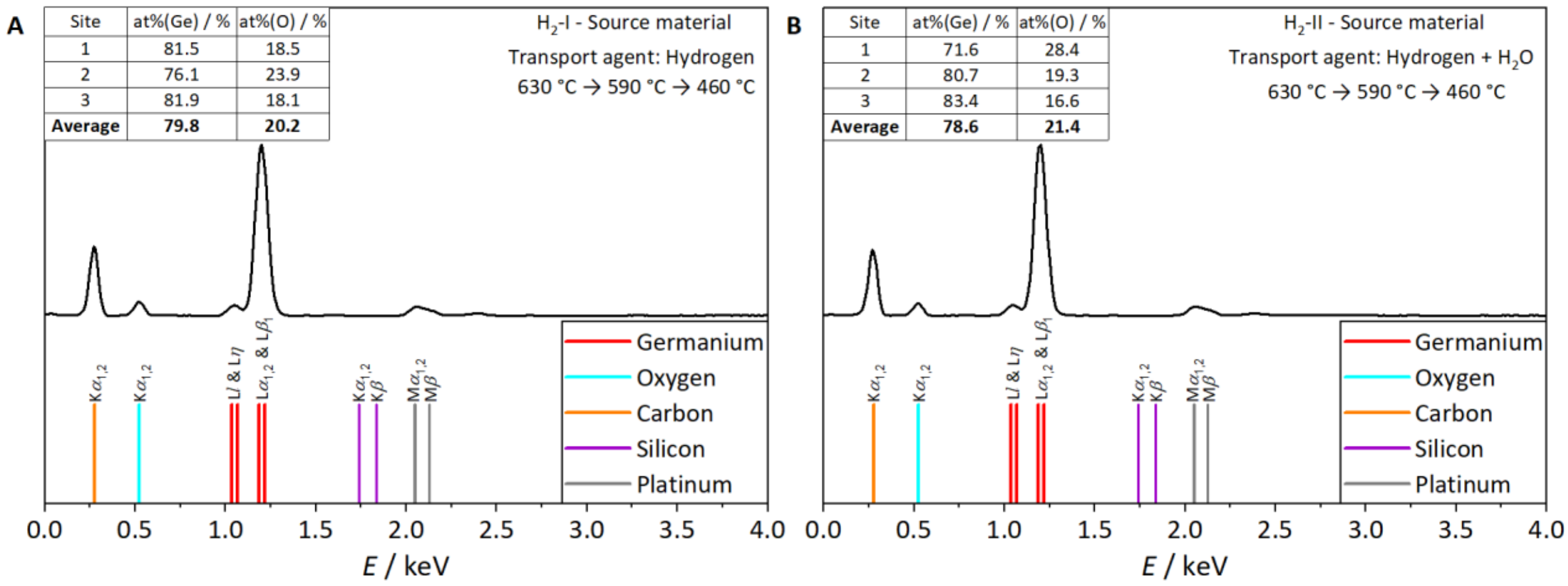

Figure S17 EDX spectra of sink and source materials without (A&B: H_2-I) and with (C&D: H_2-II) water vapour are shown. An average at% value of germanium and oxygen is given. It is calculated from three different measuring spots. X-ray emission lines are shown for germanium, oxygen, carbon, silicon and platinum as references.[58]

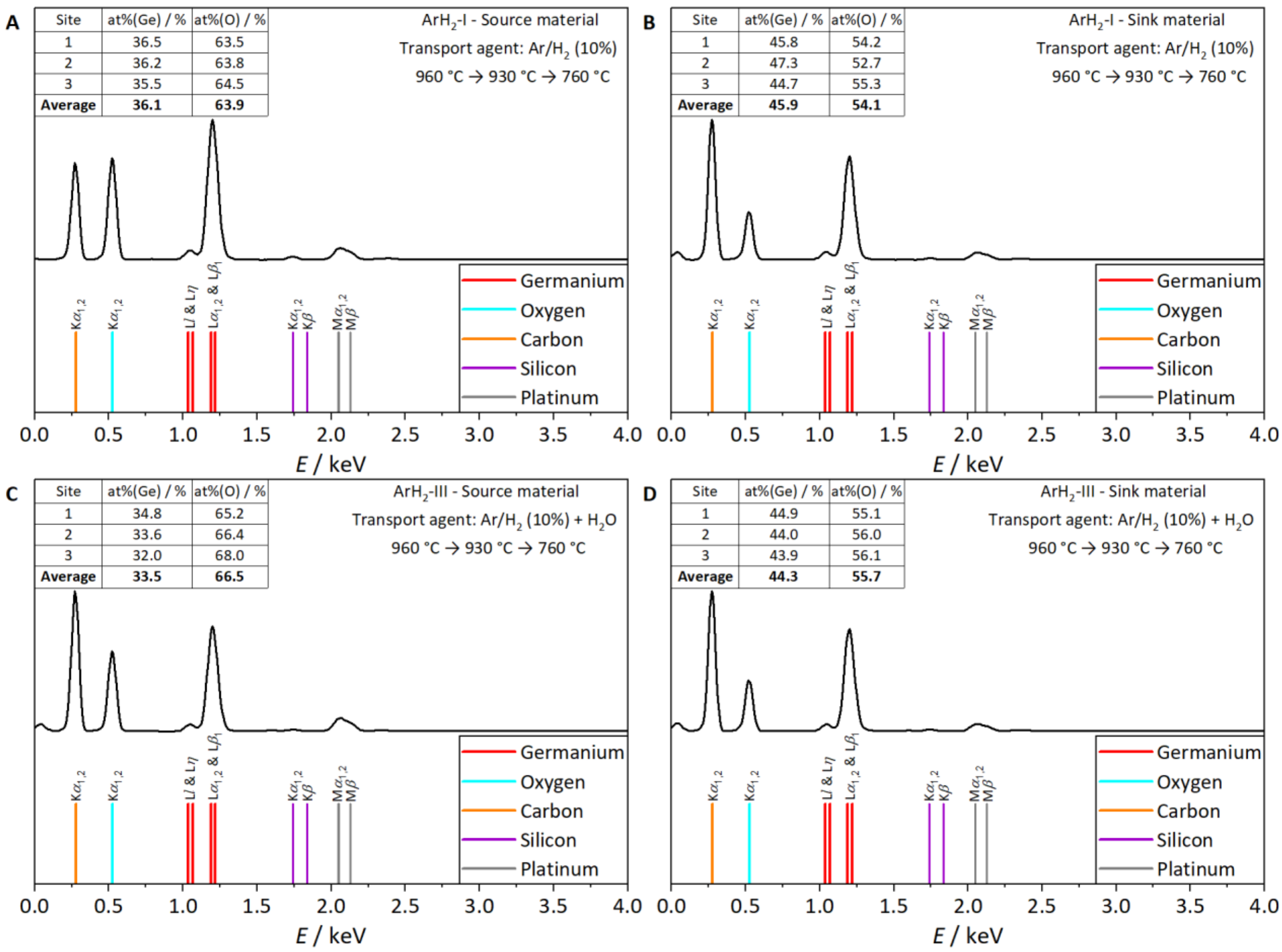

Figure S18 EDX spectra of sink and source materials without (A&B: ArH_2-I) and with (C&D: ArH_2-III) water vapour are shown. An average at% value of germanium and oxygen is given. It is calculated from three different measuring spots. X-ray emission lines are shown for germanium, oxygen, carbon, silicon and platinum as references.[58]

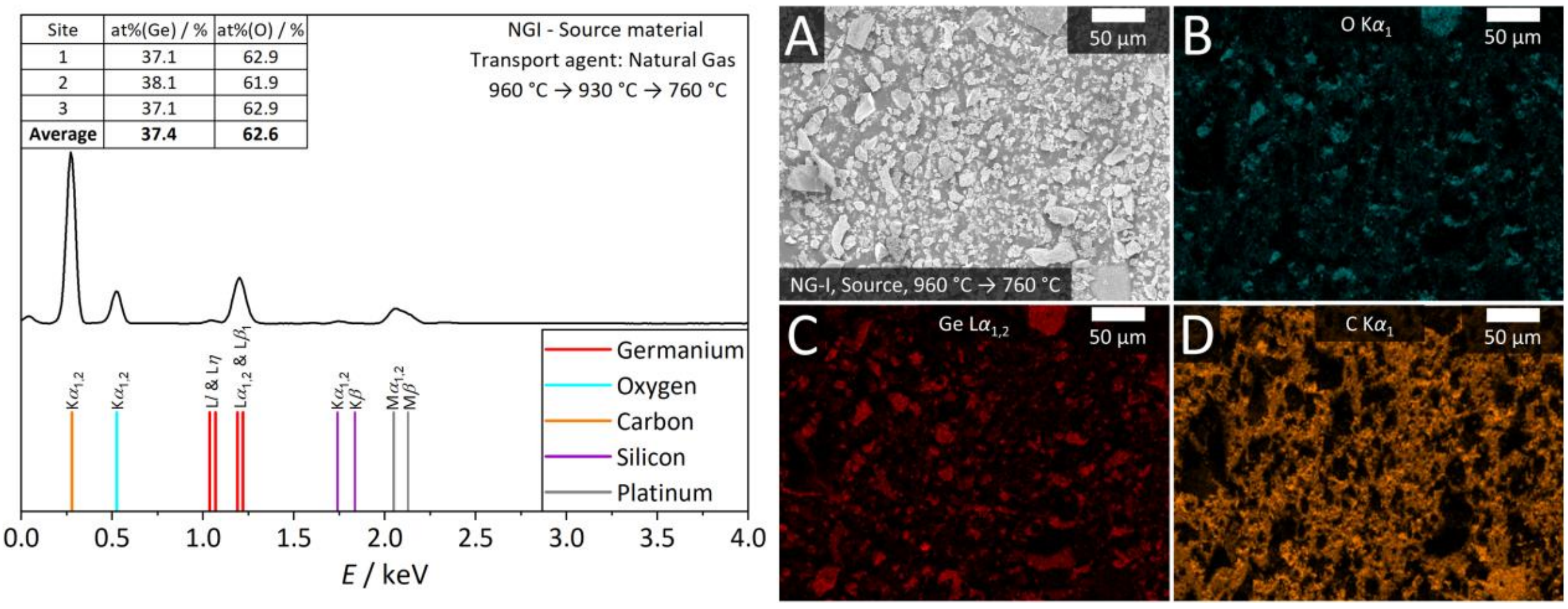

Figure S19 EDX data of the source material of investigation NG-I is shown. An average at% value of germanium and oxygen is given. It is calculated from three different measuring spots. X-ray emission lines are shown for germanium, oxygen, carbon, silicon and platinum as references.[58] Additionally, for one measuring spot of the source material, a SEM image (A), as well as the elemental mappings for germanium (C, red dots), oxygen (B, blue dots) and carbon (D, orange dots) are shown.

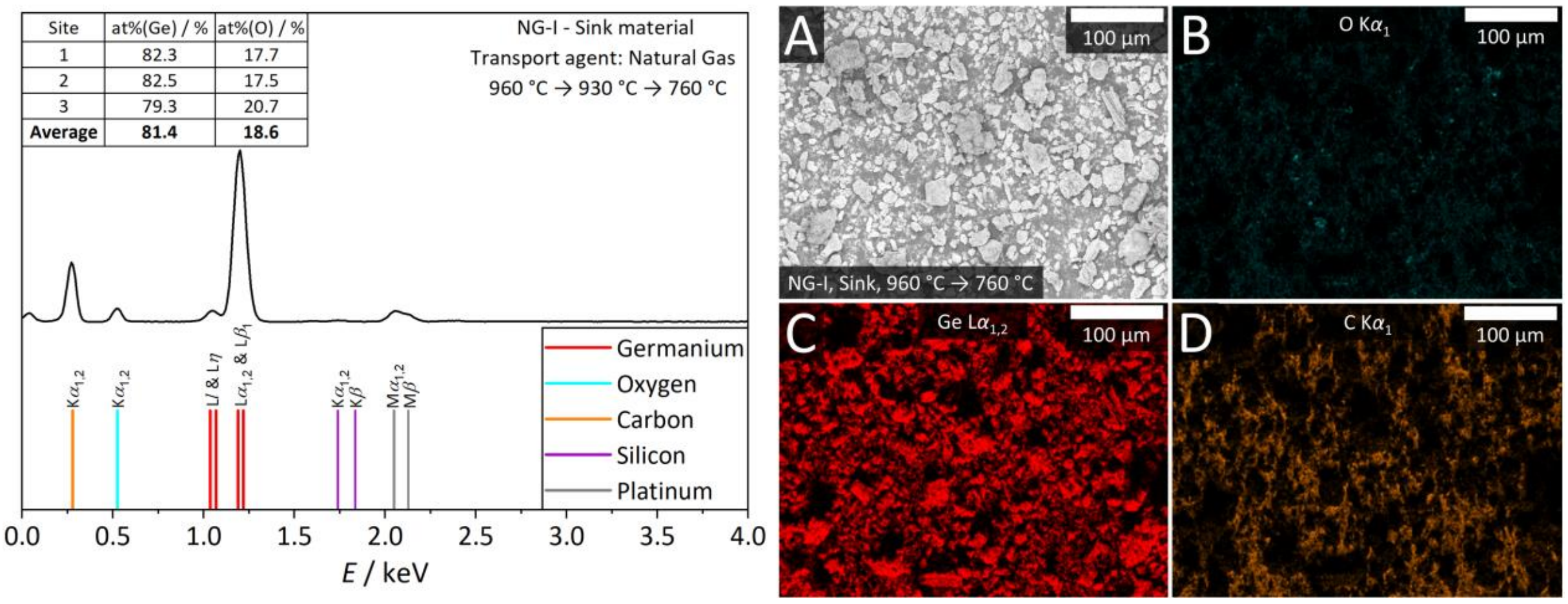

Figure S20 EDX data of the sink material of investigation NG-I is shown. An average at% value of germanium and oxygen is given. It is calculated from three different measuring spots. X-ray emission lines are shown for germanium, oxygen, carbon, silicon and platinum as references.[58] Additionally, for one measuring spot of the source material, a SEM image (A), as well as the elemental mappings for germanium (C, red dots), oxygen (B, blue dots) and carbon (D, orange dots) are shown.

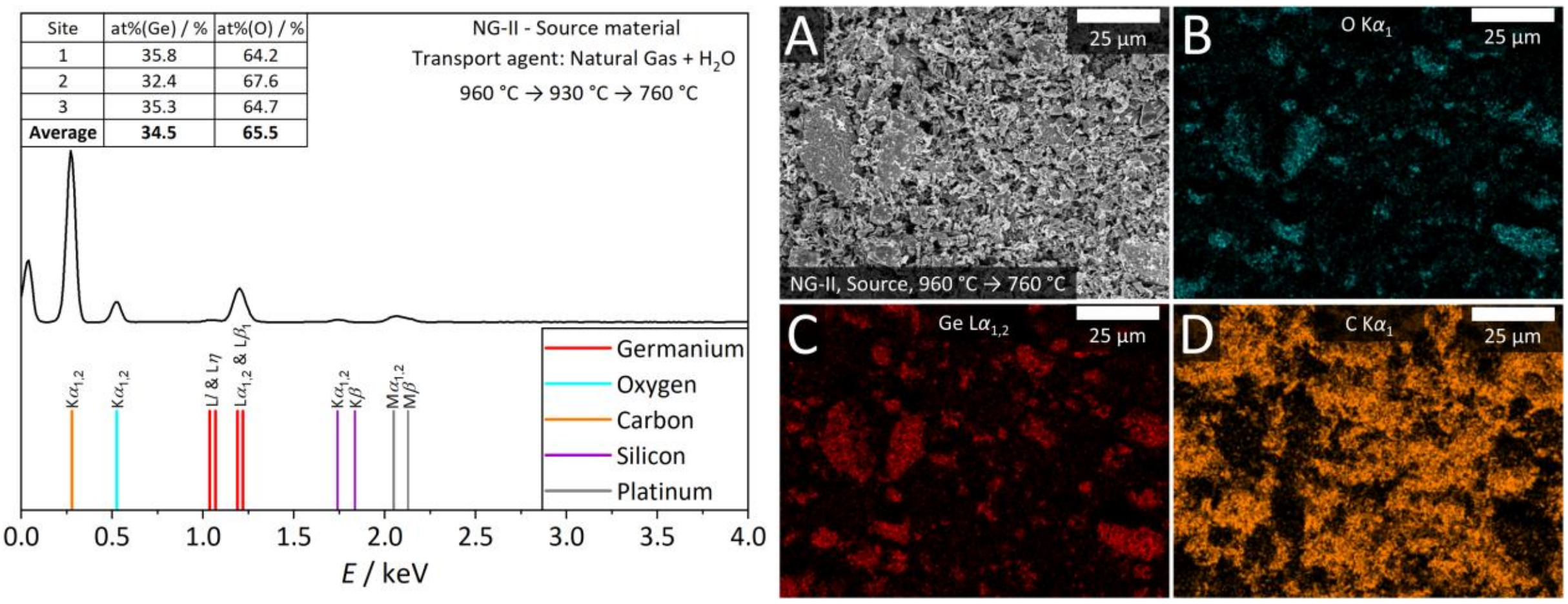

Figure S21 EDX data of the source material of investigation NG-II is shown. An average at% value of germanium and oxygen is given. It is calculated from three different measuring spots. X-ray emission lines are shown for germanium, oxygen, carbon, silicon and platinum as references.[58] Additionally, for one measuring spot of the source material, a SEM image (A), as well as the elemental mappings for germanium (C, red dots), oxygen (B, blue dots) and carbon (D, orange dots) are shown.

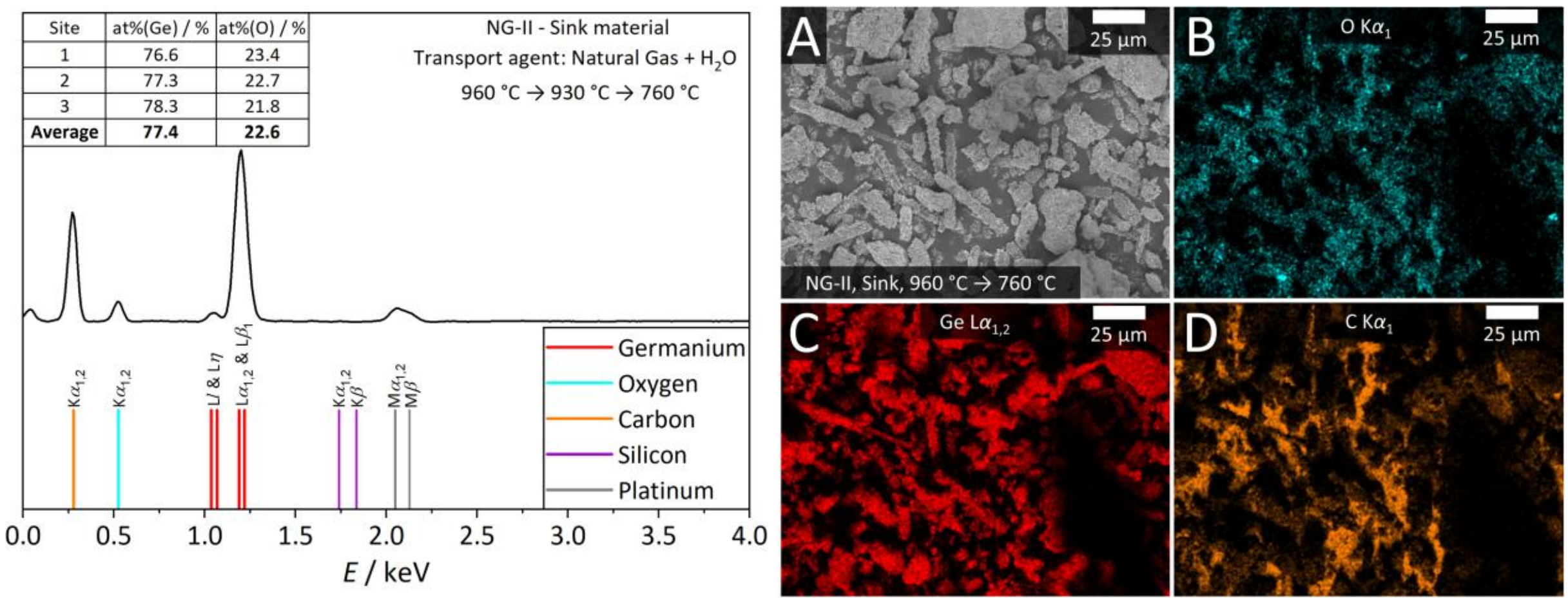

Figure S22 EDX data of the sink material of investigation NG-II is shown. An average at% value of germanium and oxygen is given. It is calculated from three different measuring spots. X-ray emission lines are shown for germanium, oxygen, carbon, silicon and platinum as references.[58] Additionally, for one measuring spot of the source material, a SEM image (A), as well as the elemental mappings for germanium (C, red dots), oxygen (B, blue dots) and carbon (D, orange dots) are shown.

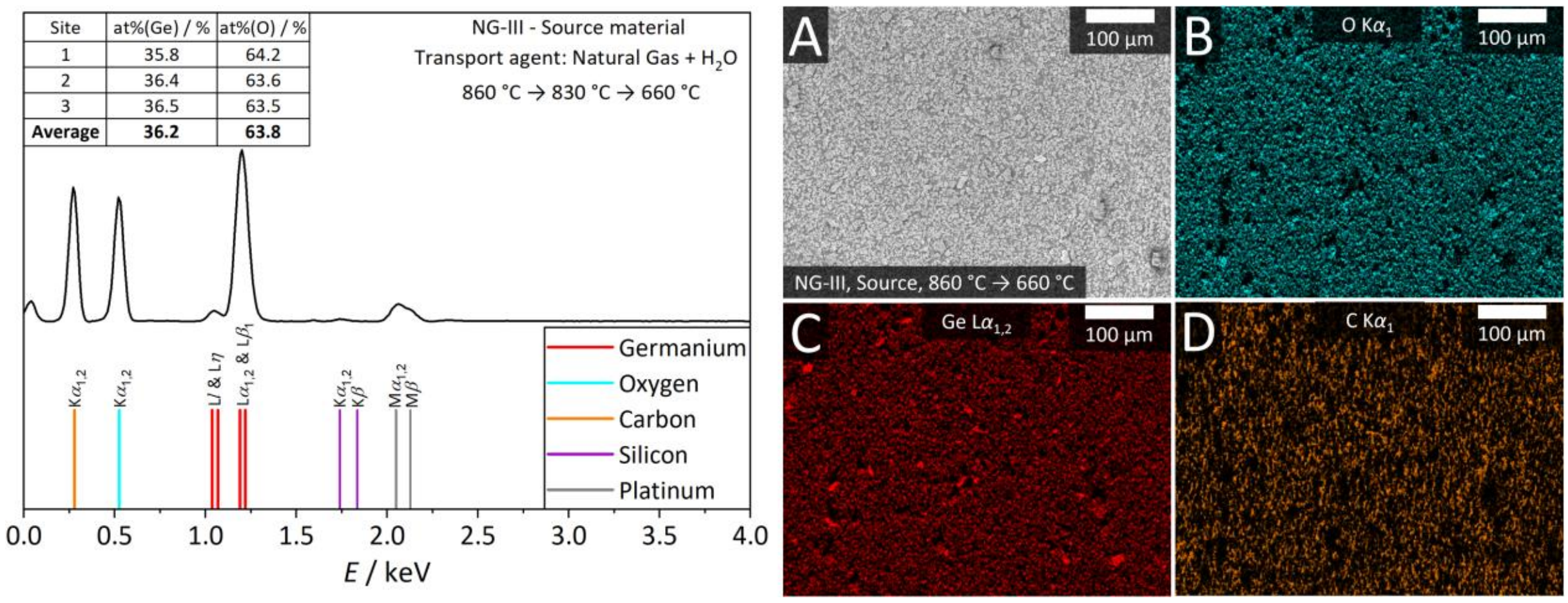

Figure S23 EDX data of the source material of investigation NG-III is shown. An average at% value of germanium and oxygen is given. It is calculated from three different measuring spots. X-ray emission lines are shown for germanium, oxygen, carbon, silicon and platinum as references.[58] Additionally, for one measuring spot of the source material, a SEM image (A), as well as the elemental mappings for germanium (C, red dots), oxygen (B, blue dots) and carbon (D, orange dots) are shown.

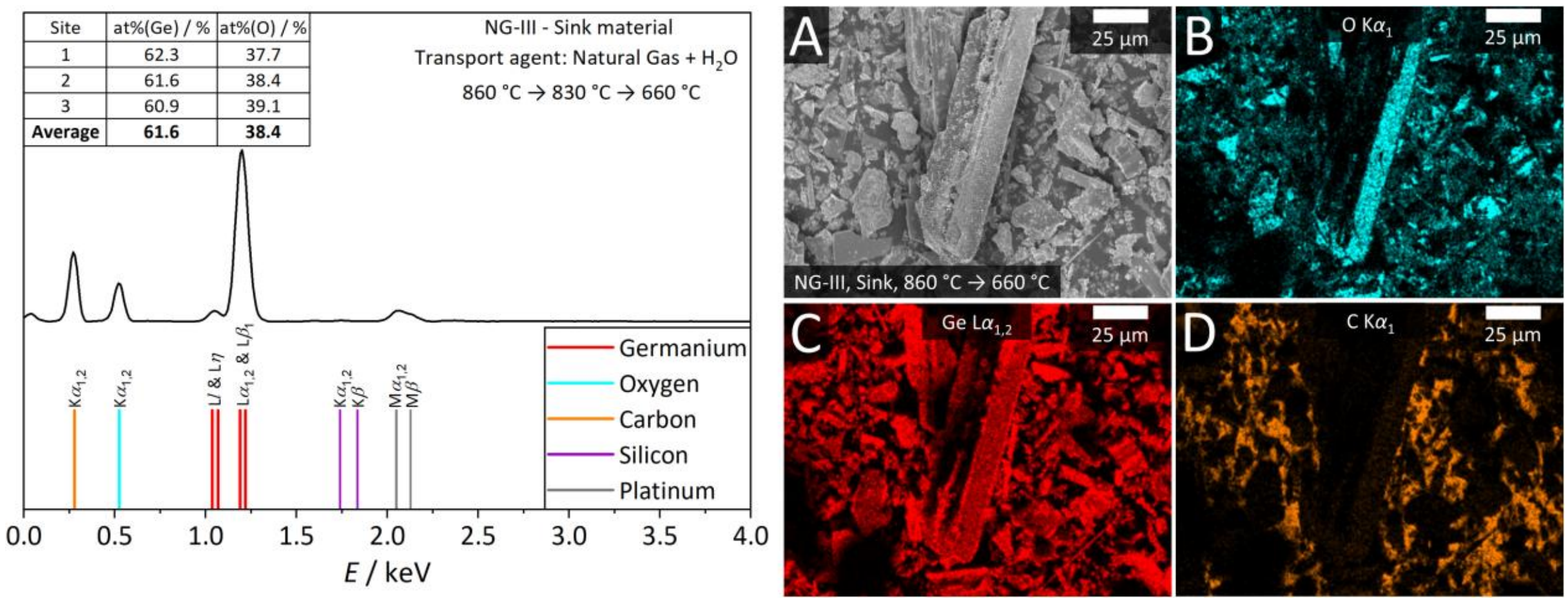

Figure S24 EDX data of the sink material of investigation NG-III is shown. An average at% value of germanium and oxygen is given. It is calculated from three different measuring spots. X-ray emission lines are shown for germanium, oxygen, carbon, silicon and platinum as references.[58] Additionally, for one measuring spot of the source material, a SEM image (A), as well as the elemental mappings for germanium (C, red dots), oxygen (B, blue dots) and carbon (D, orange dots) are shown.

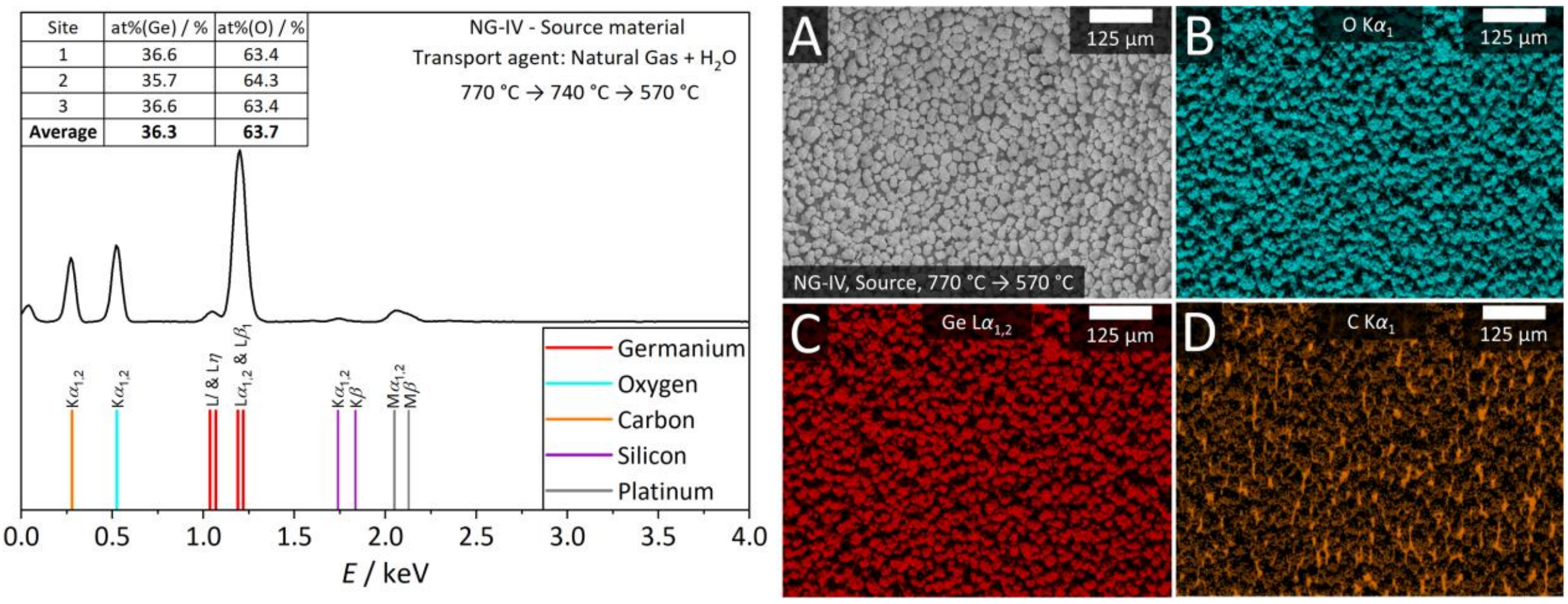

Figure S25 EDX data of the source material of investigation NG-IV is shown. An average at% value of germanium and oxygen is given. It is calculated from three different measuring spots. X-ray emission lines are shown for germanium, oxygen, carbon, silicon and platinum as references.[58] Additionally, for one measuring spot of the source material, a SEM image (A), as well as the elemental mappings for germanium (C, red dots), oxygen (B, blue dots) and carbon (D, orange dots) are shown.

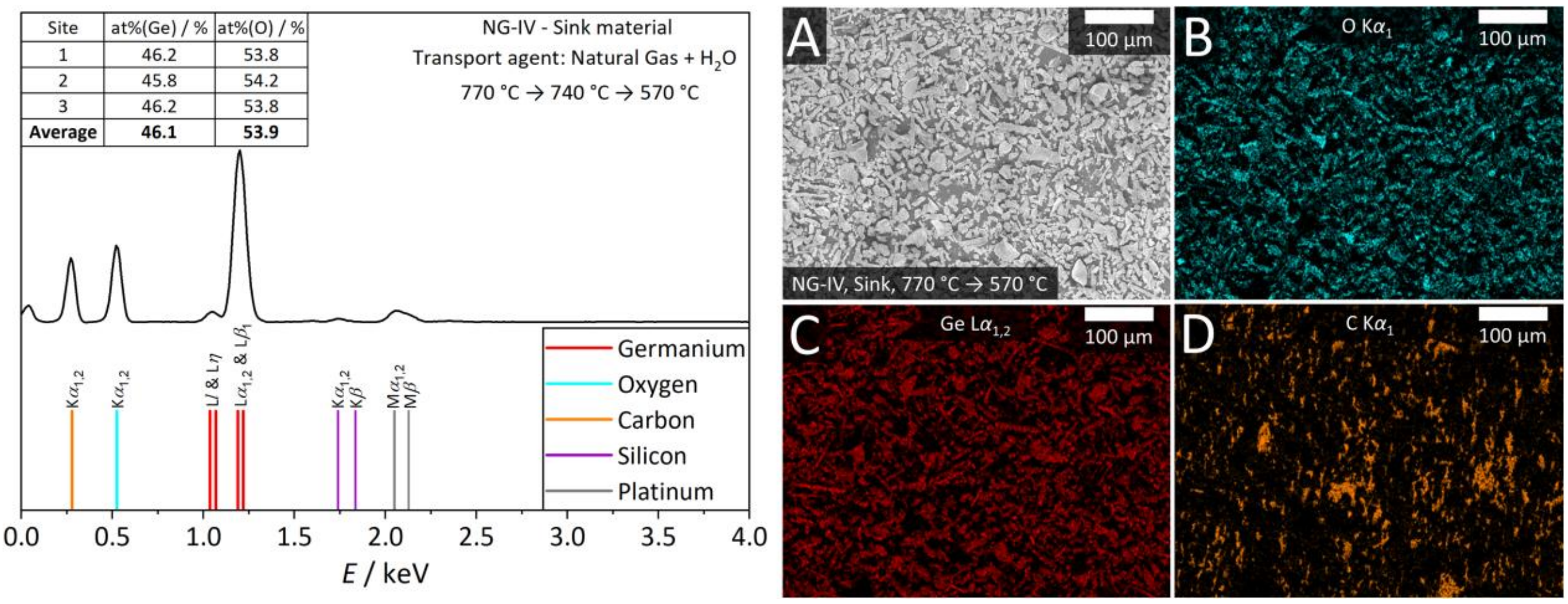

Figure S26 EDX data of the sink material of investigation NG-IV is shown. An average at% value of germanium and oxygen is given. It is calculated from three different measuring spots. X-ray emission lines are shown for germanium, oxygen, carbon, silicon and platinum as references.[58] Additionally, for one measuring spot of the source material, a SEM image (A), as well as the elemental mappings for germanium (C, red dots), oxygen (B, blue dots) and carbon (D, orange dots) are shown.

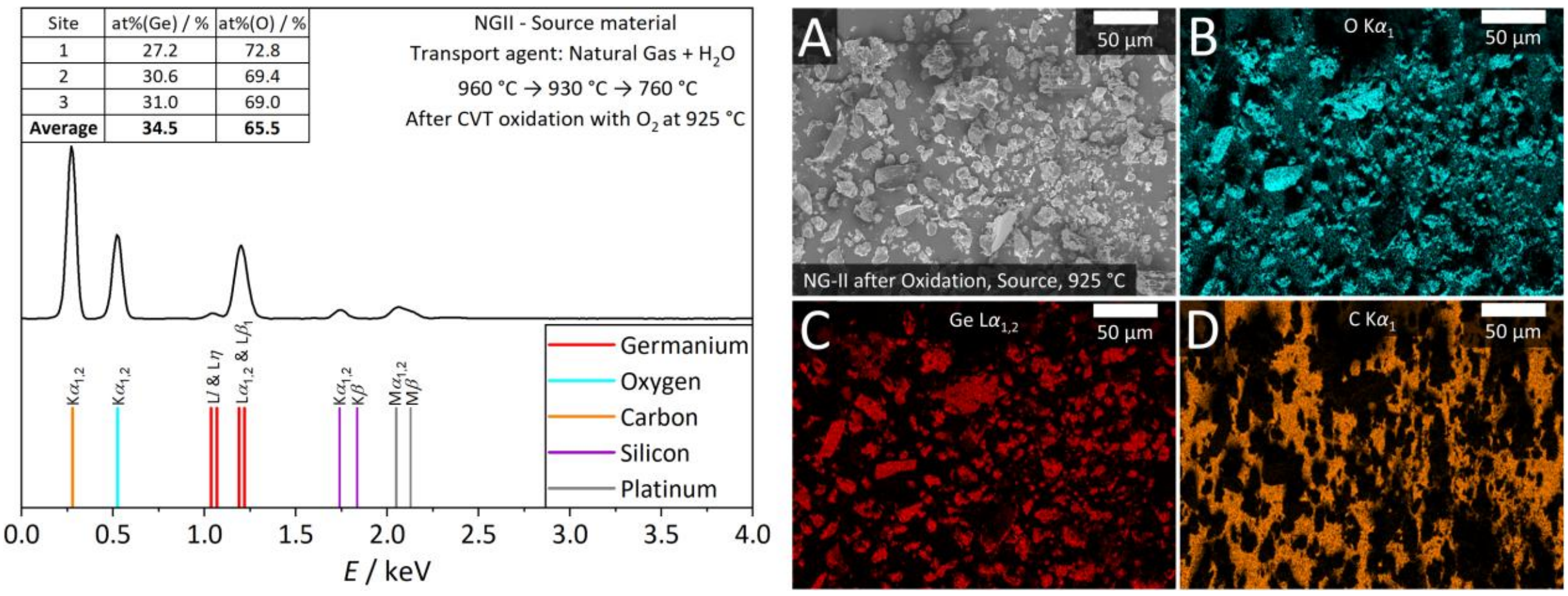

Figure S27 EDX data of the source material of investigation NG-II after oxidation with O_2 at 925 °C is shown. An average at% value of germanium and oxygen is given. It is calculated from three different measuring spots. X-ray emission lines are shown for germanium, oxygen, carbon, silicon and platinum as references.[58] Additionally, for one measuring spot of the source material, a SEM image (A), as well as the elemental mappings for germanium (C, red dots), oxygen (B, blue dots) and carbon (D, orange dots) are shown.

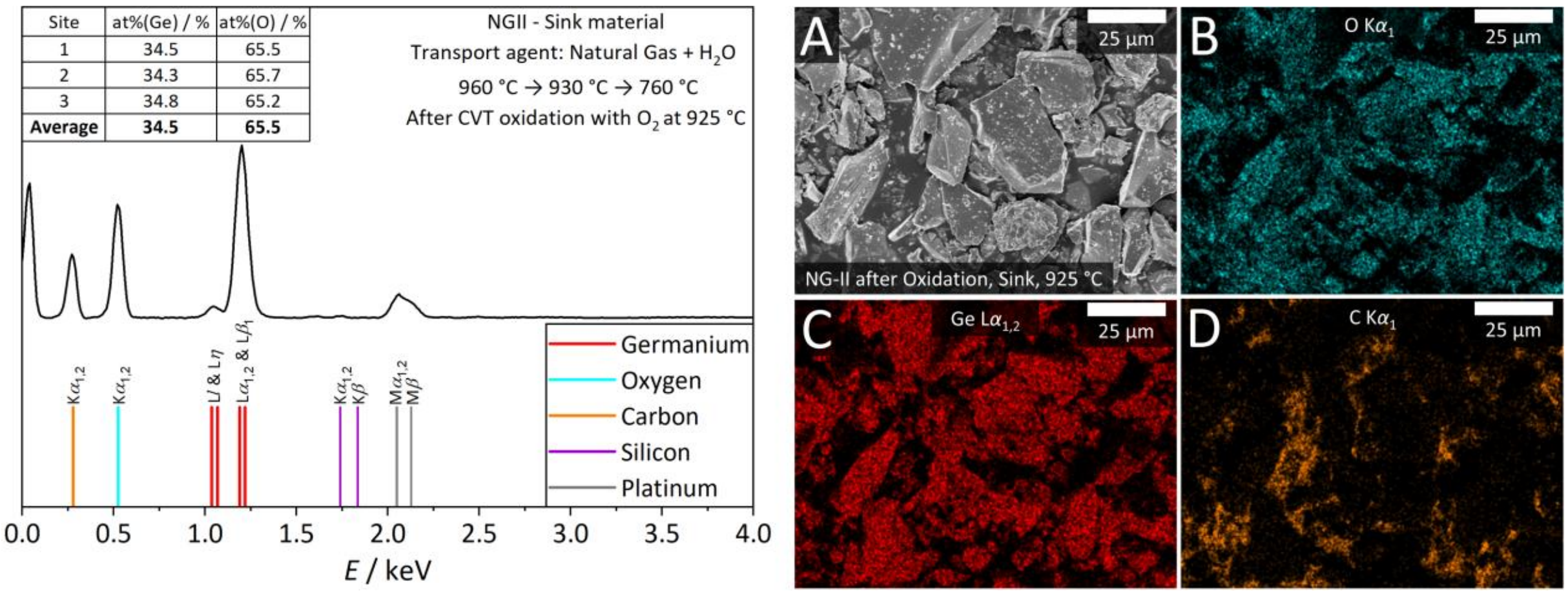

Figure S28 EDX data of the sink material of investigation NG-II after oxidation with O_2 at 925 °C is shown. An average at% value of germanium and oxygen is given. It is calculated from three different measuring spots. X-ray emission lines are shown for germanium, oxygen, carbon, silicon and platinum as references.[58] Additionally, for one measuring spot of the source material, a SEM image (A), as well as the elemental mapping for germanium (C, red dots), oxygen (B, blue dots) and carbon (D, orange dots) are shown.

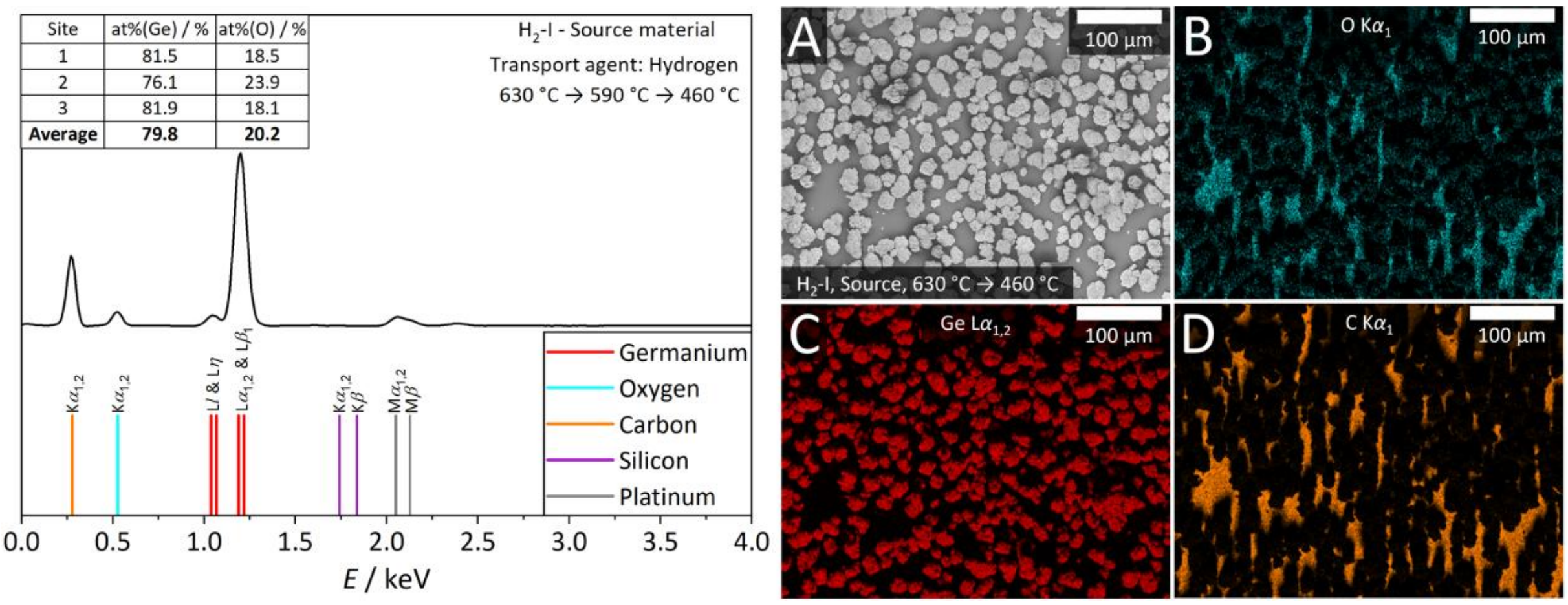

Figure S29 EDX data of the source material of investigation H_2-I is shown. An average at% value of germanium and oxygen is given. It is calculated from three different measuring spots. X-ray emission lines are shown for germanium, oxygen, carbon, silicon and platinum as references.[58] Additionally, for one measuring spot of the source material, a SEM image (A), as well as the elemental mappings for germanium (C, red dots), oxygen (B, blue dots) and carbon (D, orange dots) are shown.

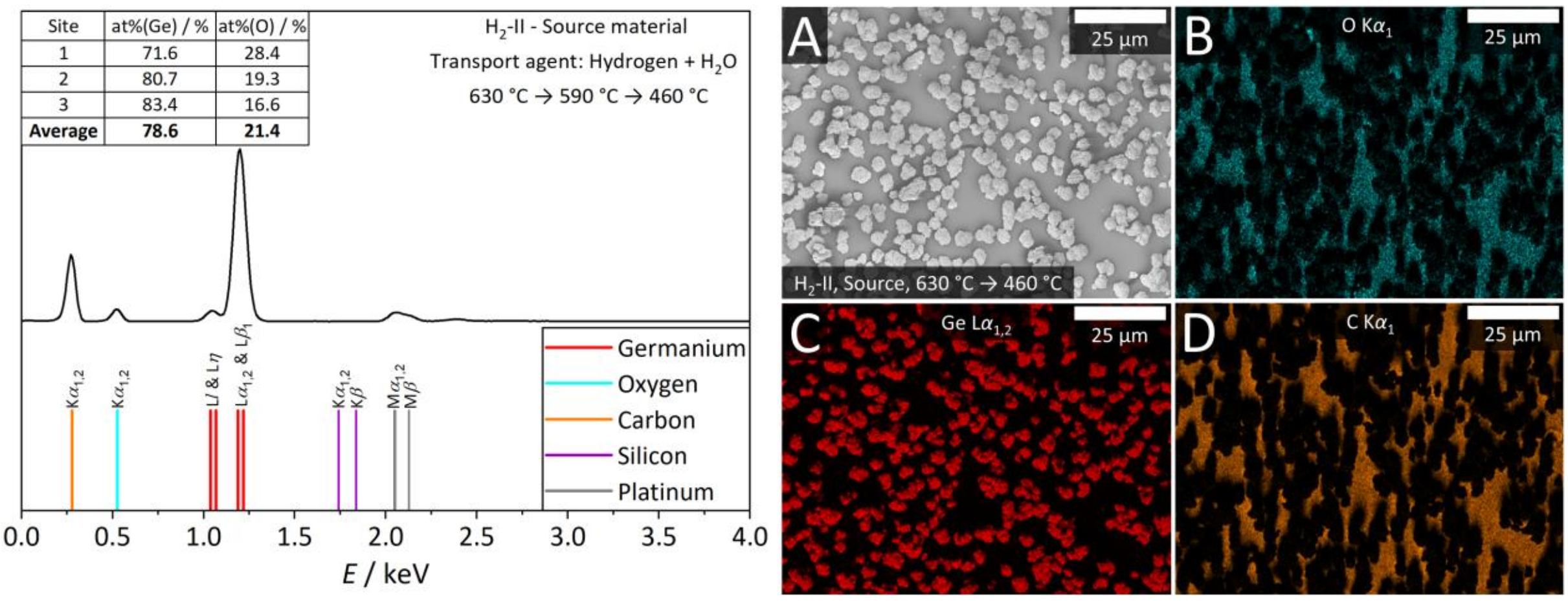

Figure S30 EDX data of the source material of investigation H_2-II is shown. An average at% value of germanium and oxygen is given. It is calculated from three different measuring spots. X-ray emission lines are shown for germanium, oxygen, carbon, silicon and platinum as references.[58] Additionally, for one measuring spot of the source material, a SEM image (A), as well as the elemental mappings for germanium (C, red dots), oxygen (B, blue dots) and carbon (D, orange dots) are shown.

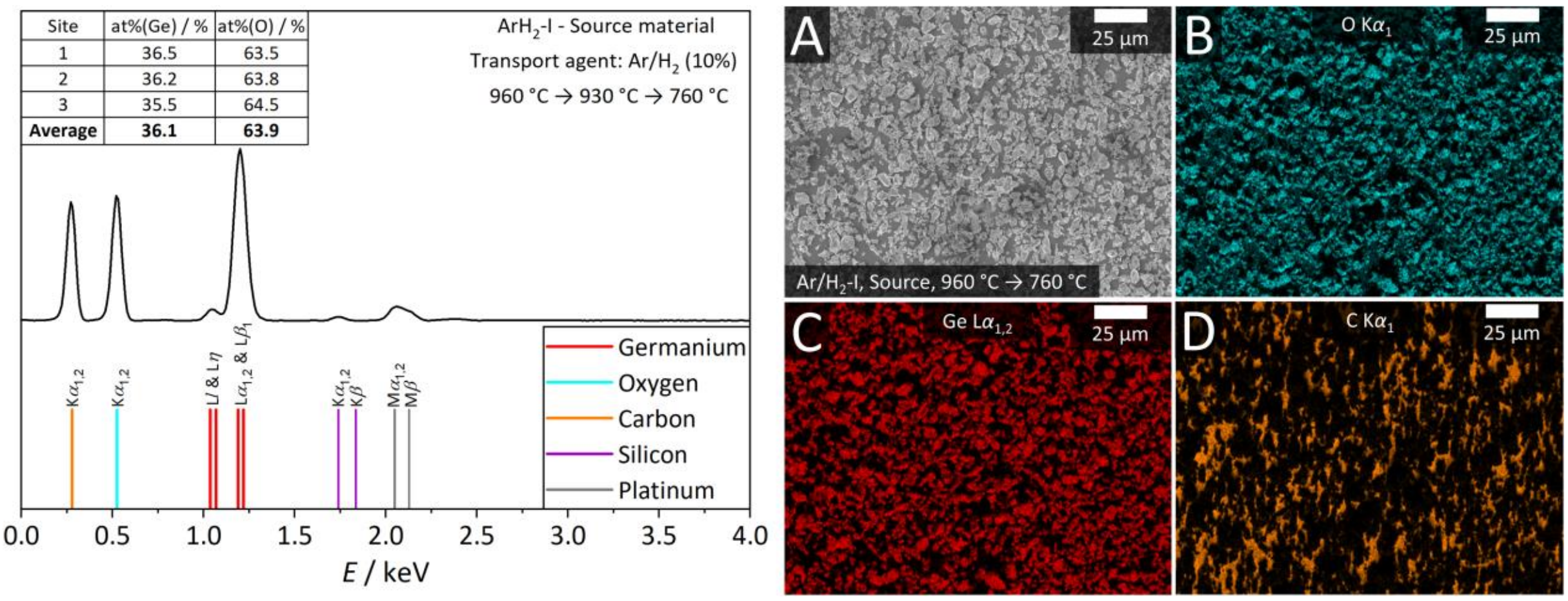

Figure S31 EDX data of the source material of investigation ArH$_2$-I is shown. An average at% value of germanium and oxygen is given. It is calculated from three different measuring spots. X-ray emission lines are shown for germanium, oxygen, carbon, silicon and platinum as references.[58] Additionally, for one measuring spot of the source material, a SEM image (A), as well as the elemental mappings for germanium (C, red dots), oxygen (B, blue dots) and carbon (D, orange dots) are shown.

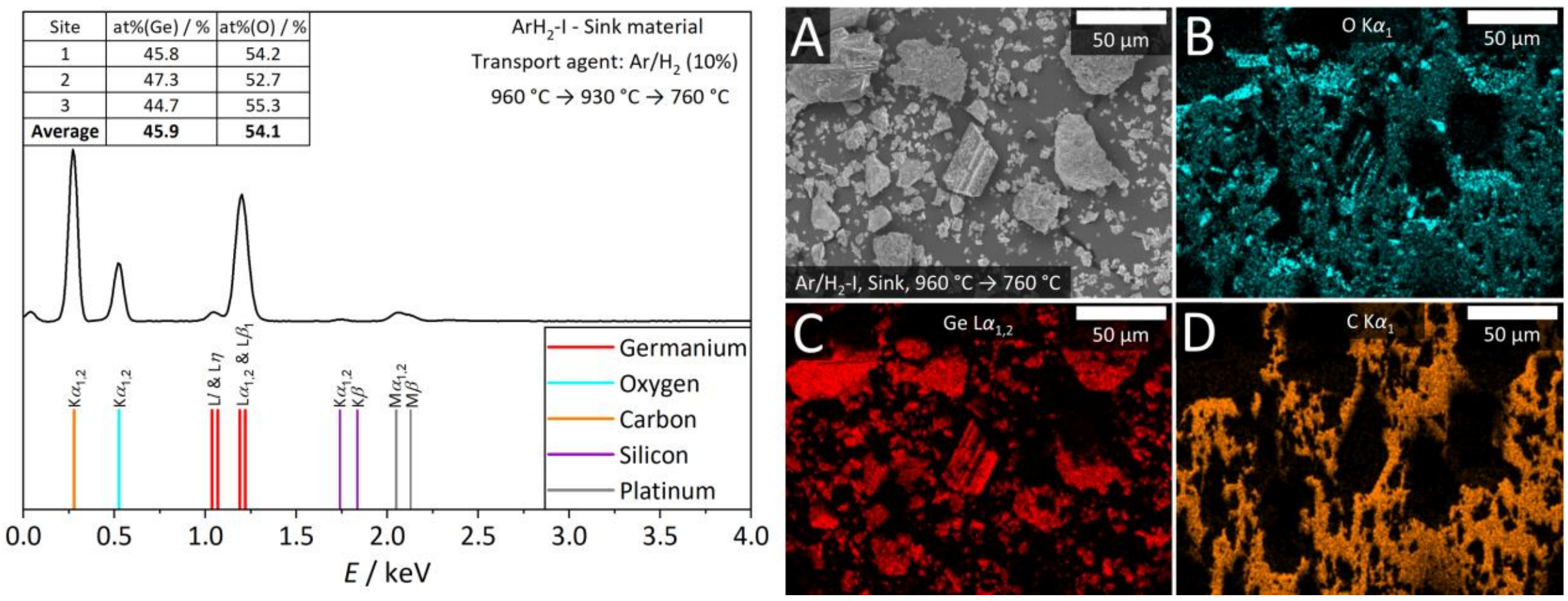

Figure S32 EDX data of the sink material of investigation ArH$_2$-I is shown. An average at% value of germanium and oxygen is given. It is calculated from three different measuring spots. X-ray emission lines are shown for germanium, oxygen, carbon, silicon and platinum as references.[58] Additionally, for one measuring spot of the source material, a SEM image (A), as well as the elemental mappings for germanium (C, red dots), oxygen (B, blue dots) and carbon (D, orange dots) are shown.

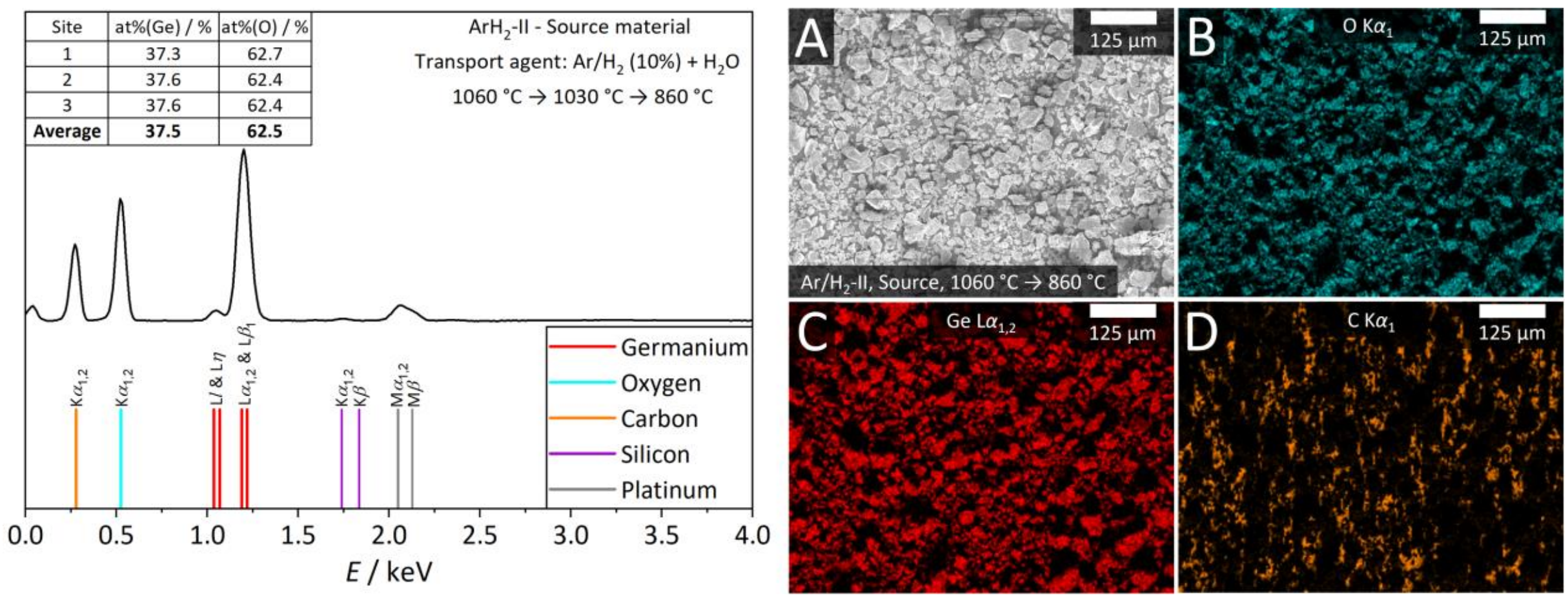

Figure S33 EDX data of the source material of investigation ArH$_2$-II is shown. An average at% value of germanium and oxygen is given. It is calculated from three different measuring spots. X-ray emission lines are shown for germanium, oxygen, carbon, silicon and platinum as references.[58] Additionally, for one measuring spot of the source material, a SEM image (A), as well as the elemental mappings for germanium (C, red dots), oxygen (B, blue dots) and carbon (D, orange dots) are shown.

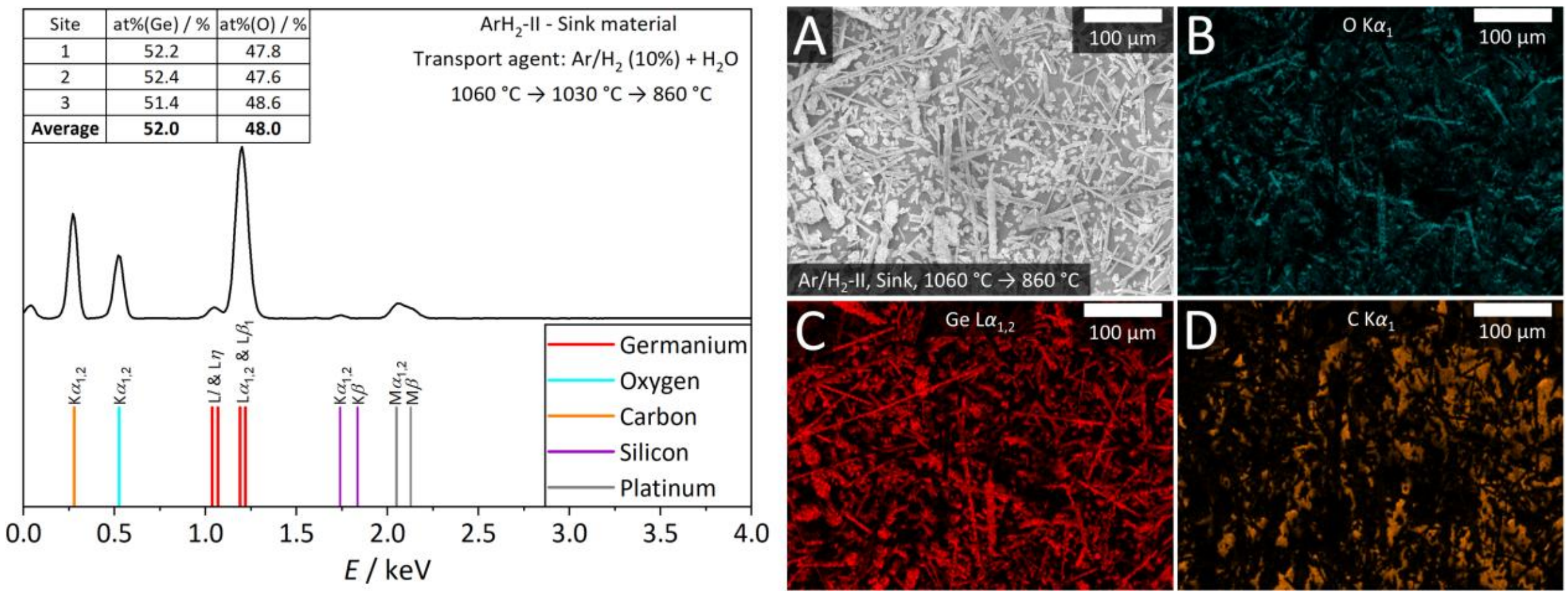

Site	at%(Ge) / %	at%(O) / %
1	52.2	47.8
2	52.4	47.6
3	51.4	48.6
**Average**	**52.0**	**48.0**

Figure S34 EDX data of the sink material of investigation ArH_2-II is shown. An average at% value of germanium and oxygen is given. It is calculated from three different measuring spots. X-ray emission lines are shown for germanium, oxygen, carbon, silicon and platinum as references.[58] Additionally, for one measuring spot of the source material, a SEM image (A), as well as the elemental mappings for germanium (C, red dots), oxygen (B, blue dots) and carbon (D, orange dots) are shown.

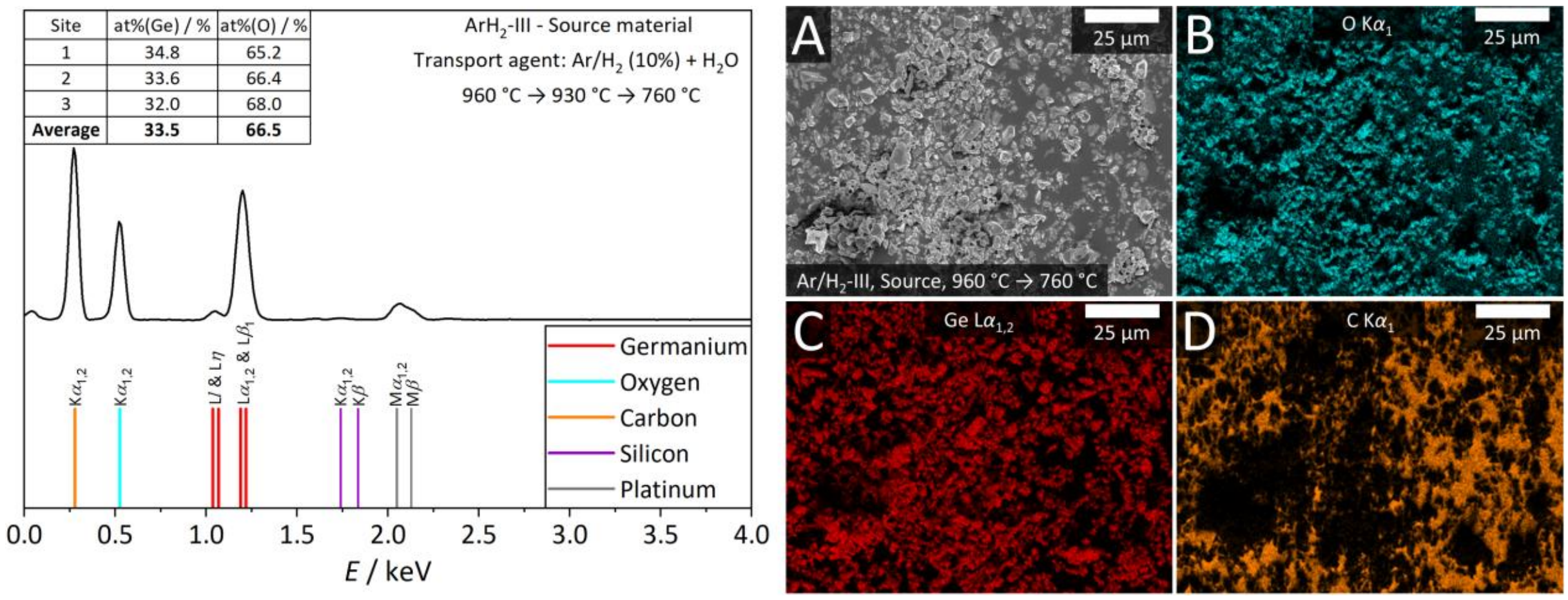

Site	at%(Ge) / %	at%(O) / %
1	34.8	65.2
2	33.6	66.4
3	32.0	68.0
**Average**	**33.5**	**66.5**

Figure S35 EDX data of the source material of investigation ArH_2-III is shown. An average at% value of germanium and oxygen is given. It is calculated from three different measuring spots. X-ray emission lines are shown for germanium, oxygen, carbon, silicon and platinum as references.[58] Additionally, for one measuring spot of the source material, a SEM image (A), as well as the elemental mappings for germanium (C, red dots), oxygen (B, blue dots) and carbon (D, orange dots) are shown.

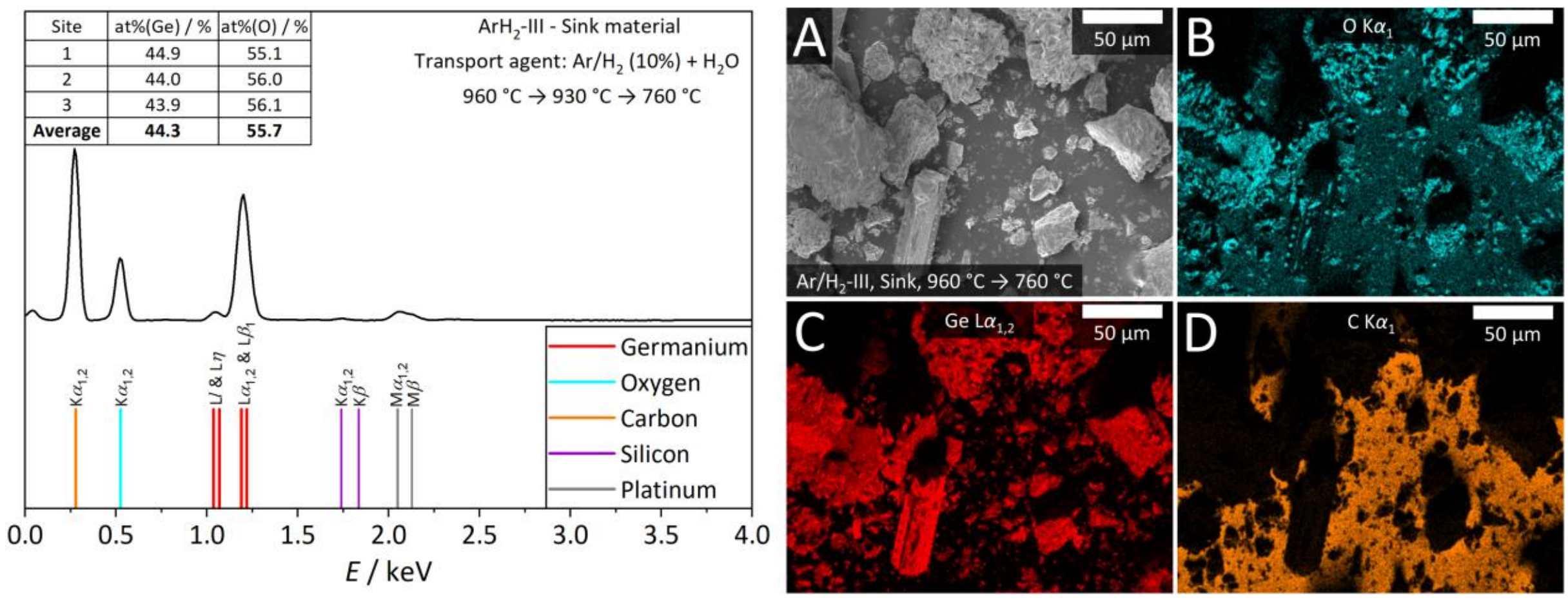

Site	at%(Ge) / %	at%(O) / %
1	44.9	55.1
2	44.0	56.0
3	43.9	56.1
**Average**	**44.3**	**55.7**

Figure S36 EDX data of the sink material of investigation ArH_2-III is shown. An average at% value of germanium and oxygen is given. It is calculated from three different measuring spots. X-ray emission lines are shown for germanium, oxygen, carbon, silicon and platinum as references.[58] Additionally, for one measuring spot of the source material, a SEM image (A), as well as the elemental mappings for germanium (C, red dots), oxygen (B, blue dots) and carbon (D, orange dots) are shown.

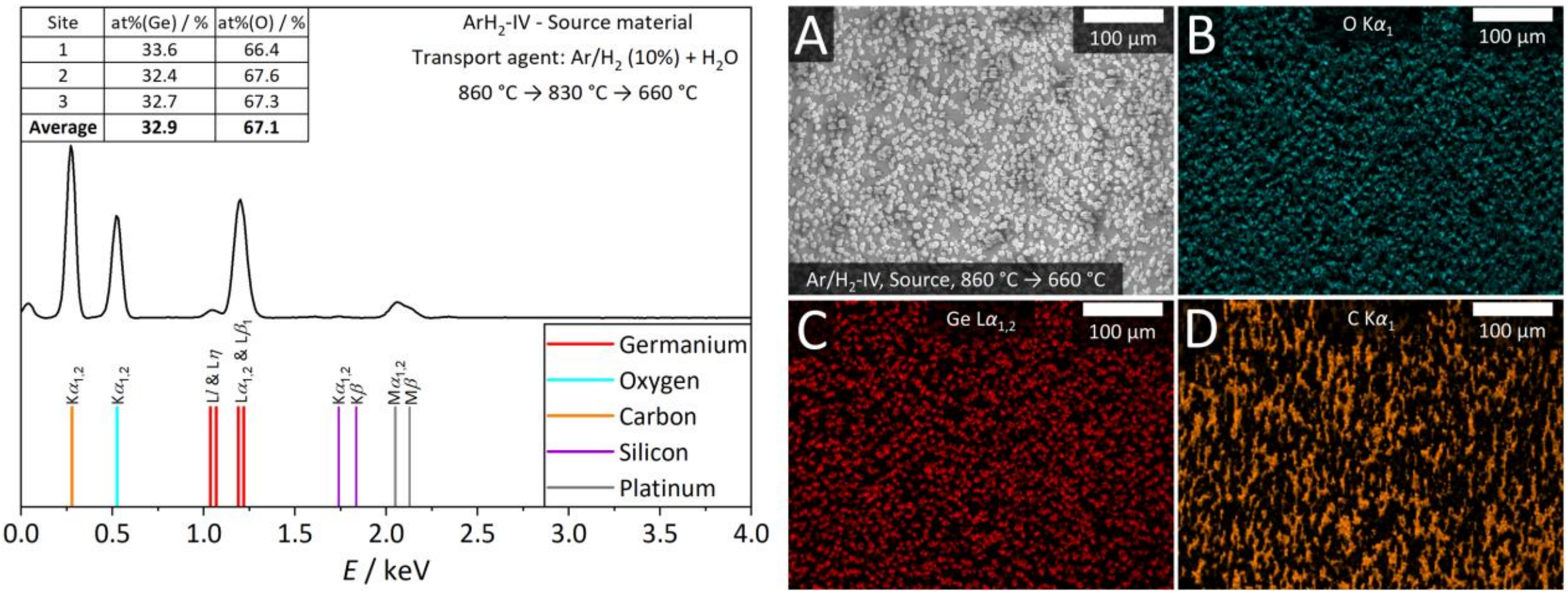

Figure S37 EDX data of the source material of investigation ArH_2-IV is shown. An average at% value of germanium and oxygen is given. It is calculated from three different measuring spots. X-ray emission lines are shown for germanium, oxygen, carbon, silicon and platinum as references.[58] Additionally, for one measuring spot of the source material, a SEM image (A), as well as the elemental mappings for germanium (C, red dots), oxygen (B, blue dots) and carbon (D, orange dots) are shown.

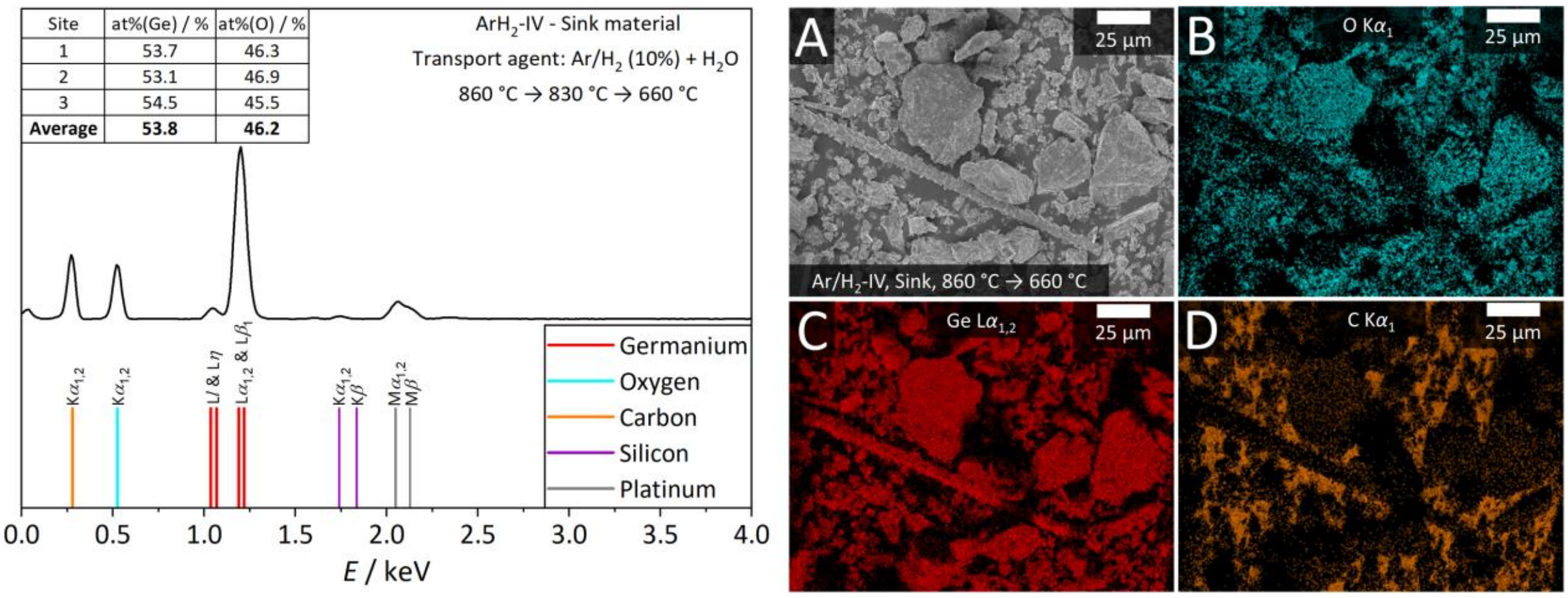

Figure S38 EDX data of the sink material of investigation ArH_2-IV is shown. An average at% value of germanium and oxygen is given. It is calculated from three different measuring spots. X-ray emission lines are shown for germanium, oxygen, carbon, silicon and platinum as references.[58] Additionally, for one measuring spot of the source material, a SEM image (A), as well as the elemental mappings for germanium (C, red dots), oxygen (B, blue dots) and carbon (D, orange dots) are shown.

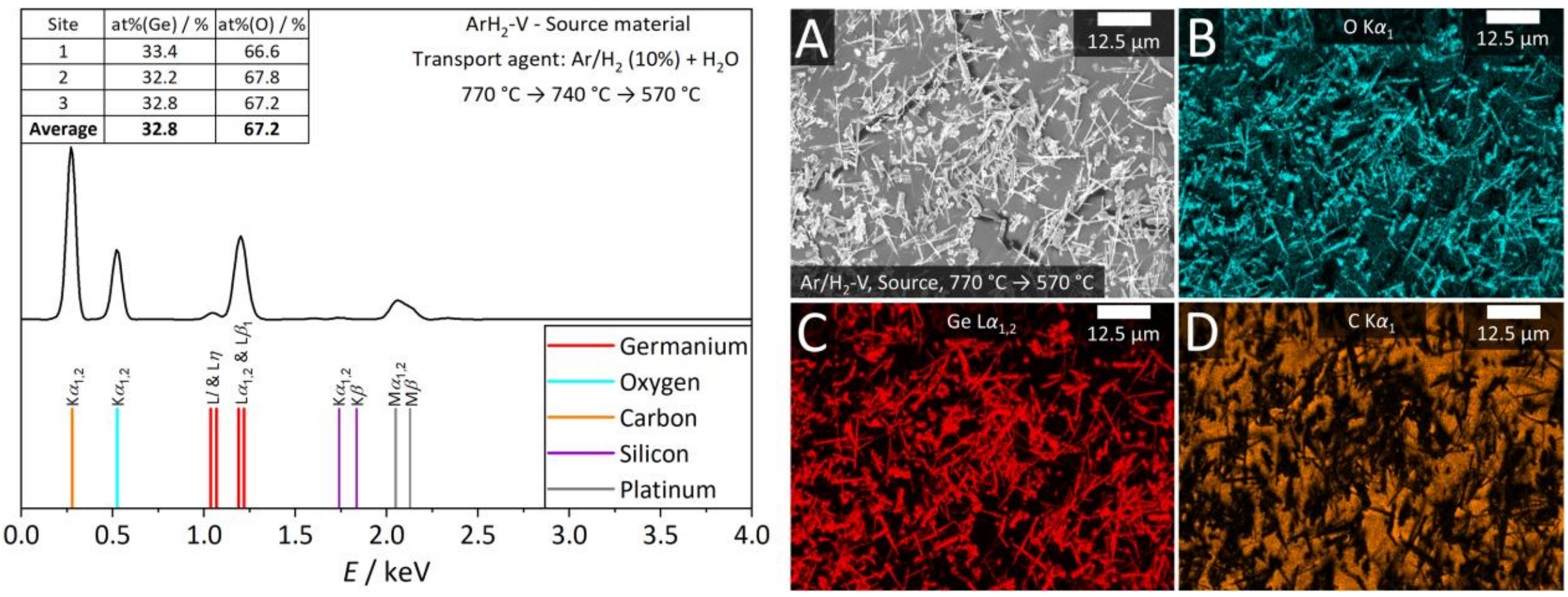

Figure S39 EDX data of the source material of investigation ArH_2-V is shown. An average at% value of germanium and oxygen is given. It is calculated from three different measuring spots. X-ray emission lines are shown for germanium, oxygen, carbon, silicon and platinum as references.[58] Additionally, for one measuring spot of the source material, a SEM image (A), as well as the elemental mappings for germanium (C, red dots), oxygen (B, blue dots) and carbon (D, orange dots) are shown.

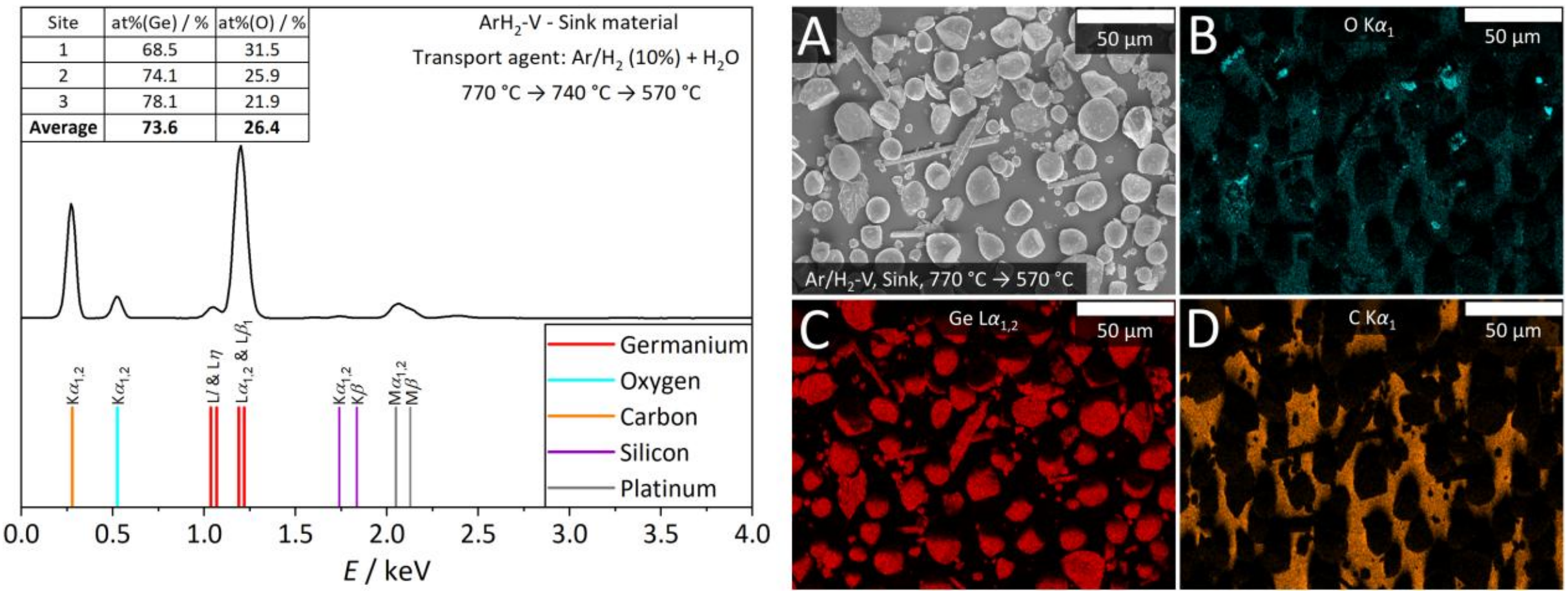

Figure S40 EDX data of the sink material of investigation ArH$_2$-V is shown. An average at% value of germanium and oxygen is given. It is calculated from three different measuring spots. X-ray emission lines are shown for germanium, oxygen, carbon, silicon and platinum as references.[58] Additionally, for one measuring spot of the source material, a SEM image (A), as well as the elemental mappings for germanium (C, red dots), oxygen (B, blue dots) and carbon (D, orange dots) are shown.

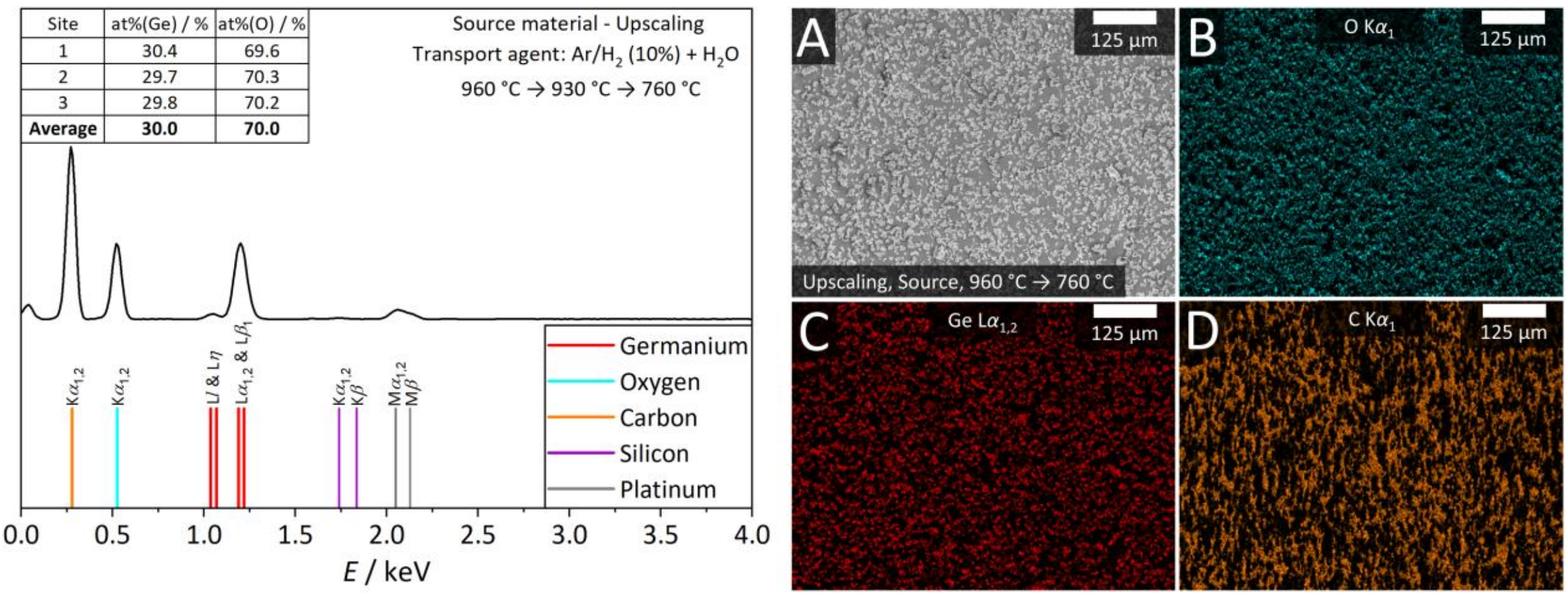

Figure S41 EDX data of the source material of the ArH$_2$-upscaling investigation is shown. An average at% value of germanium and oxygen is given. It is calculated from three different measuring spots. X-ray emission lines are shown for germanium, oxygen, carbon, silicon and platinum as references.[58] Additionally, for one measuring spot of the source material, a SEM image (A), as well as the elemental mappings for germanium (C, red dots), oxygen (B, blue dots) and carbon (D, orange dots) are shown.

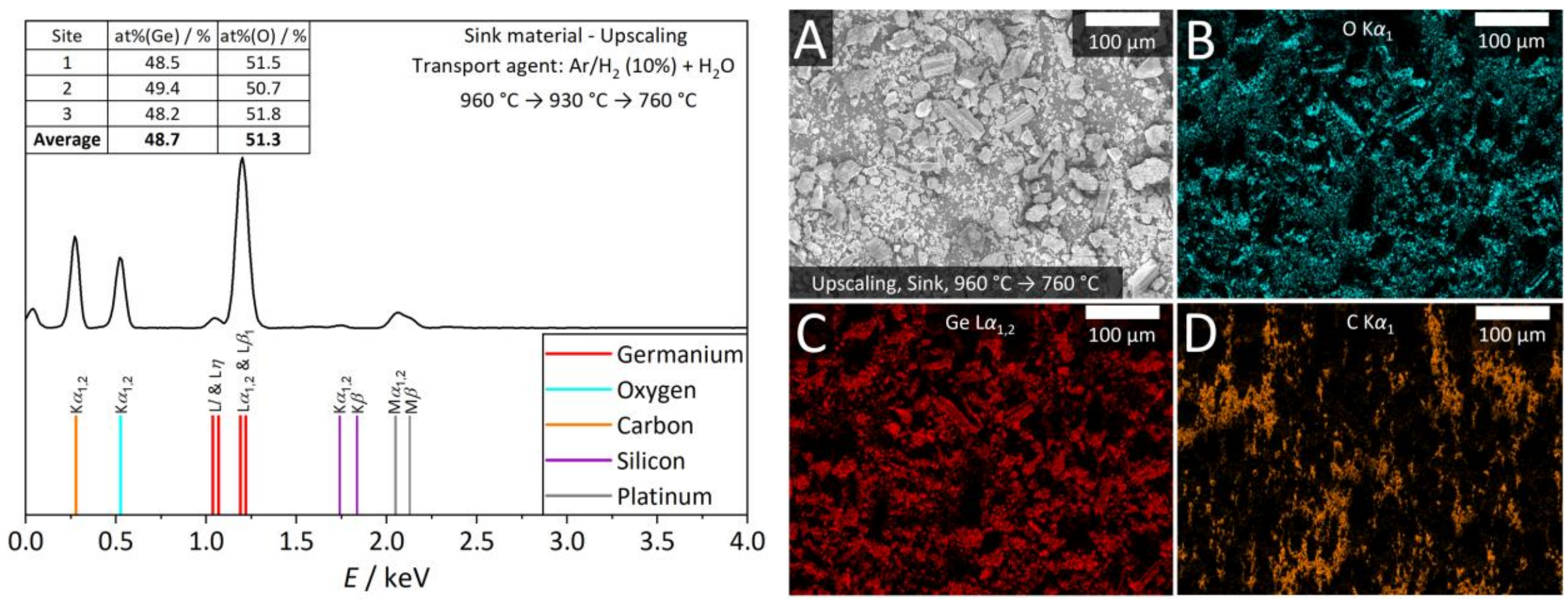

Figure S42 EDX data of the sink material of the ArH$_2$-upscaling investigation is shown. An average at% value of germanium and oxygen is given. It is calculated from three different measuring spots. X-ray emission lines are shown for germanium, oxygen, carbon, silicon and platinum as references.[58] Additionally, for one measuring spot of the source material, a SEM image (A), as well as the elemental mappings for germanium (C, red dots), oxygen (B, blue dots) and carbon (D, orange dots) are shown.

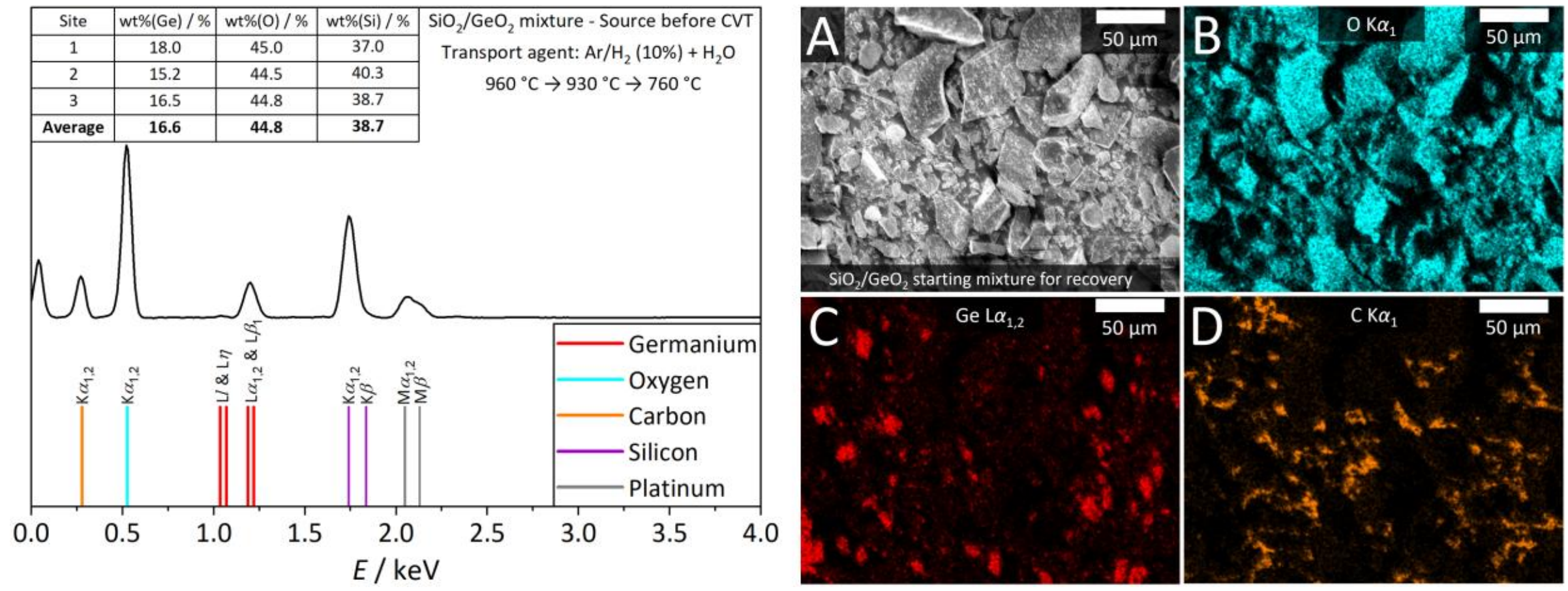

Figure S43 EDX data of the starting mixture of the SiO2/GeO_2 (15wt%) recovery investigation is shown. An average at% value of germanium, oxygen and silicon is given. It is calculated from three different measuring spots. X-ray emission lines are shown for germanium, oxygen, carbon, silicon and platinum as references.[58] Additionally, for one measuring spot of the source material, a SEM image (A), as well as the elemental mappings for germanium (C, red dots), oxygen (B, blue dots) and carbon (D, orange dots) are shown.

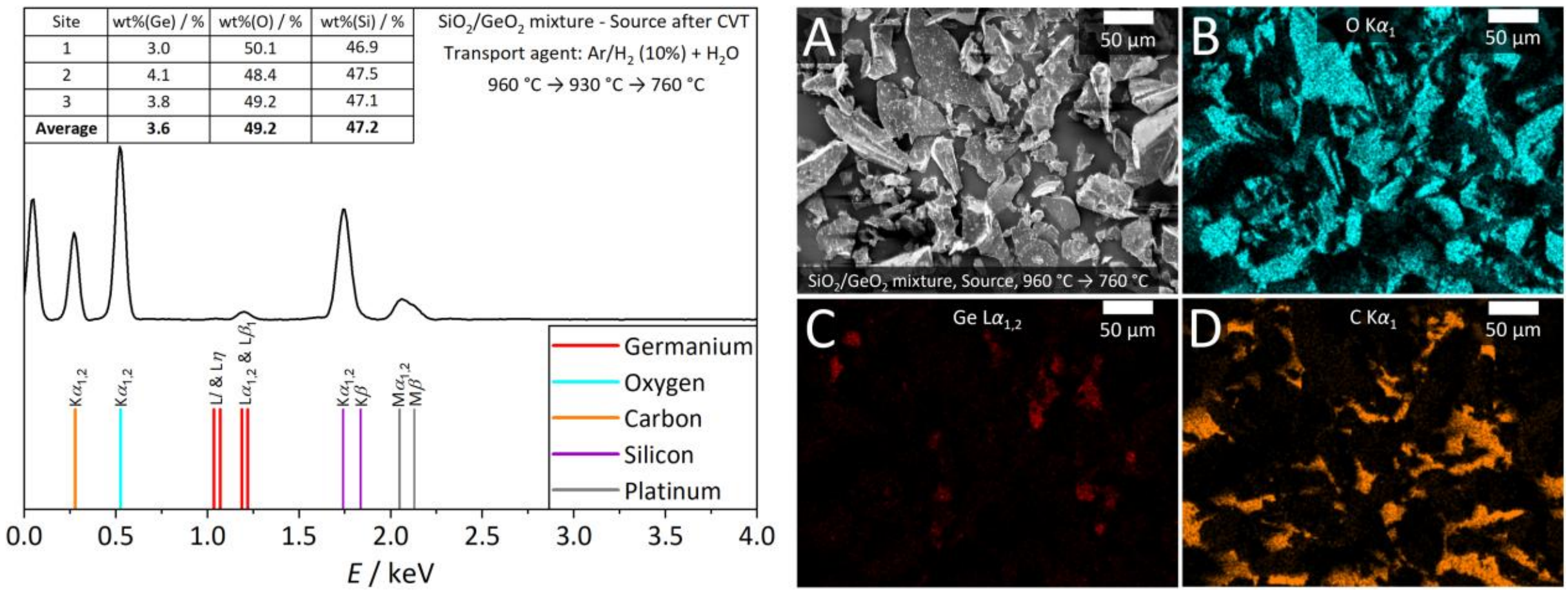

Figure S44 EDX data of the source of the SiO2/GeO_2 (15wt%) recovery investigation is shown. An average at% value of germanium, oxygen and silicon is given. It is calculated from three different measuring spots. X-ray emission lines are shown for germanium, oxygen, carbon, silicon and platinum as references.[58] Additionally, for one measuring spot of the source material, a SEM image (A), as well as the elemental mappings for germanium (C, red dots), oxygen (B, blue dots) and carbon (D, orange dots) are shown.

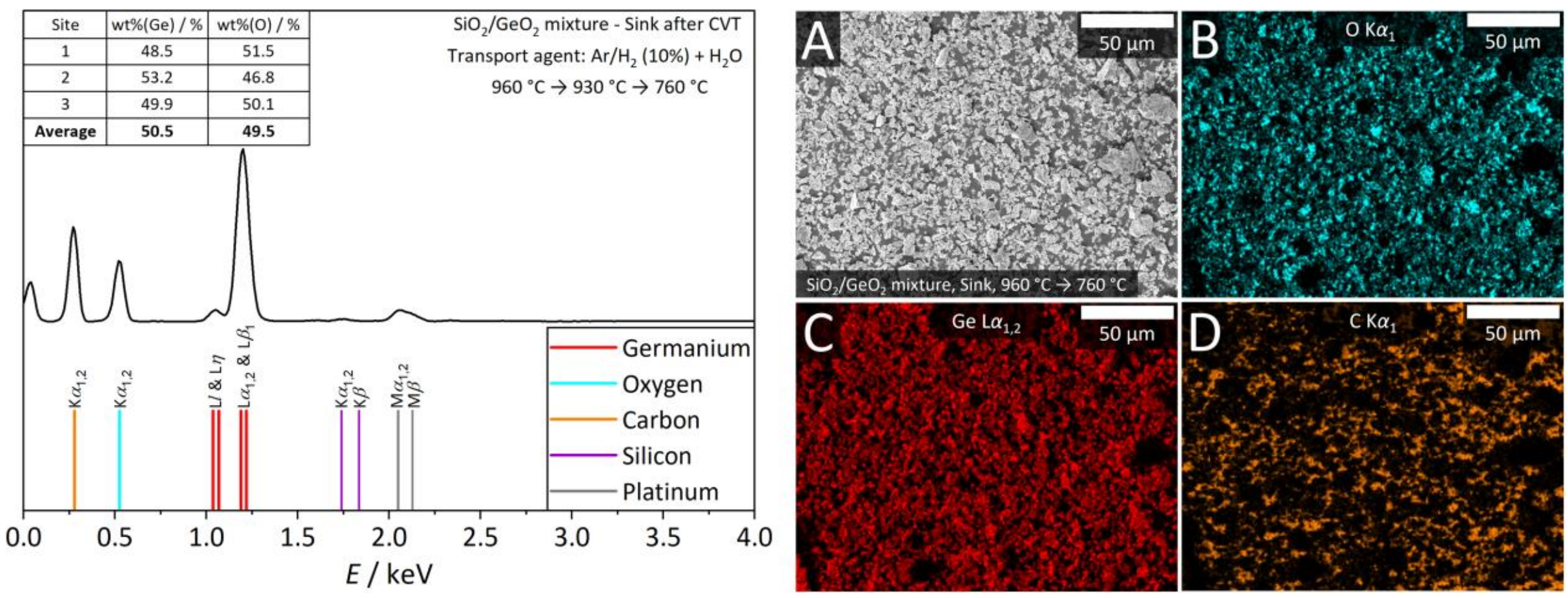

Figure S45 EDX data of the sink of the SiO2/GeO_2 (15wt%) recovery investigation is shown. An average at% value of germanium and oxygen is given. It is calculated from three different measuring spots. X-ray emission lines are shown for germanium, oxygen, carbon, silicon and platinum as references.[58] Additionally, for one measuring spot of the source material, a SEM image (A), as well as the elemental mappings for germanium (C, red dots), oxygen (B, blue dots) and carbon (D, orange dots) are shown.

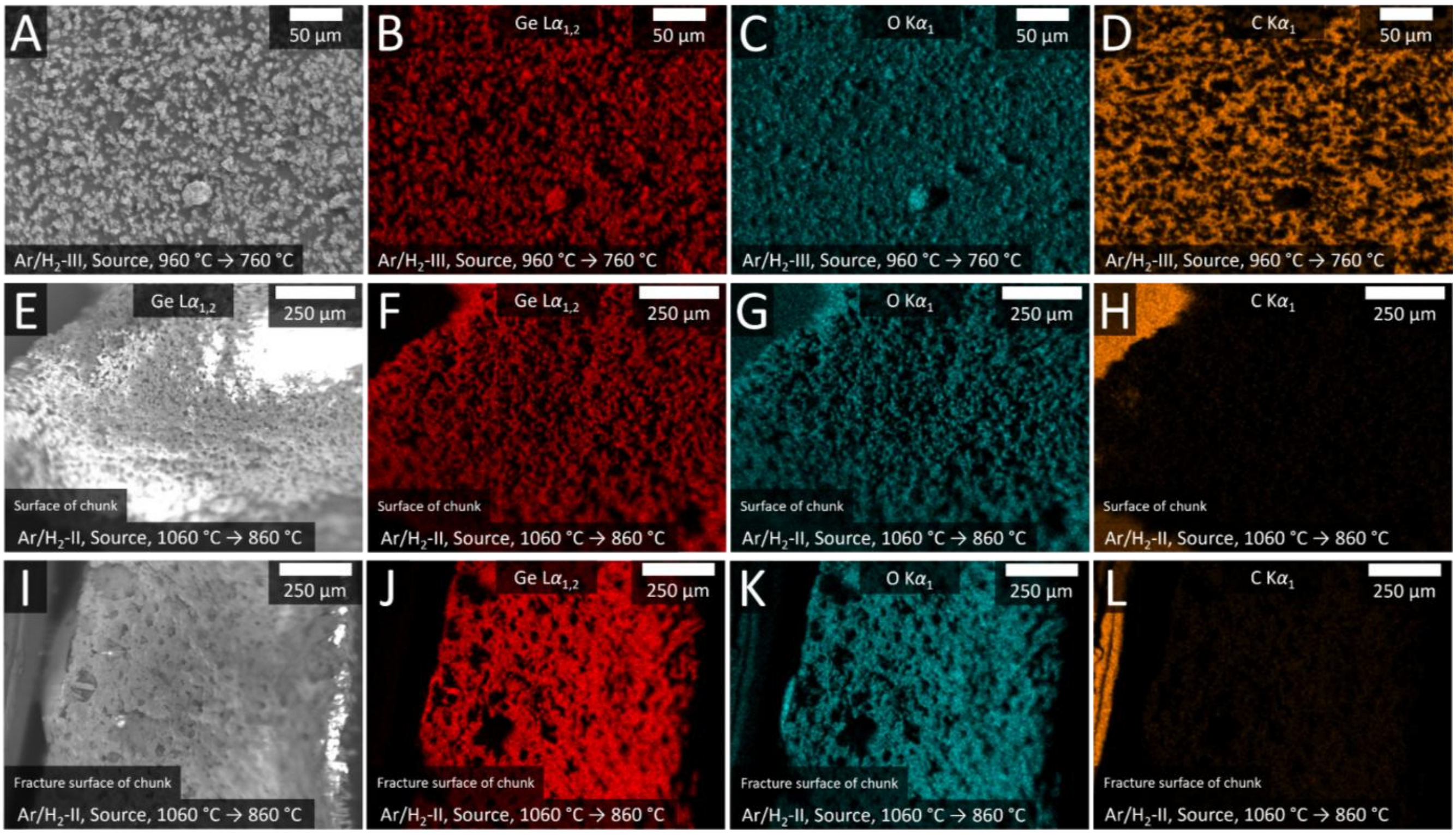

Figure S46 Morphological comparisons of unground source material from investigations ArH_2-III (A-D, 960 °C → 760 °C) and ArH_2-II (E-L, 1060 °C → 860 °C). SEM images are shown for the powdery source material of ArH_2-III (A) and for the chunk-like source material of ArH_2-II as both a surface image (E) and fracture surface image (I). In addition, elemental mappings for germanium (B, F, J, red dots), oxygen (C, G, K, blue dots) and carbon (D, H, L, orange dots) are shown.

During the mechanical removal of the source and sink material, small silica particles from the reaction tube were mixed into the collected material. The elemental mapping of silicon revealed no homogenous distribution (see Figure S44-S48C), which would be expected if silicon had been transported via CVT. Overall, the average silicon content was 1.4 weight percentage (wt%).

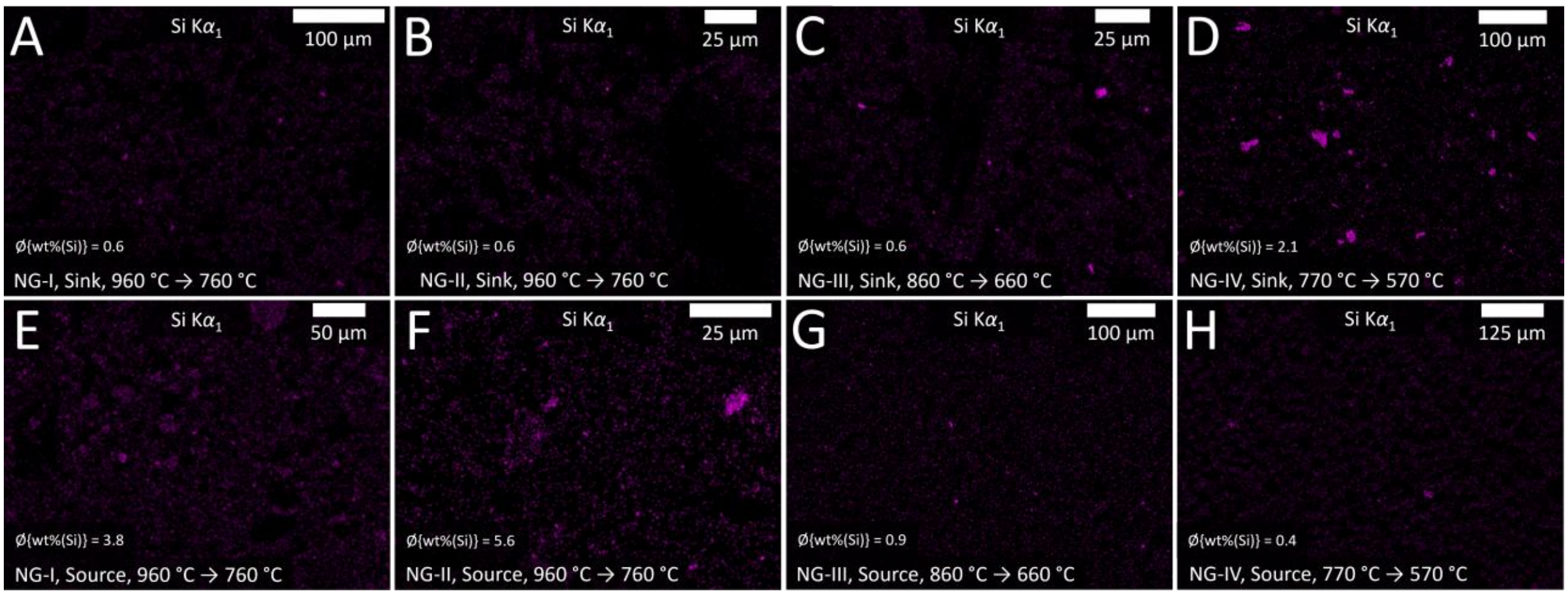

Figure S47 EDX elemental mapping of silicon for all NG investigations is shown. In addition, the average wt% of silicon calculated from three different measuring spots at the sink (A-D) and the source (E-H) is given. The shown measuring spots correspond to the shown measuring spots of germanium, oxygen and carbon, which are used in other graphics.

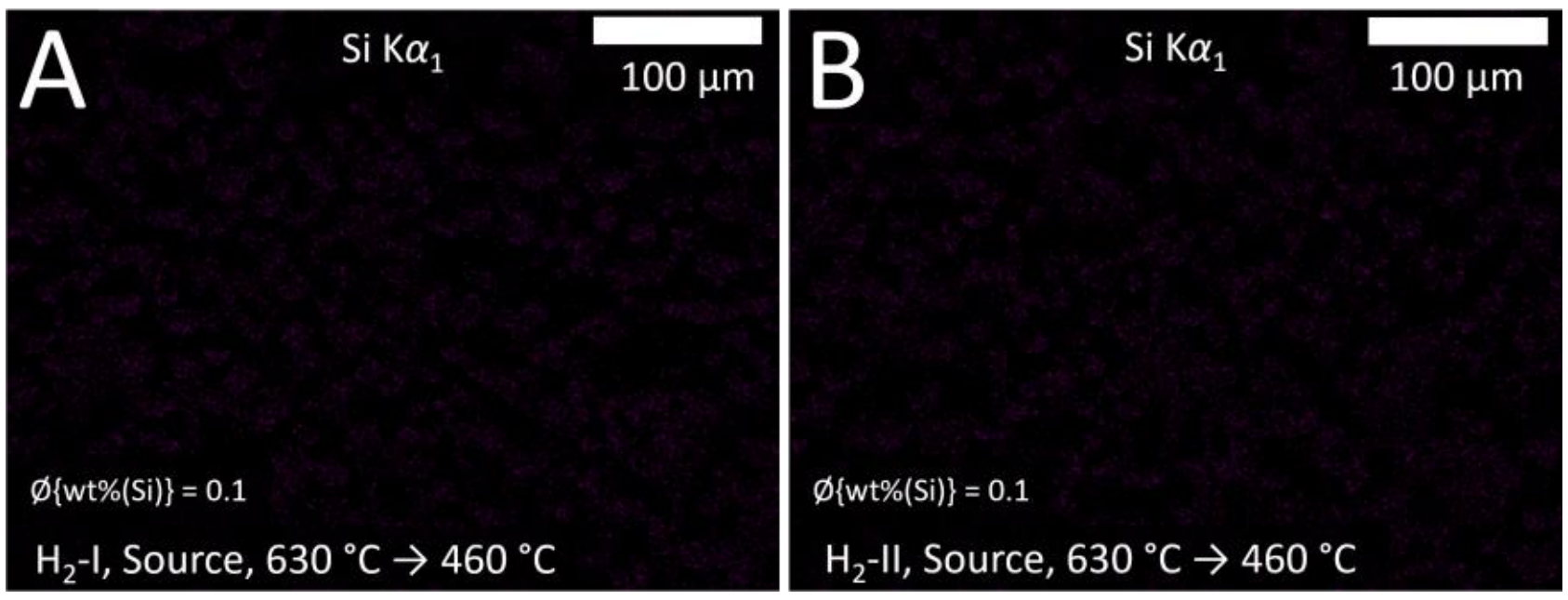

Figure S48 EDX elemental mapping of silicon for all H_2 investigations is shown. In addition, the average wt% of silicon calculated from three different measuring spots at the source (A & B) is given. The shown measuring spots correspond to the shown measuring spots of germanium, oxygen and carbon, which are used in other graphics.

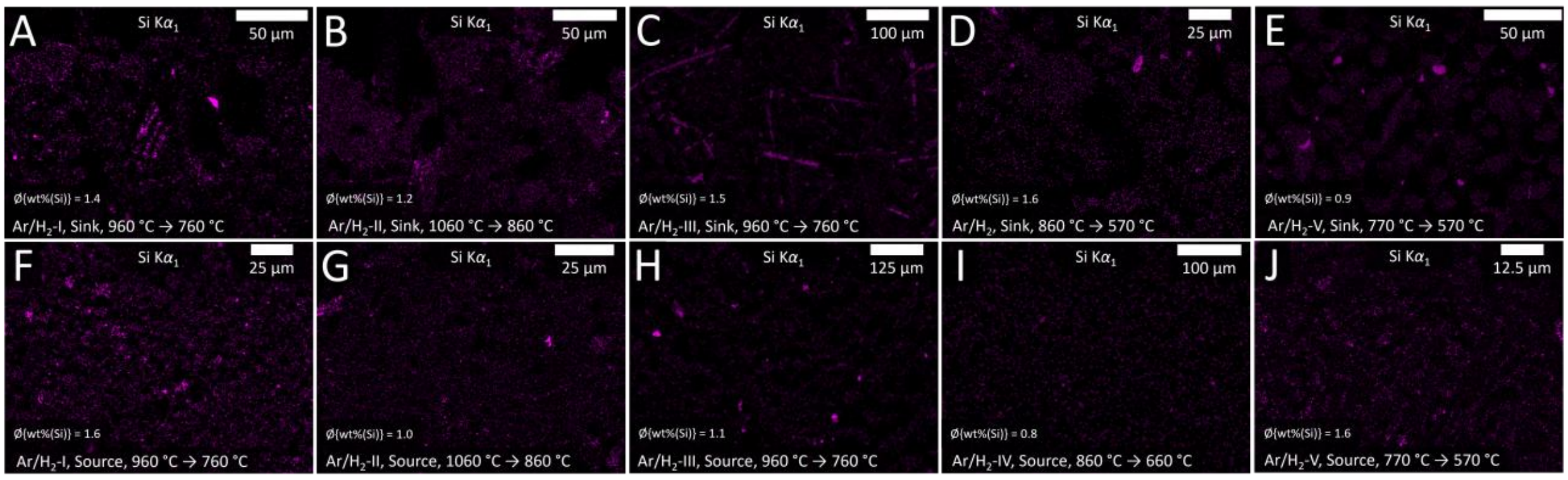

Figure S49 EDX elemental mapping of silicon for all ArH_2 investigations is shown. In addition, the average wt% of silicon calculated from three different measuring spots at the sink (A-E) and the source (F-J) is given. The shown measuring spots correspond to the shown measuring spots of germanium, oxygen and carbon, which are used in other graphics.

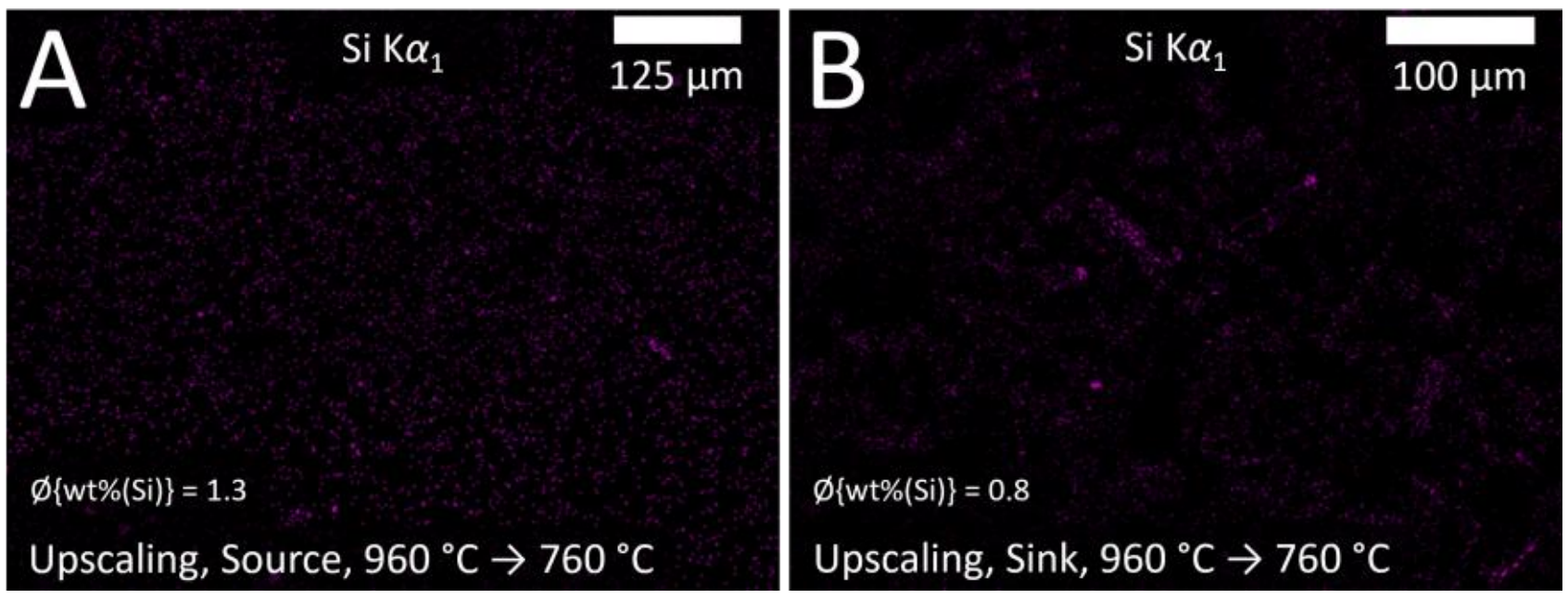

Figure S50 EDX elemental mapping of silicon for the upscaling investigation is shown. In addition, the average wt% of silicon calculated from three different measuring spots at the source (A) and the sink (B) is given. The shown measuring spots correspond to the shown measuring spots of germanium, oxygen and carbon, which are used in other graphics.

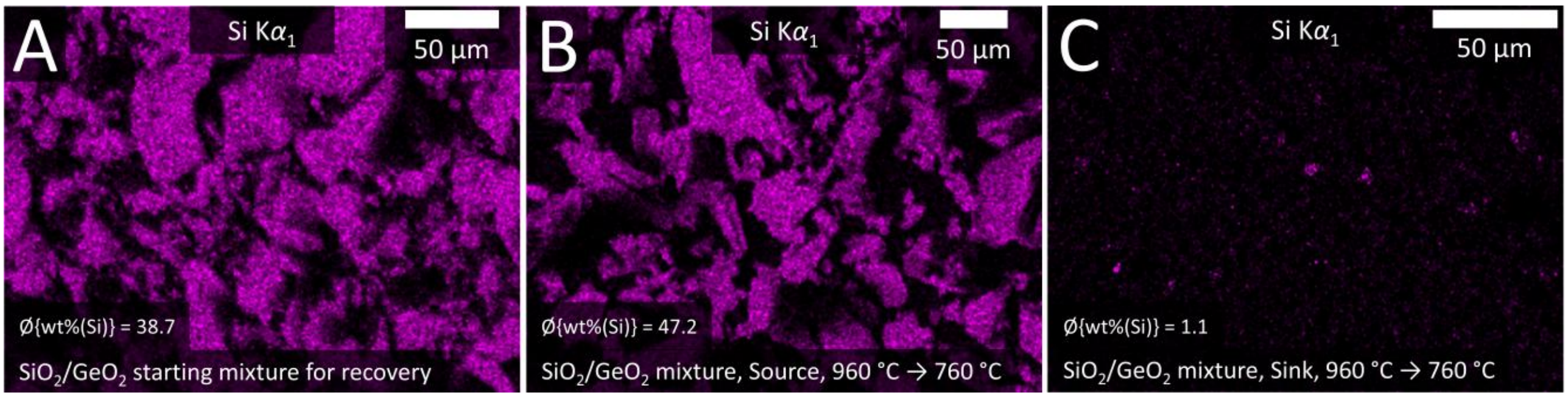

Figure S51 EDX elemental mapping of silicon for the recovery investigations starting with a SiO_2/GeO_2 (15wt%) mixture is shown. In addition, the average wt% of silicon calculated from three different measuring spots at the SiO_2/GeO_2 starting material for the recovery (A), the source (B) and the sink (C) is given. The shown measuring spots correspond to the shown measuring spots of germanium, oxygen and carbon, which are used in other graphics.

7. Comparison natural gas and hydrogen(10%)-argon(90%) mixture

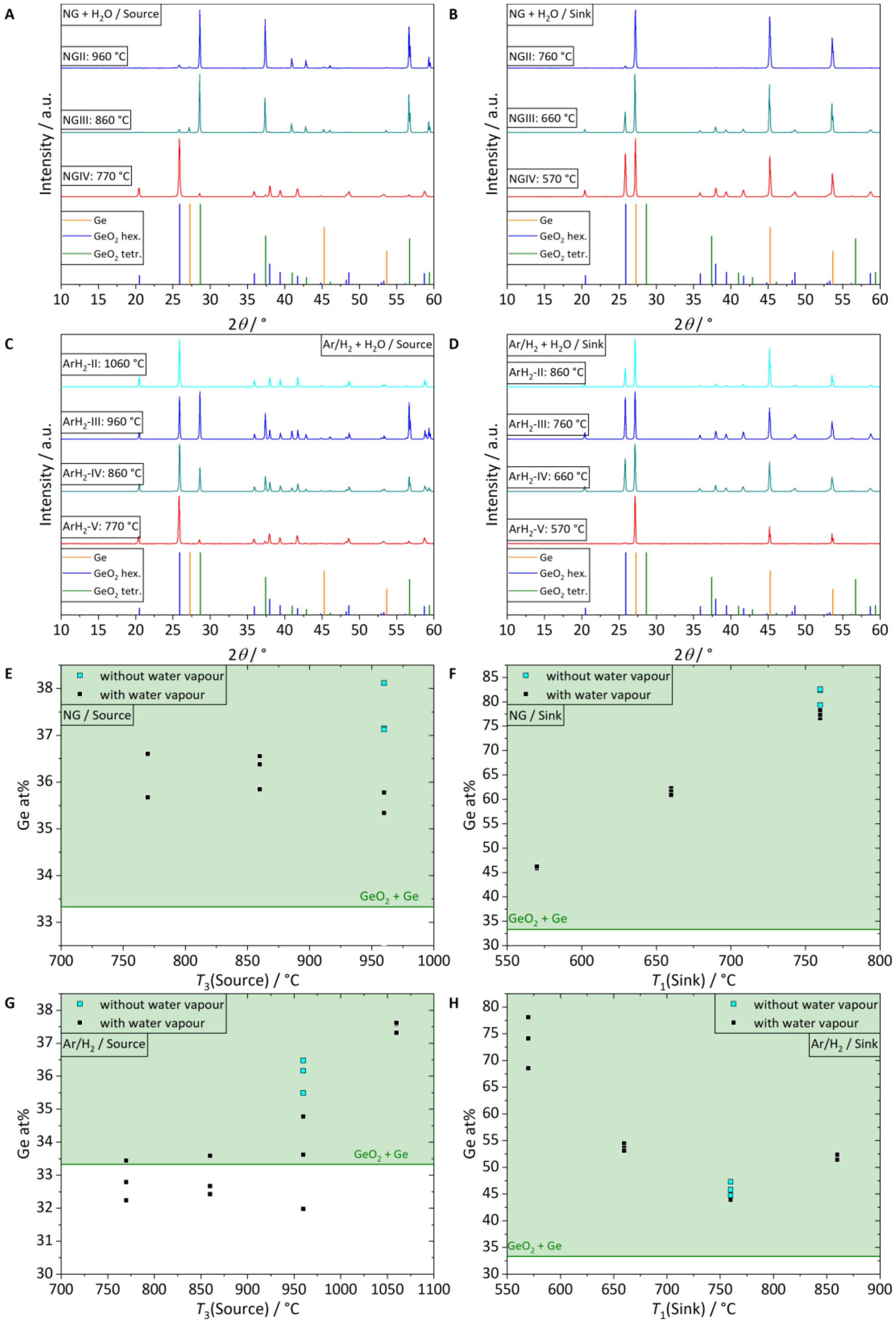

Figure S52 Comparisons of XRD (A-D) and EDX (E-H) data of the phase composition at sink and source after the CVT performed with either natural gas (A&B, E&F) or the hydrogen(10%)-argon(90%) mixture (C&D, G&H) are shown. For the XRD data, references for germanium[55], hexagonal germanium dioxide[56] and tetragonal germanium dioxide[57] are shown.

8. References

49 van't Hoff, J.H. (1884) Études de dynamique chimique, F. Muller & Company, Amsterdam.
50 Binnewies, M. and Milke, E. (2002) Thermochemical Data of Elements and Compounds, 2nd edn, Wiley, Weinheim.
51 Chase, M. W. (1998) NIST-JANAF Thermochemical Tables, 4th edn, American Institute of Physics, New York.
52 Knacke, O., Kubaschewski, O., Hesselmann, K. (1991) Thermochemical Properties of Inorganic Substances, Springer, Berlin.
55 Smakula, A. and Kalnajs, J. (1955) Precision Determination of Lattice Constants with a Geiger-Counter X-Ray Diffractometer. Physical Review, **99** (6), 1737–1743 doi: 10.1103/PhysRev.99.1737.
56 Uno, R., Ozawa, H., Yamanaka, T., et al. (1988) Powder Diffractometry at the Tsukuba Photon Factory. Australian Journal of Physics, **41** (2), 133 doi: 10.1071/PH880133.
57 Haines, J., Léger, J.M., Chateau, C., Pereira, A.S. (2000) Structural evolution of rutile-type and CaCl 2 -type germanium dioxide at high pressure. Physics and Chemistry of Minerals, **27** (8), 575–582 doi: 10.1007/s002690000092.
58 Bearden, J.A. (1967) X-Ray Wavelengths. Reviews of Modern Physics, **39** (1), 78–124 doi: 10.1103/RevModPhys.39.78.
